# Electrochemical Benzylic C(sp^3^)–H Acyloxylation

**DOI:** 10.1021/acs.orglett.2c01930

**Published:** 2022-07-13

**Authors:** Alexander P. Atkins, Albert C. Rowett, David M. Heard, Joseph A. Tate, Alastair J. J. Lennox

**Affiliations:** † University of Bristol, School of Chemistry, Cantock’s Close, Bristol BS8 1TS, United Kingdom; ‡ Syngenta, Jealott’s Hill International Research Centre, Bracknell RG42 6EY, United Kingdom

## Abstract

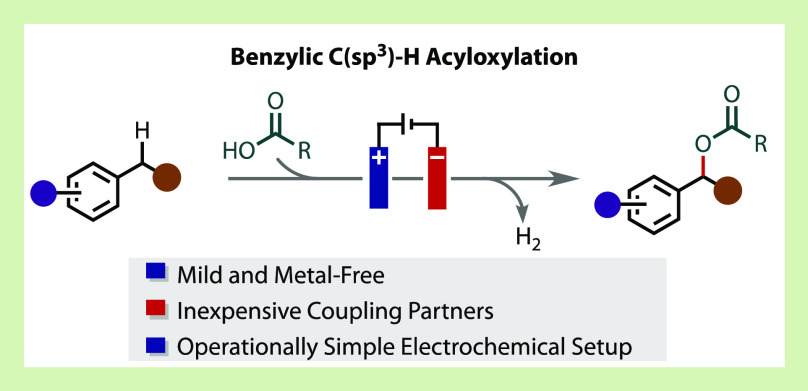

The development of sustainable C(sp^3^)–H functionalization methods is of great interest to the pharmaceutical and agrochemical industries. Anodic oxidation is an efficient means of producing benzylic cations that can undergo subsequent *in situ* nucleophilic attack to afford functionalized benzylic products. Herein, we demonstrate the suitability of carboxylic acids as nucleophiles to yield benzylic esters. This method employs a series of secondary benzylic substrates and functionalized carboxylic acids and is demonstrated on a gram scale in flow.

The selective functionalization of C(sp^3^)–H bonds is an efficient approach to the synthesis of complex molecules, as it negates the requirement to use prefunctionalized substrates.^1^ C–H functionalization is integral to the concept of “ideality”, as it introduces step-, time-, and waste-efficiency benefits into the synthesis of organic molecules. In particular, the diversification of benzylic positions through C–H functionalization is of great value in the context of pharmaceutical and agrochemical development, given the high propensity of this site to undergo enzymatic oxidation.^2−4^

A resurgence of interest in electrochemical synthesis has significantly expanded the organic chemist’s toolkit in recent years,^5,6^ largely due to the reactivity and sustainability benefits it brings to redox reactions.^7^ Indeed, electrochemical oxidation is an efficient means of generating benzylic cations from unfunctionalized benzylic C(sp^3^)–H bonds, via sequential electron-transfer, proton-transfer, and electron-transfer steps (ET/PT/ET). These reactive intermediates can be trapped by nucleophiles to afford functionalized benzylic products. Recently, amines, alcohols, isothiocyanates, and electron-rich aromatic rings have been reported as appropriate nucleophilic partners for electrochemically generated benzylic cations (Figure 1A).^8−14^

**Figure 1:**
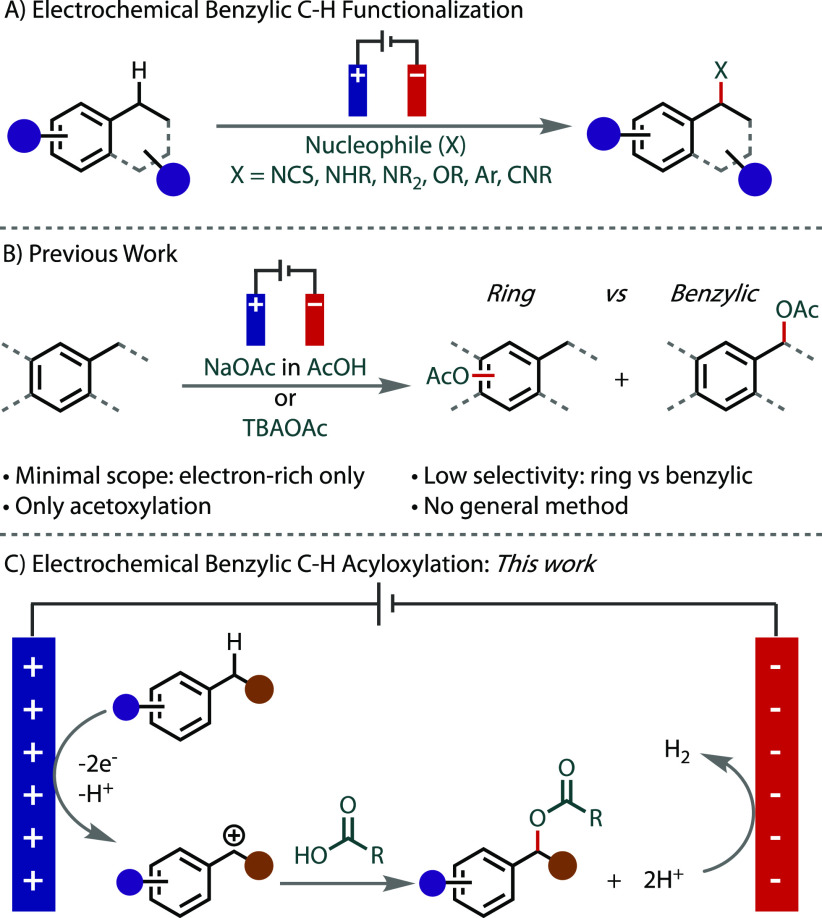
(A) Electrochemical benzylic functionalization. (B) Previous work in electrochemical benzylic C–H acetoxylation. (C) Electrochemical acyloxylation with exclusive substrate oxidation and trapping with a carboxylic acid derivative.

This strategy, however, is underexplored for coupling with carboxylic acids to form benzylic ester products, which are found in a myriad of important bioactive and high-value molecules.^15−20^ Although not highly nucleophilic, a carboxylic acid or carboxylate should serve as a competent nucleophile to quench highly reactive cations to afford a benzylic ester (Figure 1B), and they are functionally diverse, readily available, and inexpensive building blocks. Previous attempts using this approach have provided only limited progress. Early studies by Eberson showed that the electrolysis of benzyl-containing substrates (toluene, ethylbenzene, and mesitylene) in acetic acid and sodium acetate produced mixtures of ring and benzyl acetate products, with poor selectivity.^21,22^ The more electron-rich methylanisole was later found to give a higher yield under similar conditions,^23^ and TBAOAc was shown to be a suitable acetate source for the acetoxylation of 4-phenylethylbenzene.^10^ These reports highlight the need for the development of a simple and general method that directs reactivity to the benzylic position and prevents unwanted ring functionalization. In addition to expanding the scope of suitable benzylic compounds, the incorporation of carboxylic acids beyond acetic acid would be of great value to the synthesis of benzylic esters.

Unlike previous reports, our simple hypothesis was to find conditions that completely avoided oxidation of the carboxylic acid/carboxylate component. This was because under electrochemical oxidation, carboxylates/carboxylic acids give carboxyl radicals, which, following decarboxylation, are used for Kolbe/Kolbe-type coupling reactions.^24,25^ We hypothesized that the acetoxyl radicals generated may add to the aromatic ring, leading to the ring acetoxylation product.^26,27^ Hence, through the careful control of the carboxylic acid/carboxylate ratio, concentration, and electrode material,^28^ we envisioned that we could direct substrate oxidation and proton reduction on the anode and cathode, respectively. Thus, herein we report electrochemical conditions for the acyloxylation of benzylic C(sp^3^)–H bonds (Figure 1C). Initially, we exploited the availability and low cost of acetic acid as a carboxylic acid source to afford secondary benzylic acetates. These mild and metal-free conditions were then adapted to allow for a lower carboxylic acid loading, facilitating the use of higher-value coupling partners, which resulted in a series of benzylic esters with varied and useful functionality.

Optimization studies were initiated with a series of CV experiments. Biphenyl **1a** was selected as an appropriate model substrate, due to the high prevalence of biaryl scaffolds in biologically relevant molecules,^29^ with the fluorine handle allowing facile reaction monitoring by ^19^F NMR. Acetic acid was initially selected as the carboxylic acid coupling partner, due to its low cost and high availability. CV experiments indicated that benzylic substrate **1a** underwent anodic oxidation preferentially to acetic acid only in the complete absence of acetate ions (Figure S1). This insight into directing the desired pathway over the single-electron oxidation of acetate^25^ proved to be pivotal to our optimization studies. All electrolyses were undertaken on commercially available and standardized electrochemistry equipment.^30,31^ Subjecting **1a** to the NaOAc electrolyte in AcOH, as employed by Eberson, resulted in an only moderate yield of the product (Table 1, entry 1).^22^ Hence, we switched to acetic acid alone; with judicious choices of electrode material, **1a** concentration, supporting electrolyte, and solvent, ring acetoxylation could be completely avoided, leading to optimized conditions for the formation of **2a** (entry 2). Decreasing the amount of acetic acid to 10 equiv still led to the product without any ring acetoxylation, albeit with a reduced yield (entry 3). Switching from DCM to acetonitrile (entry 4), increasing the substrate concentration (entry 5), and changing the supporting electrolyte (entry 6) were all found to marginally decrease the yield of **2a**. Similarly, decreasing the applied current from 10 mA (entry 2) to 5 mA (entry 7) and using a commercially available divided cell, equipped with a glass frit membrane (entry 8), decreased the yield. Platinum cathodes have a low overpotential for proton reduction^28^ but in this transformation displayed reduced performance compared to that of a graphite cathode (entry 9 vs entry 2). Finally, the reaction was found to proceed smoothly in air with only a minor decrease in yield (entry 10).

**Table 1:**
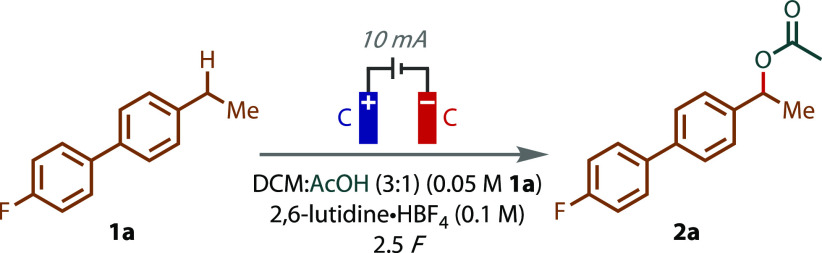
Selected Optimization Data for **2a** from **1a**^a^

entry	deviation	yield of **2a** (%)^b^
1	0.5 M NaOAc in AcOH	45
2	none	79
3	10 equiv of AcOH	48
4	3:1 MeCN/AcOH	69
5	0.1 M **1a**	72
6	TBAPF_6_ instead of 2,6-lutidine·HBF_4_	73
7	5 mA	68
8^c^	divided cell	18
9	Pt cathode	47
10	in air	72

a Reactions performed on a 0.2 mmol scale.

b ^19^F NMR yields based on the 1-fluoronaphthalene standard.

c Using an IKA Electrasyn Pro-Divide divided cell.

A series of substrates bearing secondary benzylic C(sp^3^)–H bonds were subjected to the reaction conditions to understand the generality of this electrochemical C–H functionalization reaction (Figure 2). Complete conversion of model substrate **1a** after 3 F afforded an 83% isolated yield of **2a**. Biphenyl **2b** and unsubstituted ethylbenzene **2c** were generated in slightly decreased yields. The inclusion of the sterically bulky and electron-donating *tert*-butyl group in the *meta* position resulted in a very good yield of **2d**. Substitution with a *p*-bromo group and increasing the alkyl chain length led to moderate yields of **2e** and **2f**, but with no other alkyl C–H bond functionalization observed. Strongly electron-withdrawing substituents (e.g., NO_2_) failed to lead to the desired product (Figure S3). Diphenylmethane and tetrahydronaphthalene underwent smooth reaction to afford products **2g** and **2h**. Alkyl ester **2i** and alkynyl product **2j** were prepared in moderate yields, the latter of which was due to incomplete conversion (even with the extended charge passed). Ibuprofen ethyl ester was subjected to the reaction conditions, returning acetoxylated product **2k** as a mixture of diastereomers. Finally, synthetic musk Celestolide was also transformed into the corresponding acetate **2l** in an excellent yield of 83%.

**Figure 2:**
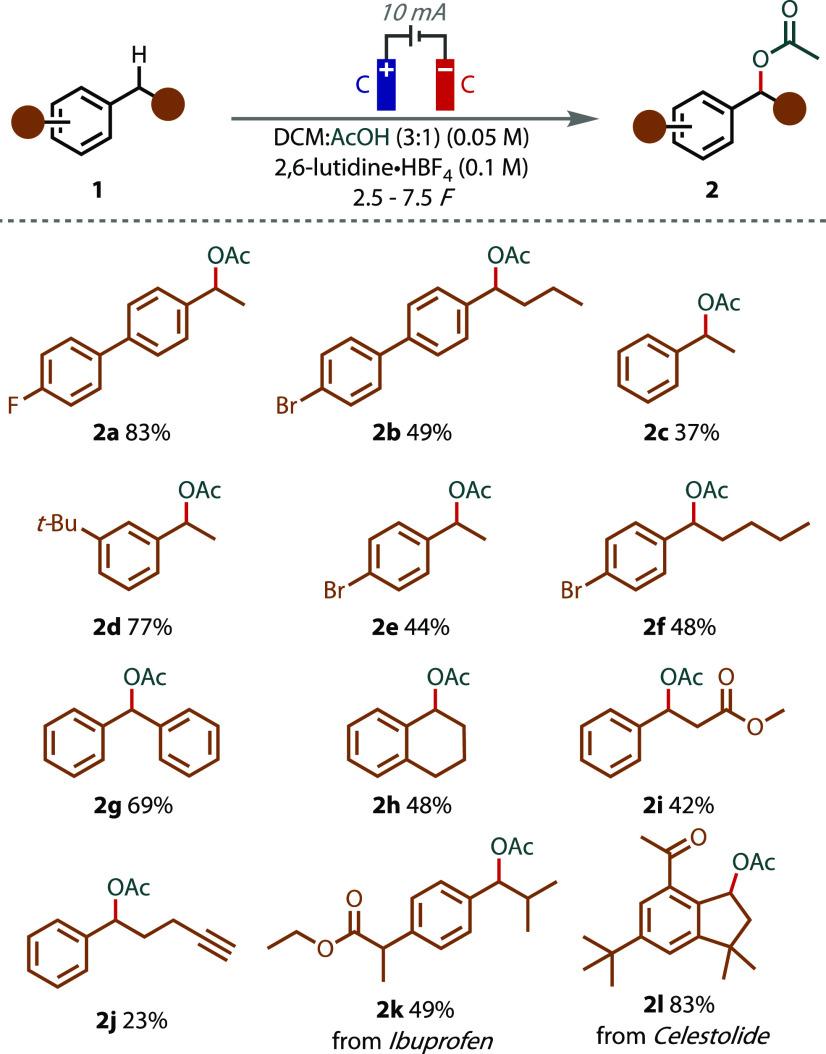
Electrochemical benzylic C–H acetoxylation substrate scope. All yields listed are isolated yields.

To further explore the generality of this C–O coupling reaction, a series of carboxylic acids bearing different functionalities were subjected to the conditions, with **1b** as the benzylic coupling partner (Figure 3). Optimization of the acetoxylation indicated the electrochemically generated benzylic cation could be trapped with non-solvent level acetic acid (Table 1, entry 3), which was a promising result for couplings with higher-value carboxylic acids. When **1b** was subjected to 10 equiv of benzoic acid as the coupling partner, benzyl ester **3a** was formed in an excellent isolated yield of 81%. With the use of 2 equiv, the reaction yield decreased to 42%. This observed variation reflects the sensitivity of the yield to the quantity of acid added, which is a decision dependent on the relative costs of the product and carboxylic acid. Considering many carboxylic acids are very inexpensive, the larger loadings contribute only a minor cost component of the total reaction mixture, while a more economic use of higher-value carboxylic acids is also possible. 4-Fluorobenzoic acid was poorly soluble in the reaction conditions, but a loading of just 3 equiv gave rise to 53% **3b**. Various alkyl-bearing carboxylic acids of varying size were tolerated in moderate to excellent yields. Carboxylic acids with straight chains, small rings, and bulky cyclic and open chains with quaternary centers were all well tolerated, giving esters **3c–3h** in moderate to excellent yields. Despite a 4-unit decrease in p*K*_a_ from acetic acid to trifluoroacetic acid corresponding to a decrease in nucleophilicity, a moderate yield of product **3j** was achieved, demonstrating this as a method for preparing high-value fluorinated molecules. Finally, carboxylic acids bearing unsaturated alkyl chains, acrylic and crotonic acid, gave rise to benzylic ester products **3k** and **3l**, respectively, in moderate to good yields.

**Figure 3:**
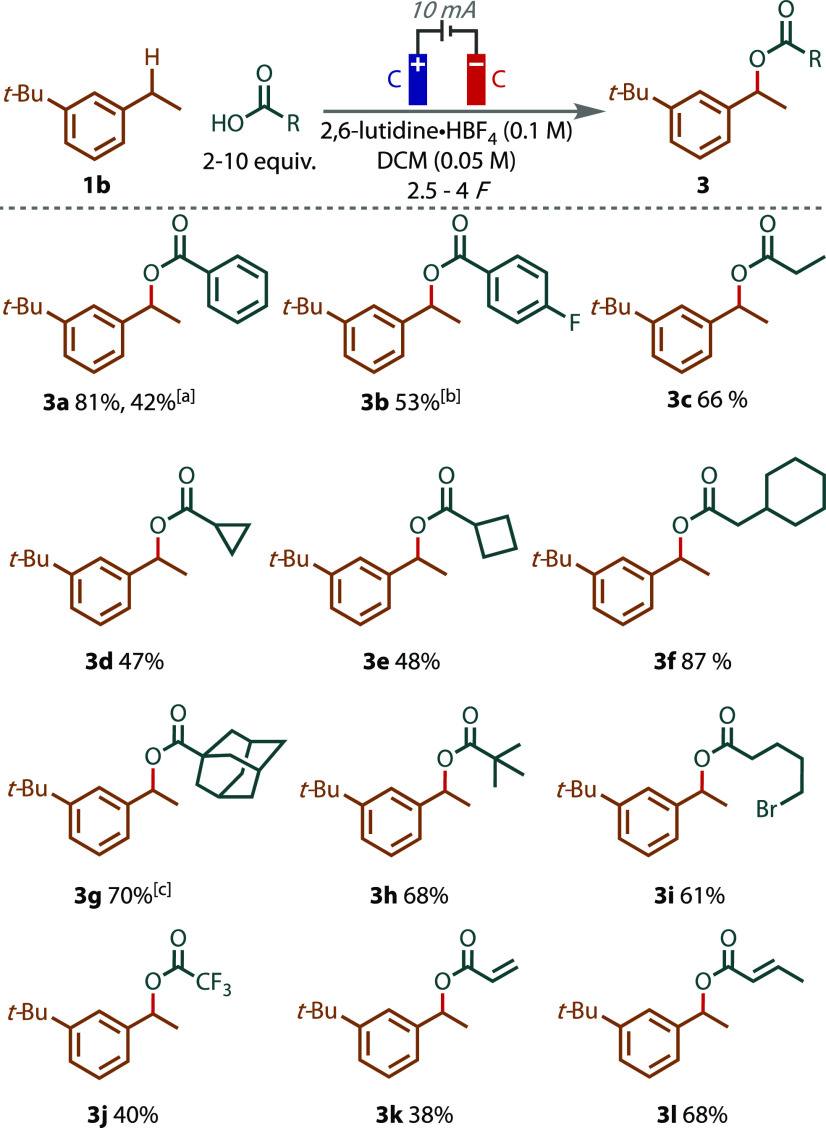
Isolated yields listed. Unless otherwise stated, 10 equiv of a carboxylic acid coupling partner employed. ^[a]^With 2 equiv of acid added. ^[b]^With 3 equiv of acid added. ^[c]^With 5 equiv of acid added.

The reaction conditions were translated from the commercially available batch setup into flow (Figure 4). Substrate **1g** was coupled with acetic acid using a commercially available flow cell and pump system. Approximately the same general conditions as batch were applied, such as graphite electrodes in a DCM/AcOH mixture. However, the electrolyte system was switched to effect better conductivity, the current density was reduced, and more charge was passed in a recirculating system to give an optimum yield of 51% and >1 g of **2g**.

**Figure 4:**
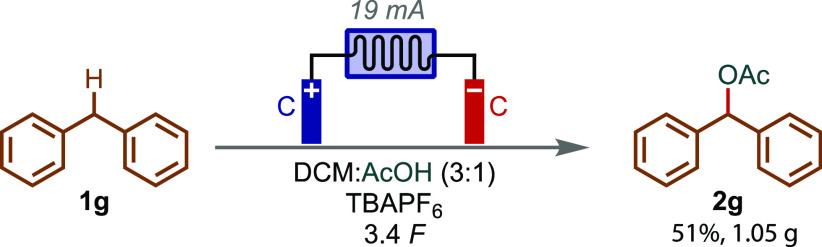
Flow electrolysis of **1g** and its reaction with acetic acid to produce **2g**.

In conclusion, we have reported a general method for the electrochemical acyloxylation of benzylic C(sp^3^)–H bonds. The protocol operates in a simple, commercially available electrochemical setup: an undivided cell under constant current electrolysis with inexpensive graphite working and counter electrodes. The reaction was found to achieve moderate to excellent yields for the acetoxylation of a series of secondary benzylic substrates. Furthermore, the scope of the reaction was extended by employing a range of carboxylic acids as coupling partners. The scalability of the reaction was demonstrated on a gram scale via the use of flow electrochemistry.

## Supporting Information Available

Experimental procedures and characterization data for known and new compounds (PDF)
