# Supplementary material for: Electrochemical Benzylic C(sp3)–H Acyloxylation
Source: Org Lett. 2022 Jul 13;24(28):5105–8. doi: 10.1021/acs.orglett.2c01930 (PMC9315976; doi:10.1021/acs.orglett.2c01930)
Supplement: Supplementary file 1 — ol2c01930_si_001.pdf [file ol2c01930_si_001.pdf]

Supporting Information

Electrochemical Benzylic C(sp^3)-H Acyloxylation

Alexander P. Atkins[†], Albert C. Rowett[†], David M. Heard[†], Joseph A. Tate[‡], Alastair J. J. Lennox[†]*

[†]University of Bristol, School of Chemistry, Cantock's Close, Bristol, BS8 1TS, United Kingdom

[‡]Syngenta, Jealott's Hill International Research Centre, Bracknell, RG42 6EY, United Kingdom

*Corresponding Author; Alastair J. J. Lennox, a.lennox@bristol.ac.uk

General Experimental Details 3

Cyclic Voltammograms 4

Substrate Preparation and Characterisation 5

General Reaction Procedures 9

General Procedure 1: Electrochemical Benzylic Acetoxylation *9*

Acetoxylation Products Characterisation *10*

Acetoxylation Failed or Low Yielding Substrates *16*

General Procedure 2: Electrochemical Benzylic Acyloxylation *17*

Acyloxylation Product Characterisation *18*

Acyloxylation Failed or Low Yielding Carboxylic Acids *24*

Electrochemical Flow Procedure and Optimisation 25

Electrochemical Flow Optimisation *25*

Recirculating Flow Procedure *26*

Electrochemical Setup 27

Compounds Spectra 30

Acetoxylation Product NMR Spectra *30*

Esterification Products NMR Spectra *43*

References 57

General Experimental Details

Air-sensitive procedures were carried out using Schlenk-line techniques under an atmosphere of N_2. Glassware was dried in a 180 °C oven before use. Chemicals were purchased from Sigma Aldrich, Acros, Fluorochem, TCI, Fisher Scientific, Alfa Aesar or Lancaster Chemicals and used as received unless otherwise stated. Additions of <200 µL were made with Gilson Pipetman pipettes. Anhydrous solvents were collected via the Anhydrous Engineering double alumina drying system located at the University of Bristol. Technical grade solvents were used for column chromatography. Column chromatography was performed using silica gel (230-400 mesh, 60 Å pore size) or via a Biotage Selekt with Sfär Silica D Duo capsules. TLC analysis was performed using SiO_2 coated aluminium plates visualized by UV fluorescence, iodine vapours, potassium permanganate, or phosphomolybdic acid stains.

NMR samples were submitted in $CDCl_3$ or MeCN-D_3 (Sigma Aldrich) and spectra were recorded on Bruker Nano 400, Jeol ECS 300, Jeol ECS 400, Jeol ECZ 400, Varian 500 or Bruker Advance III HD 500 Cryo spectrometers. Chemical shifts are reported in parts per million (ppm) and referenced to residual solvent. Coupling constants (*J*) are quoted in Hz. Multiplicities are reported as s (singlet), d (doublet), t (triplet), q (quartet), hept (heptet) and m (multiplet). NMR shifts for novel compounds have been assigned using the appropriate 2D NMR experiments, such as COSY, HSQC and HMBC.

IR analysis was performed on a PerkinElmer Spectrum 100 FTIR with an ATR accessory and frequencies reported in wavenumbers (cm^{-1}).

High Resolution Mass Spectrometry was recorded on QExactive (GC-Orbitrap), Orbitrap Elite (LC-Orbitrap) or Synapt G2S (IMS-Q-TOF) instruments using electron ionisation (EI) and nanospray techniques. Samples were submitted in DCM, MeCN or $CHCl_3$, or neat.

Electrolysis was performed using an IKA ElectraSyn 2.0 with ElectraSyn electrodes, lids and reaction vials. Graphite electrodes were sonicated in a 50/50 mixture of EtOH/Acetone and dried using a heat gun before use. If material had deposited onto the electrode surface a fine sandpaper was used to remove it before washing. CV analysis was performed using a Palmsens Multisens 4 with a glassy carbon working electrode, platinum wire counter electrode and a 0.1 M Ag/$AgNO_3$ reference electrode. All electrodes were polished and the reaction mixture was stirred and degassed by a stream of N_2 for approximately 90 seconds before each CV.

Flow electrolysis was carried out in an ElectroCell Micro Flow Cell using the 2 mm thick, 10 cm^2 electrode area PTFE flow frame and PTFE turbulence mesh, expanded PTFE gaskets, and two custom-made, impervious graphite, plate electrodes. Reaction mixtures were pumped with either a syringe pump or a Vapourtec E-Series Flow Chemistry System peristaltic pump, using a variety of rubber and plastic tubing and adapters. A Tenma DC power supply was used as a power source.

Cyclic Voltammograms

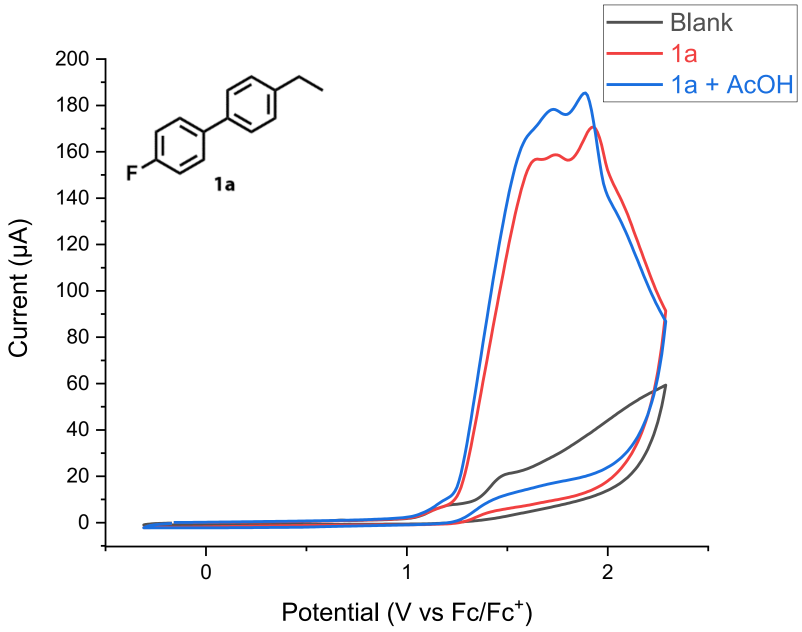

Figure S1. Cyclic voltammogram of substrate **1a** with and without acetic acid.

1a (5mM) and AcOH (5 mM) in DCM (anhydrous and degassed by stream of N_2) with $TBAPF_6$ (0.1 M) supporting electrolyte. Glassy carbon disc working electrode, platinum wire counter electrode and $Ag/AgNO_3$ reference electrode. 0.1 Vs^{-1} scan rate. Referenced vs Fc/Fc^+.

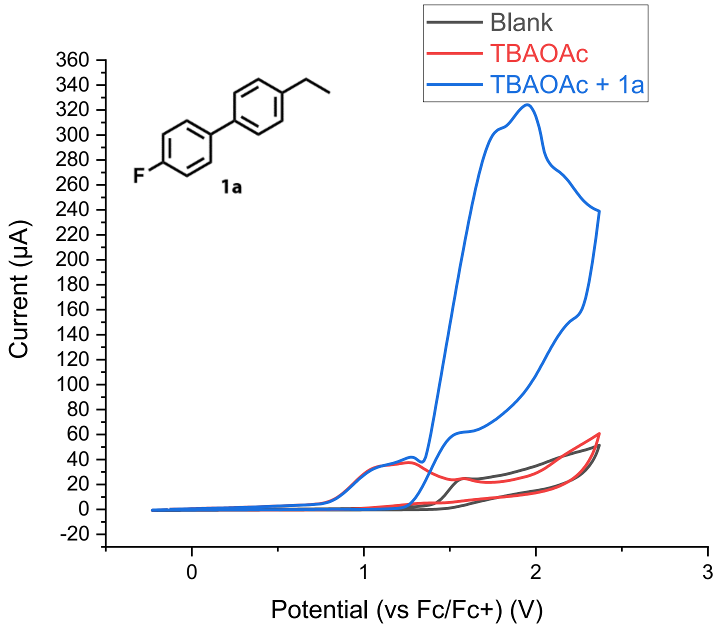

Figure S2. Cyclic voltammogram of acetate ion with and without **1a**.

TBAOAc (5mM) and **1a** (5mM) in DCM (anhydrous and degassed by stream of N_2) with $TBAPF_6$ (0.1 M) supporting electrolyte. Glassy carbon disc working electrode, platinum wire counter electrode and $Ag/AgNO_3$ reference electrode. 0.1 Vs^{-1} scan rate. Referenced vs Fc/Fc^+.

Substrate Preparation and Characterisation

4-Ethyl-4′-fluoro-1,1′-biphenyl (**1a**)

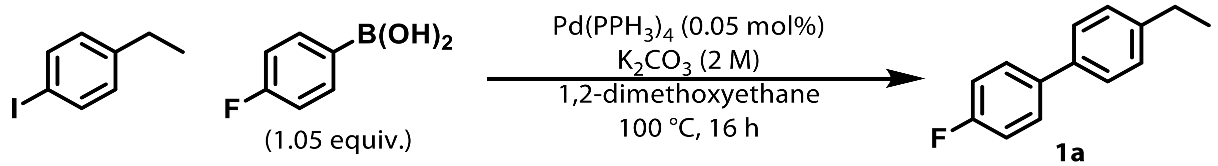

The title compound was prepared from an adapted known procedure.[1] To a round-bottom flask equipped with a magnetic stirrer bar was added 4-fluorophenylboronic acid (10.5 mmol, 1.05 equiv., 1470mg), 4-iodoethylbenzene (10.0 mmol, 1.0 equiv., 2320 mg), and $Pd(PPh_3)_4$ (0.05 mmol, 0.005 equiv., 57.8 mg). To this was added 2 M K_2CO_3 (10.0 mL) and 1,2-dimethoxyethane (25.0 mL) and the reaction mixture placed in a pre-heated heating mantle set to 100 °C. The reaction mixture was refluxed for 16 h and then allowed to cool to room temperature. Then was added sat. aq. NH_4Cl. The resulting mixture was partitioned in a separating funnel and the organics extracted into DCM (3 x 50 mL). The organic extracts were combined, dried with $MgSO_4$, filtered, and dried under reduced pressure to afford crude product, which was purified by column chromatography (100% pentane) to afford 1670 mg (83%) of **1a** as a white solid.

^{1}H NMR (400 MHz, $CDCl_3$) δ 7.57 – 7.50 (m, 2H), 7.47 (d, *J* = 8.1 Hz, 2H), 7.31 – 7.25 (m, 2H), 7.16 – 7.07 (m, 2H), 2.69 (q, *J* = 7.6 Hz, 2H), 1.28 (t, *J* = 7.6 Hz, 3H).
^{19}F NMR (377 MHz, $CDCl_3$) δ -116.2 (tt, *J* = 8.7, 5.4 Hz).
^{13}C NMR (101 MHz, $CDCl_3$) δ 162.5 (d, *J* = 245.8 Hz), 143.6, 137.8, 137.5 (d, *J* = 3.2 Hz), 128.6 (d, *J* = 8.0 Hz), 128.5, 127.1, 115.7 (d, *J* = 21.4 Hz), 28.6, 15.7.
Data are in agreement with the literature.[2]

Ibuprofen ethyl ester (**1k**)

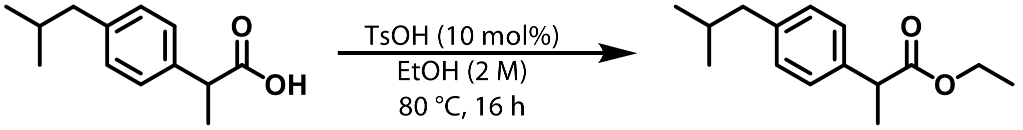

The title compound was prepared according to a known procedure.[3] To a 25-mL round-bottom flask equipped with a magnetic stirrer bar and under a stream of N_2 was added *p*-toluenesulfonic acid monohydrate (0.5 mmol, 0.1 equiv., 95.1 mg) and ibuprofen (5.0 mmol, 1 equiv., 1030 mg). To this was added anhydrous EtOH (5 mL) and the reaction mixture heated at reflux for 16 hours. The reaction mixture was then cooled to room temperature and the solvent removed under reduced pressure. The remaining residue was dissolved in DCM and washed with 2 M NaOH (1 x 20 mL). The organic layer was dried with $MgSO_4$, filtered and dried under vacuum to afford 1107 mg (95%) of **1k** as a colourless oil.

^{1}H NMR (400 MHz, $CDCl_3$) δ 7.22 – 7.18 (m, 2H), 7.11 – 7.07 (m, 2H), 4.20 – 4.04 (m, 2H), 3.68 (q, *J* = 7.2 Hz, 1H), 2.45 (d, *J* = 7.2 Hz, 2H), 1.85 (dt, *J* = 13.4, 6.7 Hz, 1H), 1.48 (d, *J* = 7.2 Hz, 3H), 1.21 (t, *J* = 7.1 Hz, 3H), 0.90 (d, *J* = 6.6 Hz, 6H).
^{13}C NMR (101 MHz, $CDCl_3$) δ 175.0, 140.6, 138.0, 129.4, 127.3, 60.8, 45.3, 45.2, 30.3, 22.5, 18.7, 14.3.
Data are in agreement with the literature.[4]

Preparation of 2,6-Lutidine•HBF_4

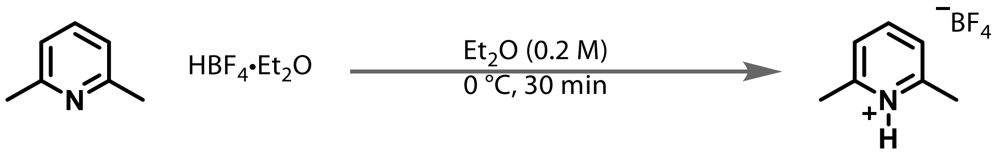

To a 250-mL round-bottom flask equipped with a magnetic stirrer bar was added HBF_4•Et_2O (40.0 mmol, 1.0 equiv., 5.40 mL) and Et_2O (80 mL), and the reaction vessel was cooled and stirred at 0 °C. 2,6-Lutidine (40.0 mmol, 1.0 equiv., 4.62 mL) was added dropwise to the cooled reaction vessel. The reaction mixture was allowed to stir at 0 °C until no more precipitate formed (approximately 30 mins), after which the precipitate was filtered and underwent several cold Et_2O washes. The BF_4 salt was milled with a pestle and mortar and dried under reduced pressure overnight to afford 2,6-lutidine•HBF_4 (4.3 g, 56%) as a pink solid.

^{1}H NMR (400 MHz, $CDCl_3$) δ 8.23 (t, *J* = 7.9 Hz, 1H), 7.56 (d, *J* = 7.9 Hz, 2H), 2.86 (s, 6H).
^{13}C NMR (101 MHz, $CDCl_3$) δ 154.3, 146.1, 124.9, 19.9.
^{19}F NMR (377 MHz, $CDCl_3$) δ -149.3 (s), -149.4 (s).

2,6-Lutidine•HBF_4

^{1}H NMR (400 MHz, $CDCl_3$)

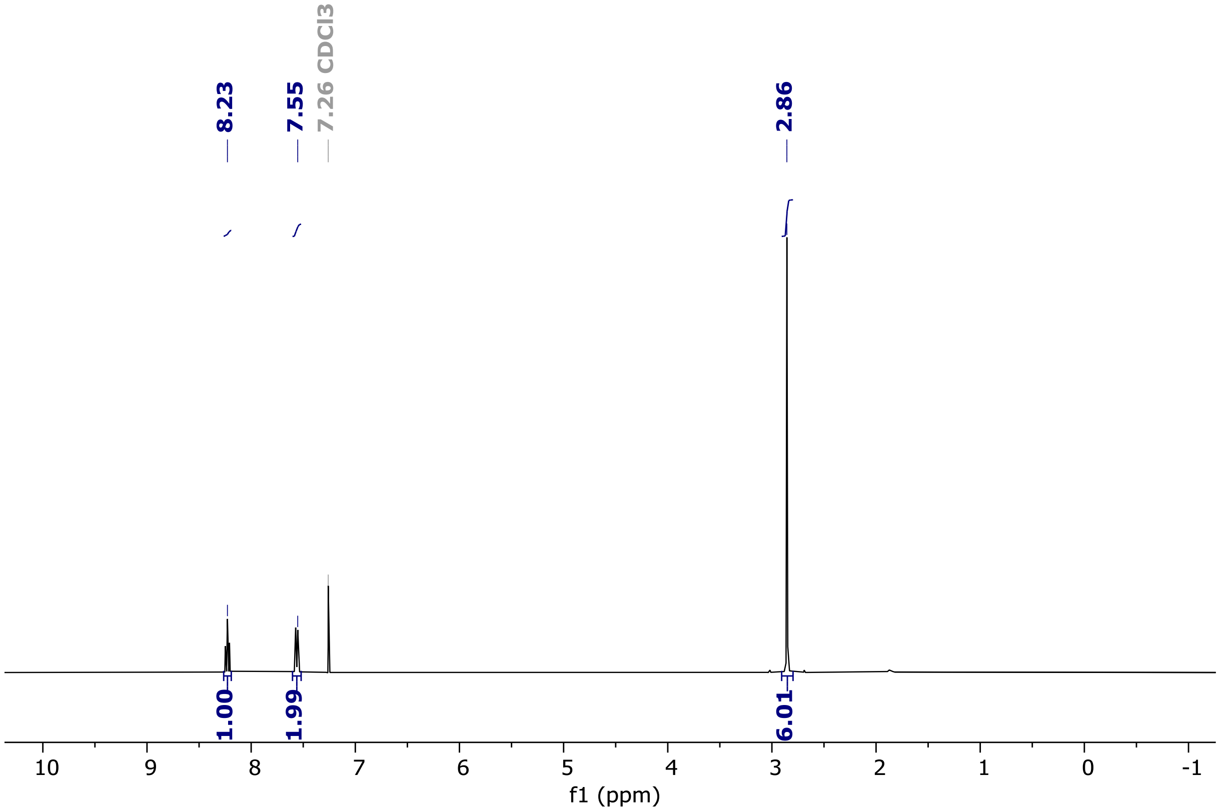

^{13}C NMR (101 MHz, $CDCl_3$)

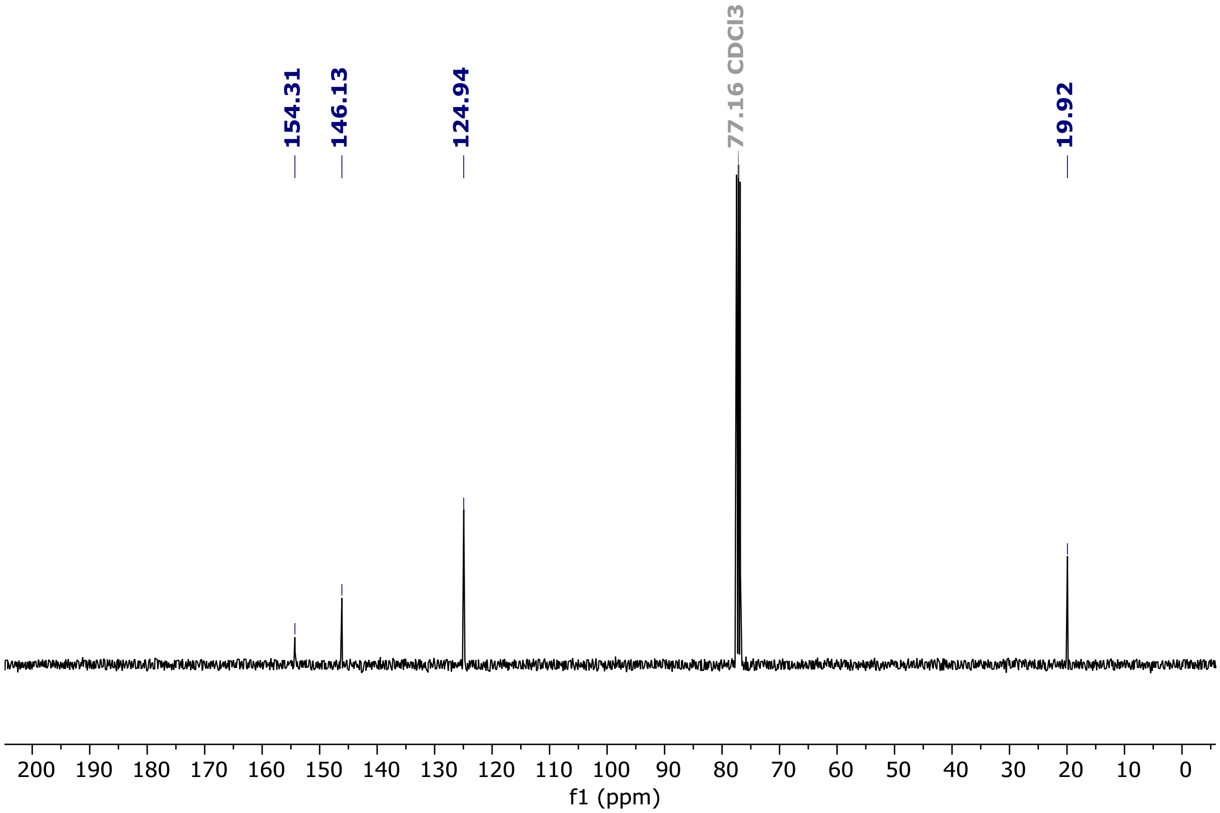

^{19}F NMR (377 MHz, $CDCl_3$)

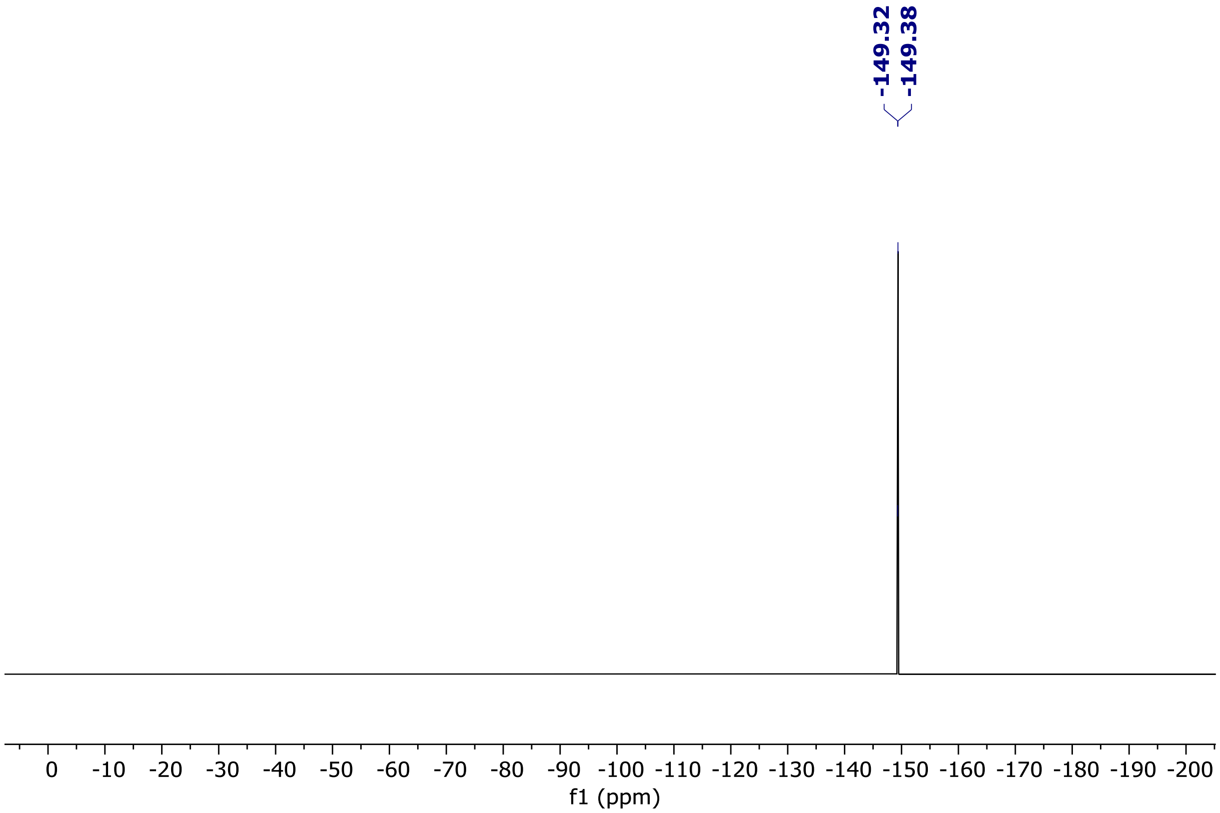

2,6-Lutidine•HBF_4 ^1H NMR Compared to 2,6-Lutidine

^{1}H NMR (400 MHz, $CDCl_3$)

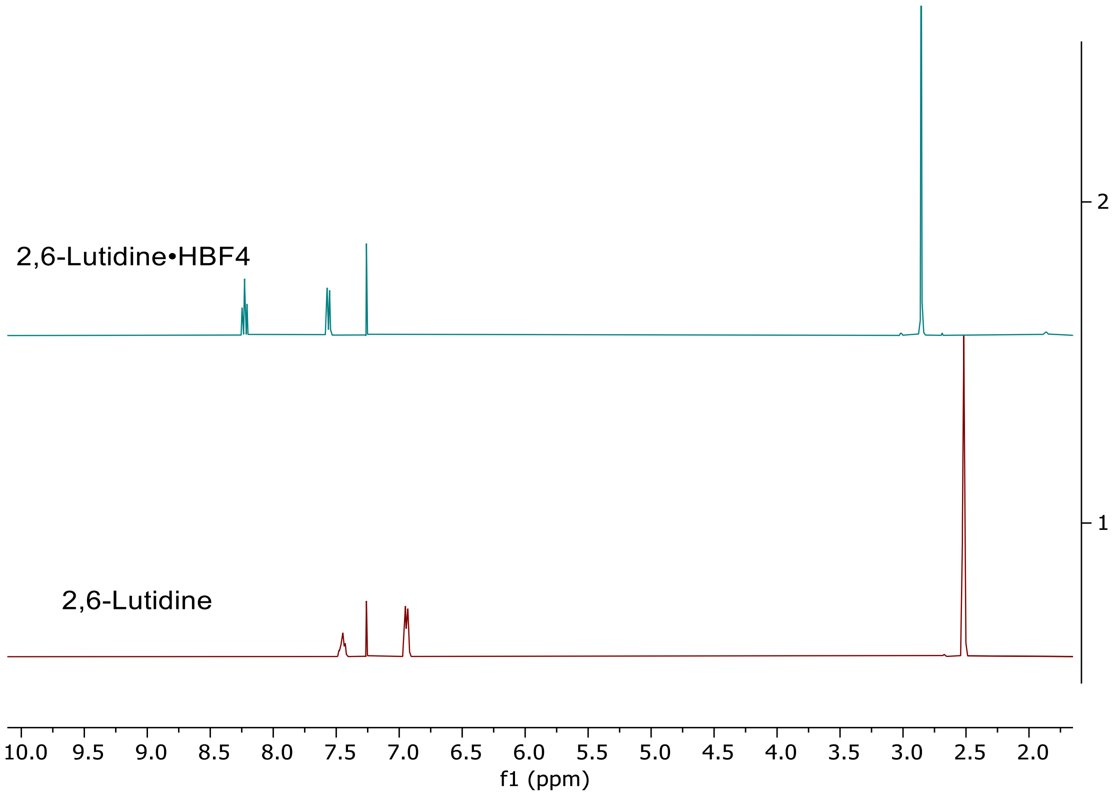

General Reaction Procedures

General Procedure 1: Electrochemical Benzylic Acetoxylation

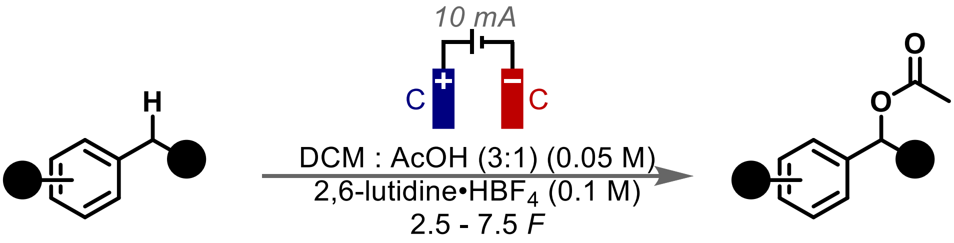

To a 5-mL oven-dried ElectraSyn vial equipped with a magnetic stirrer bar was added substrate (0.2 mmol, 1 equiv.) and 2,6-lutidine•HBF_4 (0.40 mmol, 2.0 equiv., 77.8 mg). The ElectraSyn vial threads were wrapped in PTFE tape and the vial sealed with a lid bearing a suba-seal and graphite working and counter electrodes (**Figure S5**). The vial was then evacuated and back-filled with N_2 three times. Then were added DCM (3.0 mL) and glacial acetic acid (1.0 mL) and the reaction allowed to stir for 5 mins. An N_2 balloon was placed through the suba-seal and the reaction mixture subjected to electrolysis on the ElectraSyn 2.0 (10 mA constant current for 2.5 *F*) after which the reaction progress was monitored by TLC (ethyl acetate in pentane) and the reaction allowed to continue in 0.5 *F* steps until complete consumption of the starting material was observed. Upon completion, the reaction mixture was added to a separating funnel and sat. aq. $NaHCO_3$ (50 mL) and water (20 mL) were added. The electrodes were rinsed with DCM and the washings added to the separating funnel. The organic layer was extracted three times with DCM (10 mL), dried with $MgSO_4$, filtered, and solvent was removed under reduced pressure. The product was isolated by column chromatography (ethyl acetate in pentane).

Note: For volatile substrates the reaction mixture was not evacuated under vacuum but was purged with N_2 for 5 min before addition of DCM and acetic acid.

Acetoxylation Products Characterisation

1-(4-Fluoro-1[1,1′-biphenyl]-4-yl)ethyl acetate (**2a**)

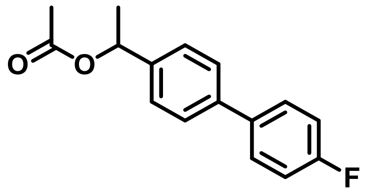

Title compound **2a** was prepared from 4-ethyl-4′-fluoro-1,1′-biphenyl (**1a**) via general procedure 1 and isolated by column chromatography (5% ethyl acetate in pentane) to afford an off-white solid (43 mg, 83%) after 3 *h*.

^{1}H NMR (400 MHz, $CDCl_3$) δ = 7.56 – 7.49 (m, 4H), 7.44 – 7.40 (m, 2H), 7.16 – 7.08 (m, 2H), 5.92 (q, *J*= 6.6, 1H), 2.09 (s, 3H), 1.57 (d, *J*= 6.6, 3H).
^{13}C NMR (101 MHz, $CDCl_3$) δ = 170.5, 162.7 (d, *J*=246.4), 140.9, 140.0, 137.0 (d, *J*=3.1), 128.8 (d, *J* =8.1), 127.3, 126.8, 115.8 (d, *J*=21.4), 72.2, 22.3, 21.5.
^{19}F NMR (377 MHz, $CDCl_3$) δ -115.5 (tt, *J*= 8.6, 5.3 Hz).
HRMS (EI+) calc: [M^+] ($C_{16}H_{15}O_2F$) 258.1051, measured 258.1053, 0.77 ppm error.
IR (neat) ν_{max}/cm^{-1}: 2987, 2901, 1733, 1601, 1497, 1372, 1238, 1066, 1027.

*1-(4′-Bromo-[1,1′-biphenyl]-4-yl)butyl acetate (**2b**)*

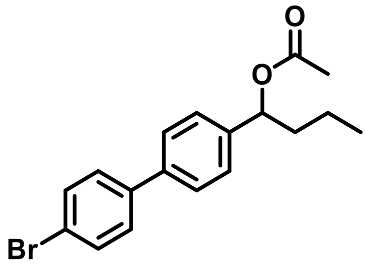

Title compound **2b** was prepared from 4-bromo-4′-butyl-1,1′-biphenyl via general procedure 1 and isolated by column chromatography (5% diethyl ether in pentane) to afford a colourless solid (34 mg, 49%) after 7.5 *h*.

^{1}H NMR (400 MHz, $CDCl_3$) δ 7.58 – 7.50 (m, 4H), 7.46 – 7.38 (m, 4H), 5.77 (dd, *J*= 7.7, 6.2 Hz, 1H), 2.08 (s, 3H), 1.98 – 1.87 (m, 1H), 1.83 – 1.71 (m, 1H), 1.46 – 1.23 (m, 2H), 0.94 (t, *J*= 7.4 Hz, 3H).
^{13}C NMR (101 MHz, $CDCl_3$) δ 170.6, 140.5, 139.8, 139.7, 132.0, 128.8, 127.2, 127.1, 121.7, 75.8, 38.5, 21.4, 18.9, 13.9.
HRMS (EI^+) calc: [M^+] ($C_{18}H_{19}BrO_2$) 346.0563, measured 346.0561, 0.58 ppm error.
IR (neat) ν_{max}/cm^{-1}: 2959, 1734, 1483, 1371, 1230, 1023, 1001, 811.

1-Phenylethyl acetate (**2c**)

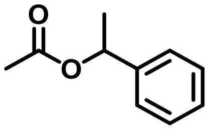

Title compound **2c** was prepared from ethylbenzene via general procedure 1 and isolated by column chromatography (5% ethyl acetate in pentane) to afford a colourless oil (12.1 mg, 37%) after 3 *F*.

^{1}H NMR (400 MHz, $CDCl_3$) δ 7.37 – 7.27 (m, 5H), 5.89 (q, *J* = 6.6 Hz, 1H), 2.07 (s, 3H), 1.54 (d, *J* = 6.6 Hz, 3H).
^{13}C NMR (101 MHz, $CDCl_3$) δ 170.5, 141.8, 128.6, 128.0, 126.2, 72.4, 22.3, 21.5.
Data are in agreement with the literature.[5]

*1-(3-(tert-butyl)phenyl)ethyl acetate (**2d**)*

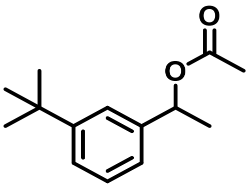

Title compound **2d** was prepared from 1-(tert-butyl)-3-ethylbenzene via general procedure 1 and isolated by column chromatography (2% ethyl acetate in pentane) to afford a colourless oil (34 mg, 77%) after 2.5 *F*.

^{1}H NMR (400 MHz, $CDCl_3$) δ 7.38 – 7.27 (m, 3H), 7.21 – 7.17 (dt, *J* = 7.3, 1.5 Hz, 1H), 5.90 (q, *J* = 6.6 Hz, 1H), 2.08 (s, 3H), 1.55 (d, *J* = 6.6 Hz, 3H), 1.33 (s, 9H).
^{13}C NMR (101 MHz, $CDCl_3$) δ 170.5, 151.5, 141.4, 128.3, 125.1, 123.3, 123.3, 72.8, 34.9, 31.5, 22.4, 21.5.
HRMS (EI+) calc: [M+] ($C_{14}H_{20}O_2$) 220.1458, measured 220.1456, 0.91 ppm error.
IR (neat) ν_{max}/cm^{-1}: 2964, 1734, 1369, 1239, 1205, 1066, 707.

1-(4-Bromophenyl)ethyl acetate (**2e**)

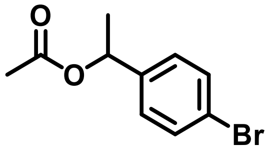

Title compound **2e** was prepared from 1-bromo-4-ethylbenzene via general procedure 1 and isolated by column chromatography (5% ethyl acetate in pentane) to afford a colourless oil (21.3 mg, 44%) after 3 *F*.

^{1}H NMR (400 MHz, $CDCl_3$) δ 7.49 – 7.45 (m, 2H), 7.25 – 7.20 (m, 2H), 5.82 (q, *J* = 6.6 Hz, 1H), 2.07 (s, 3H), 1.51 (d, *J* = 6.6 Hz, 3H).
^{13}C NMR (101 MHz, $CDCl_3$) δ 170.3, 140.9, 131.8, 128.0, 121.9, 71.8, 22.3, 21.4.
Data are in agreement with the literature.[6]

*1-(4-Bromophenyl)pentyl acetate (**2f**)*

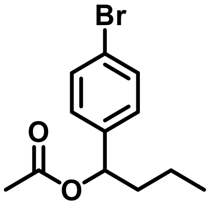

Title compound **2f** was prepared from 1-bromo-4-pentylbenzene via general procedure 1 and isolated by column chromatography (5% ethyl acetate in pentane) to afford a colourless oil (27.8 mg, 48%) after 2.5 *F*.

^{1}H NMR (400 MHz, $CDCl_3$) δ 7.50 – 7.42 (m, 2H), 7.24 – 7.16 (m, 2H), 5.65 (dd, *J* = 7.5, 6.5 Hz, 1H), 2.06 (s, 3H), 1.88 (dddd, *J* = 13.5, 10.5, 7.5, 5.0 Hz, 1H), 1.72 (dddd, *J* = 13.5, 10.0, 6.5, 5.5 Hz, 1H), 1.36 – 1.25 (m, 3H), 1.18 (m, 1H), 0.87 (t, *J* = 7.0 Hz, 3H).
^{13}C NMR (101 MHz, $CDCl_3$) δ 170.8, 140.4, 132.0, 128.7, 122.1, 76.0, 36.3, 28.0, 22.8, 21.7, 14.4.
HRMS (EI+) calc: [M^+] ($C_{13}H_{17}BrO_2$) 284.0406, measured 284.0406, 0.00 ppm error.
IR (neat) ν_{max}/cm^{-1}: 2956, 2932, 1735, 1488, 1231, 1010, 820, 546.

Benzhydryl acetate (**2g**)

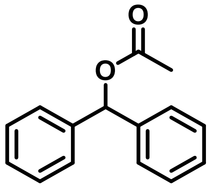

Title compound **2g** was prepared from diphenylmethane via general procedure 1 and isolated by column chromatography (2% ethyl acetate in pentane) to afford a colourless oil (31 mg, 69%) after 3 *F*.

^{1}H NMR (400 MHz, $CDCl_3$) δ 7.38 – 7.27 (m, 10H), 6.89 (s, 1H), 2.17 (s, 3H).
^{13}C NMR (101 MHz, $CDCl_3$) δ 170.2, 140.4, 128.6, 128.0, 127.2, 77.0, 21.4.
Data are in agreement with the literature.[7]

1,2,3,4-Tetrahydronaphthalen-1-yl acetate (**2h**)

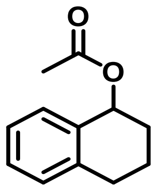

Title compound **2h** was prepared from 1,2,3,4-tetrahydronaphthalene via general procedure 1 and isolated by column chromatography (1% ethyl acetate in pentane) to afford a pale-yellow oil (18.3 mg, 48%) after 3 *F*.

^{1}H NMR (400 MHz, $CDCl_3$) δ 7.33 – 7.09 (m, 4H), 6.01 (t, *J* = 4.3 Hz, 1H), 2.95 – 2.68 (m, 2H), 2.09 (s, 3H), 2.04 – 1.91 (m, 3H), 1.90 – 1.77 (m, 1H).
^{13}C NMR (101 MHz, $CDCl_3$) δ = 170.9, 138.1, 134.7, 129.6, 129.2, 128.2, 126.2, 70.1, 29.2, 29.1, 21.6, 18.9.
Data are in agreement with the literature.[6]

Methyl 3-acetoxy-3-phenylpropanoate (**2i**)

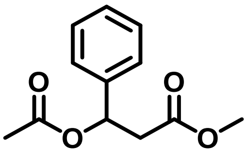

Title compound **2i** was prepared from methyl 3-phenylpropanoate via general procedure 1 and isolated by column chromatography (10% ethyl acetate in pentane) to afford a colourless oil (18.7 mg, 42%) after 7.5 *F*.

^{1}H NMR (400 MHz, $CDCl_3$) δ 7.39 – 7.29 (m, 5H), 6.17 (dd, *J* = 9.0, 5.0 Hz, 1H), 3.67 (s, 3H), 2.98 (dd, *J* = 16.0, 9.0 Hz, 1H), 2.76 (dd, *J* = 16.0, 5.0 Hz, 1H), 2.06 (s, 3H).
^{13}C NMR (101 MHz, $CDCl_3$) δ 170.4, 170.0, 139.4, 128.8, 128.5, 126.6, 72.2, 52.0, 41.4, 21.2.
Data are in agreement with the literature.[8]

*1-Phenylpent-4-yn-1-yl acetate (**2j**)*

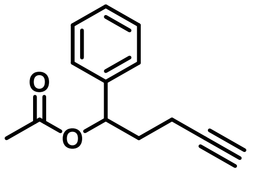

Title compound **2j** was prepared from pent-4-yn-1-ylbenzene via general procedure 1 and isolated by column chromatography (5% ethyl acetate in pentane) to afford a colourless oil (9.4 mg, 23%) after 7.5 *F*. Running for 2.5 *F* yielded 19%.

^{1}H NMR (400 MHz, $CDCl_3$) δ 7.40 – 7.27 (m, 5H), 5.85 (dd, *J* = 7.5, 6.0 Hz, 1H), 2.26 – 2.11 (m, 3H) 2.08 (s, 3H), 2.03 – 1.93 (m, 2H).
^{13}C NMR (101 MHz, $CDCl_3$) δ 170.3,140.1, 128.7, 128.2, 126.6, 83.1, 74.9, 69.2, 35.2, 21.4, 15.1.
HRMS (EI+) calc: $[M-H]^+$ ($C_{13}H_{13}O_2$) 201.0910, measured 201.0909, 0.50 ppm error.
IR (neat) v_{max}/cm^{-1}: 3676, 3289, 2989, 2972, 2900, 2118, 1738, 1394, 1241, 1046, 700, 640.

*Ethyl 2-(4-(1-acetoxy-2-methylpropyl)phenyl)propanoate (**2k**)*

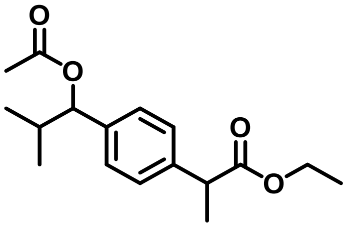

Title compound **2k** was prepared from ibuprofen ethyl ester (**1k**) via general procedure 1 and isolated by column chromatography (10% ethyl acetate in pentane) to afford a colourless oil (28.9 mg, 49%) after 3.5 *F*.

^{1}H NMR (400 MHz, $CDCl_3$) δ 7.29 – 7.18 (m, 4H), 5.45 (d, *J* = 7.5 Hz, 1H), 4.20 – 4.02 (m, 2H), 3.68 (q, *J* = 7.0 Hz, 1H), 2.13 – 2.00 (m, 4H), 1.47 (app. dd, *J* = 7.0, 1.0 Hz, 3H), 1.20 (app. td, *J* = 7.0, 0.5 Hz, 3H), 0.95 (d, *J* = 6.5 Hz, 3H), 0.78 (d, *J* =7.0 Hz, 3H).
^{13}C NMR (101 MHz, $CDCl_3$) δ 174.7*, 170.5, 140.1*, 138.6, 127.4, 127.3, 80.8*, 60.9, 45.3*, 33.6, 21.3, 18.8, 18.7*, 18.6, 14.2.
Peaks marked * are present as two peaks, as this compound is synthesised as a mixture of diastereomers.
HRMS (EI+) calc: $[M^+]$ ($C_{17}H_{24}O_4$) 292.1669, measured 292.1670, 0.34 ppm erorr.
IR (neat) v_{max}/cm^{-1}: 2976, 1727, 1372, 1243, 1020, 908, 732.

*7-Acetyl-5-(tert-butyl)-3,3-dimethyl-2,3-dihydro-1H-inden-1-yl acetate (**2l**)*

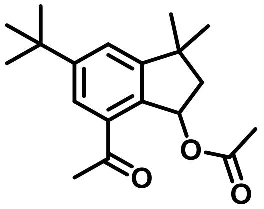

Title compound **2l** was prepared from celestolide via general procedure 1 and isolated by column chromatography (10% ethyl acetate in pentane) to afford a colourless solid (50.5 mg, 83%) after 2.5 *F*.

^{1}H NMR (400 MHz, $CDCl_3$) δ 7.74 (d, *J* = 1.5 Hz, 1H), 7.41 (d, *J* = 1.5 Hz, 1H), 6.52 (dd, *J* = 7.0, 2.5 Hz, 1H), 2.58 (s, 3H), 2.34 (dd, *J* = 14.5, 7.0 Hz, 1H), 2.06 – 1.98 (m, 4H), 1.37 (s, 9H), 1.34 (s, 3H) 1.32 (s, 3H).
^{13}C NMR (101 MHz, $CDCl_3$) δ 199.4, 170.7, 155.7, 153.5, 135.9, 134.7, 125.8, 123.5, 75.8, 48.5, 42.8, 35.1, 31.5, 31.2, 29.4, 28.4, 21.2.
HRMS (EI+) calc: $[M\text{-}COCH_3]^+$ ($C_{17}H_{23}O_2$) 259.1693, measured 259.1693, 0.00 ppm error.
IR (neat) ν_{max}/cm^{-1}: 2954, 1730, 1683, 1364, 1236, 908, 731.

Acetoxylation Failed or Low Yielding Substrates

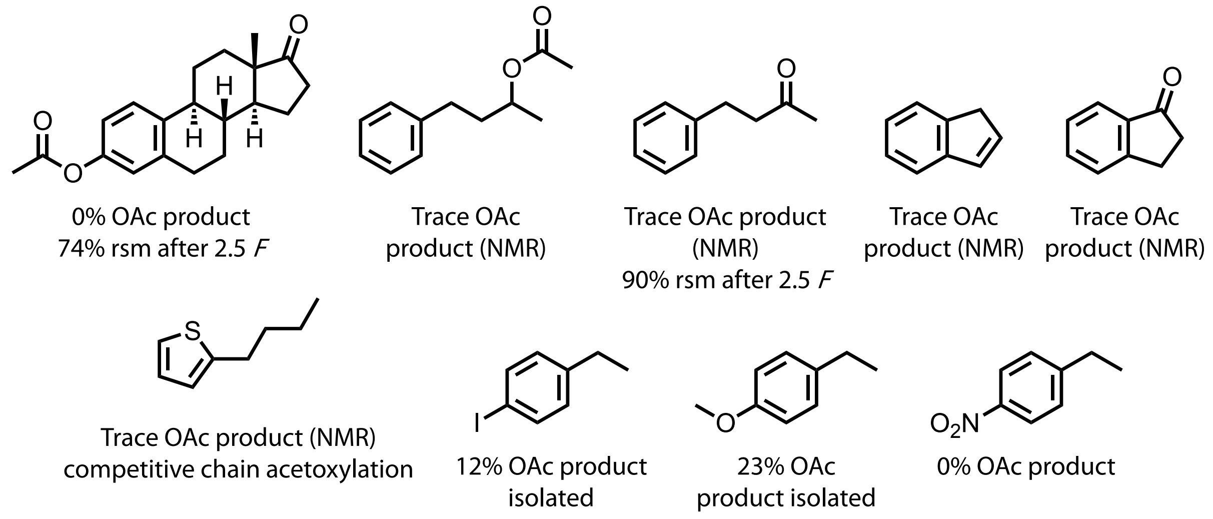

Figure S3. Failed or low yielding substrates.

General Procedure 2: Electrochemical Benzylic Acyloxylation

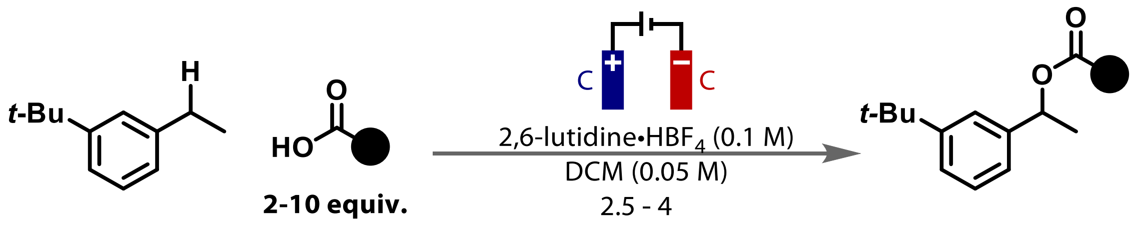

To a 5-mL oven-dried ElectraSyn vial equipped with a magnetic stirrer bar was added 1-tert-butyl-3-ethylbenzene (0.20 mmol, 1.0 equiv., 37.7 µL), acid (0.6-2.0 mmol, 3-10 equiv.) and 2,6-lutidine•HBF_4 (0.40 mmol, 2.0 equiv., 77.8 mg). The ElectraSyn vial threads were wrapped in PTFE tape and the vial sealed with a lid bearing graphite working and counter electrodes and a suba-seal (**Figure S5**). The vial was then purged by a stream of N_2 for 5 min. Then was added DCM (4.0 mL) and the reaction mixture allowed to stir for 5 min. A balloon filled with N_2 was placed through the suba-seal and the reaction mixture subjected to electrolysis on the ElectraSyn 2.0 (10 mA constant current for 2.5 *F*) after which the reaction progress was monitored by TLC (typically 2% ethyl acetate in pentane) and the reaction allowed to continue in 0.5 *F* steps until complete consumption of the starting material was observed. Upon completion, the reaction mixture was added to a separating funnel and sat. aq. $NaHCO_3$ (20 mL) and water (20 mL) were added. The electrodes were rinsed with DCM and the washings added to the separating funnel. The organic layer was extracted three times with DCM (10 mL), dried with $MgSO_4$, filtered, and solvent was removed under reduced pressure. Pure product was isolated by column chromatography (typically 0-4% gradient ethyl acetate in pentane).

Acyloxylation Product Characterisation

*1-(3-(tert-Butyl)phenyl)ethyl benzoate (**3a**)*

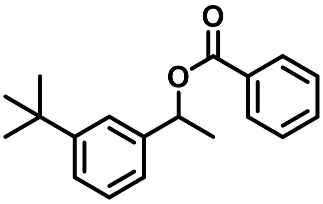

Title compound **3a** was prepared via general procedure 2 with 10 equivalents of benzoic acid and isolated by column chromatography (2% ethyl acetate in pentane) to afford a colourless oil (45.6 mg, 81%) after 2.5 *F*. With 2 equivalents of benzoic acid 23.8 mg (42%) was isolated.

^{1}H NMR (400 MHz, $CDCl_3$) δ 8.13 - 8.06 (m, 2H,), 7.56 (tt, *J* = 7.3, 1.4 Hz, 1H), 7.48 – 7.42 (m, 3H), 7.38 – 7.28 (m, 3H), 6.16 (q, *J* = 6.6 Hz, 1H), 1.69 (d, *J* = 6.6 Hz, 3H), 1.34 (s, 9H).
^{13}C NMR (101 MHz, $CDCl_3$) δ 166.0, 151.5, 141.5, 133.0, 130.8, 129.8, 128.5, 128.4, 125.1, 123.3, 123.2, 73.4, 34.9, 31.5, 22.7.
HRMS (EI+) calc: [M^+] ($C_{19}H_{22}O_2$) 282.1614, measured 282.1614, 0.00 ppm error.
IR (neat) ν_{max}/cm^{-1}: 3063, 2963, 2902, 2868, 1716, 1603, 1451, 1265, 1109, 1065.

*1-(3-(tert-Butyl)phenyl)ethyl 4-fluorobenzoate (**3b**)*

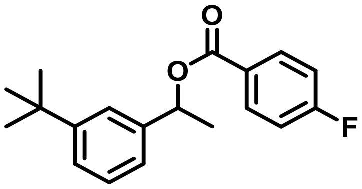

Title compound **3b** was prepared via general procedure 2 with 3 equivalents of 4-fluorobenzoic acid and isolated by column chromatography (2% ethyl acetate in pentane) to afford a pale-yellow oil (32.1 mg, 53%) after 3 *F*.

^{1}H NMR (400 MHz, $CDCl_3$) δ 8.14 – 8.05 (m, 2H), 7.44 (s, 1H), 7.39 – 7.26 (m, 3H), 7.16 – 7.07 (m, 2H), 6.13 (q, *J* = 6.6 Hz, 1H), 1.68 (d, *J* = 6.6 Hz, 3H), 1.33 (s, 9H).
^{13}C NMR (101 MHz, $CDCl_3$) δ 165.9 (d, *J* = 253.7 Hz), 165.0, 151.6, 141.4, 132.3 (d, *J* = 9.2 Hz), 128.4, 127.0 (d, *J* = 2.9 Hz), 125.2, 123.3, 123.2, 115.6 (d, *J* = 21.9 Hz), 73.6, 34.9, 31.5, 22.6.
^{19}F NMR (376 MHz, $CDCl_3$) δ -105.8 (tt, *J* = 8.4, 5.4 Hz).
HRMS (EI+) calc: [M^+] ($C_{19}H_{21}O_2F$) 300.1520, measured 300.1518, 0.67 ppm error.
IR (neat) ν_{max}/cm^{-1}: 2963, 2869, 1719, 1604, 1507, 1268, 1152, 1090, 854, 767, 706.

*1-(3-(tert-Butyl)phenyl)ethyl propionate (**3c**)*

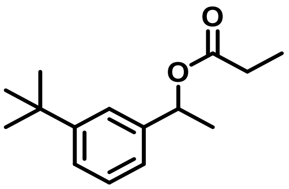

Title compound **3c** was prepared via general procedure 2 with 10 equivalents of propionic acid and isolated by column chromatography (2% ethyl acetate in pentane) to afford a light-yellow oil (30.8 mg, 66%) after 2.5 *F*.

^{1}H NMR (400 MHz, $CDCl_3$) δ 7.37 – 7.26 (m, 3H), 7.18 (dt, *J* = 7.2, 1.4 Hz, 1H), 5.90 (q, *J* = 6.6 Hz, 1H), 2.36 (qd, *J* = 7.6, 1.7 Hz, 2H), 1.53 (d, *J* = 6.6 Hz, 3H), 1.32 (s, 9H), 1.15 (t, *J* = 7.6 Hz, 3H).
^{13}C NMR (101 MHz, $CDCl_3$) δ 173.9, 151.5, 141.6, 128.3, 125.0, 123.2, 123.2, 72.5, 34.9, 31.5, 28.1, 22.5, 9.3.
HRMS (EI+) calc: [M$^+$] ($C_{15}H_{22}O_2$) 234.1614, measured 234.1613, 0.43 ppm error.
IR (neat) ν_{max}/cm^{-1}: 2965, 2882, 1737, 1606, 1462, 1364, 1183, 1065, 795, 707.

*1-(3-(tert-Butyl)phenyl)ethyl cyclopropanecarboxylate (**3d**)*

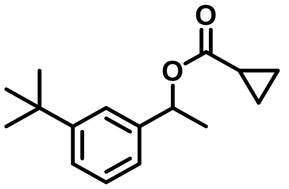

Title compound **3d** was prepared via general procedure 2 with 10 equivalents of cyclopropane carboxylic acid and isolated by column chromatography (2% ethyl acetate in pentane) to afford a colourless oil (23 mg, 47%) after 4 *F*.

^{1}H NMR (400 MHz, $CDCl_3$) δ 7.38 – 7.27 (m, 3H), 7.18 (dd, *J* = 7.2, 1.7 Hz, 1H), 5.90 (q, *J* = 6.6 Hz, 1H), 1.65 (tt, *J* = 8.0, 4.6 Hz, 1H), 1.54 (d, *J* = 6.6 Hz, 3H), 1.33 (s, 9H), 1.06 – 0.94 (m, 2H), 0.91 – 0.79 (m, 2H).
^{13}C NMR (101 MHz, $CDCl_3$) δ 174.3, 151.5, 141.6, 128.3, 125.0, 123.2, 123.2, 72.7, 34.9, 31.5, 22.5, 13.4, 8.5.
HRMS (EI+) calc: [M$^+$] ($C_{16}H_{22}O_2$) 246.1614, measured 246.1615, 0.41 ppm error.
IR (neat) ν_{max}/cm^{-1}: 2964, 2868, 1726, 1606, 1391, 1264, 1172, 1065, 795, 707.

*1-(3-(tert-Butyl)phenyl)ethyl cyclobutanecarboxylate (**3e**)*

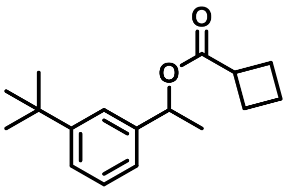

Title compound **3e** was prepared via general procedure 2 with 10 equivalents of cyclobutene carboxylic acid and isolated by column chromatography (2% ethyl acetate in pentane) to afford a colourless oil (25.1 mg, 48%) after 4 *F*.

^{1}H NMR (400 MHz, $CDCl_3$) δ 7.36 – 7.26 (m, 3H), 7.17 (dt, *J* = 7.2, 1.4 Hz, 1H), 5.90 (q, *J* = 6.6 Hz, 1H), 3.17 (p, *J* = 8.6 Hz, 1H), 2.37 – 2.15 (m, 4H), 2.07 – 1.83 (m, 2H), 1.53 (d, *J* = 6.6 Hz, 3H), 1.33 (s, 9H).
^{13}C NMR (101 MHz, $CDCl_3$) δ 174.8, 151.5, 141.8, 128.3, 124.9, 123.1, 123.0, 72.3, 38.5, 34.9, 31.5, 25.4, 25.2, 22.6, 18.5.
HRMS (EI+) calc: [M$^+$] ($C_{17}H_{24}O_2$) 260.1771, measured 260.1772, 0.38 ppm error.
IR (neat) ν_{max}/cm^{-1}: 2959, 2868, 1729, 1606, 1490, 1465, 1250, 1168, 1065, 794, 706.

*1-(3-(tert-Butyl)phenyl)ethyl 2-cyclohexylacetate (**3f**)*

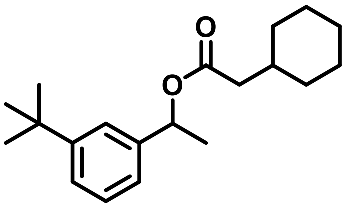

Title compound **3f** was prepared via general procedure 2 with 10 equivalents of 2-cyclohexylacetic acid and isolated by column chromatography (2% ethyl acetate in pentane) to afford a colourless oil (48 mg, 87%) after 3 *F*.

^{1}H NMR (400 MHz, $CDCl_3$) δ 7.38 – 7.26 (m, 3H), 7.17 (dd, *J* = 7.3, 1.6 Hz, 1H), 5.91 (q, *J* = 6.6 Hz, 1H), 2.22 (d, *J* = 7.1 Hz, 2H), 1.86 – 1.74 (m, 1H), 1.74 – 1.61 (m, 5H), 1.54 (d, *J* = 6.6 Hz, 3H), 1.33 (s, 9H), 1.25 (m, 2H), 1.15 (m, 1H), 0.96 (m, 2H).
^{13}C NMR (101 MHz, $CDCl_3$) δ 172.6, 151.5, 141.6, 128.3, 124.9, 123.3, 123.2, 72.4, 42.6, 35.1, 34.8, 33.2, 33.1, 31.5, 26.3, 26.2, 22.5.
HRMS (EI+) calc: [M$^+$] ($C_{20}H_{30}O_2$) 302.2240, measured 302.2242, 0.66 ppm error.
IR (neat) ν_{max}/cm^{-1}: 2963, 2923, 2852, 1732, 1606, 1449, 1363, 1286, 1236, 1190, 1163, 1065, 794, 706.

*1-(3-(tert-Butyl)phenyl)ethyl-adamantane-1-carboxylate (**3g**)*

Title compound **3g** was prepared via general procedure 2 with 5 equivalents of adamantane-1-carboxylic acid and isolated by column chromatography (2% ethyl acetate in pentane) to afford a colourless oil (48 mg, 70%) after 3 *F*.

^{1}H NMR (400 MHz, $CDCl_3$) δ 7.36 – 7.27 (m, 3H), 7.16 (dd, *J* = 7.3, 1.7 Hz, 1H), 5.87 (q, *J* = 6.6 Hz, 1H), 2.03 (s, 3H), 1.93 (d, *J* = 2.8 Hz, 6H), 1.78 – 1.68 (m, 6H), 1.51 (d, *J* = 6.6 Hz, 3H), 1.33 (s, 9H).
^{13}C NMR (101 MHz, $CDCl_3$) δ 177.0, 151.4, 142.1, 128.2, 124.6, 122.9, 122.8, 71.9, 40.8, 38.9, 36.7, 34.9, 31.5, 28.1, 22.8.
HRMS (EI+) calc: [M^+] ($C_{23}H_{32}O_2$) 340.2397, measured 340.2397, 0.00 ppm error.
IR (neat) ν_{max}/cm^{-1}: 2904, 2851, 1725, 1605, 1452, 1230, 1073, 793, 705.

*1-(3-(tert-Butyl)phenyl)ethyl pivalate (**3h**)*

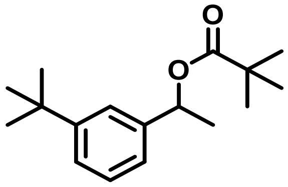

Title compound **3h** was prepared via general procedure 2 with 10 equivalents of pivalic acid and isolated by column chromatography (2% ethyl acetate in pentane) to afford a yellow oil (35.8 mg, 68%) after 3 *F*.

^{1}H NMR (400 MHz, $CDCl_3$) δ 7.36 – 7.27 (m, 3H), 7.15 (dt, *J* = 7.0, 1.7 Hz, 1H), 5.86 (q, *J* = 6.6 Hz, 1H), 1.51 (d, *J* = 6.6 Hz, 3H), 1.32 (s, 9H), 1.22 (s, 9H).
^{13}C NMR (101 MHz, $CDCl_3$) δ 177.8, 151.4, 142.0, 128.2, 124.7, 122.9, 122.7, 72.3, 38.9, 34.9, 31.5, 27.3, 22.7.
HRMS (EI+) calc: [M^+] ($C_{17}H_{26}O_2$) 262.1927, measured 262.1926, 0.38 ppm error.
IR (neat) ν_{max}/cm^{-1}: 2964, 2871, 1729, 1480, 1366, 1281, 1156, 1065, 795, 706.

*1-(3-(tert-Butyl)phenyl)ethyl 5-bromopentanoate (**3i**)*

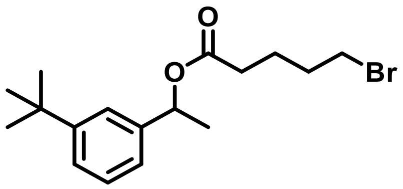

Title compound **3i** was prepared via general procedure 2 with 10 equivalents of 5-bromovaleric acid and isolated by column chromatography (2% ethyl acetate in pentane) to afford a colourless oil (41 mg, 61%) after 4 *F*.

^{1}H NMR (400 MHz, $CDCl_3$) δ 7.36 – 7.26 (m, 3H), 7.17 (dt, *J* = 7.3, 1.4 Hz, 1H), 5.90 (q, *J* = 6.6 Hz, 1H), 3.39 (t, *J* = 6.5 Hz, 2H), 2.37 (td, *J* = 7.2, 1.2 Hz, 2H), 1.93 – 1.74 (m, 4H), 1.54 (d, *J* = 6.6 Hz, 3H), 1.32 (s, 9H).
^{13}C NMR (101 MHz, $CDCl_3$) δ 172.5, 151.5, 141.4, 128.4, 125.1, 123.3, 123.2, 72.8, 34.9, 33.7, 33.2, 32.1, 31.5, 23.7, 22.5.
HRMS (EI+) calc: [M^{+}] ($C_{17}H_{25}O_2Br$) 340.1032, measured 340.1030, 0.59 ppm error.
IR (neat) ν_{max}/cm^{-1}: 2962, 2868, 1732, 1606, 1454, 1366, 1254, 1201, 1171, 1065, 796, 707.

*1-(3-(tert-Butyl)phenyl)ethyl 2,2,2-trifluoroacetate (**3j**)*

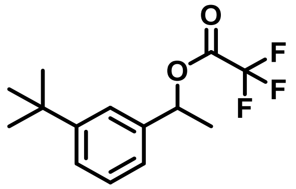

Title compound **3j** was prepared via general procedure 2 with 10 equivalents of trifluoroacetic acid and isolated by column chromatography (2% ethyl acetate in pentane) to afford a colourless oil (22 mg, 40%) after 3 *F*.

^{1}H NMR (400 MHz, $CDCl_3$) δ 7.41 – 7.30 (m, 3H), 7.20 (dt, *J* = 7.5, 1.3 Hz, 1H), 6.04 (q, *J* = 6.6 Hz, 1H), 1.68 (d, *J* = 6.6 Hz, 3H), 1.33 (s, 9H).
^{13}C NMR (126 MHz, $CDCl_3$) δ 156.9 (q, *J* = 42.1 Hz), 152.0, 138.9, 128.7, 126.0, 123.3, 123.2, 114.7 (q, *J* = 286.0 Hz), 77.7, 34.9, 31.4, 22.1.
^{19}F NMR (376 MHz, $CDCl_3$) δ -75.2 (s).
HRMS (Nanospray) calc: [M+Na]$^{+}$ ($C_{14}H_{17}O_2NaF_3$) 297.1078, measured 297.1083, 1.68 ppm error.
IR (neat) ν_{max}/cm^{-1}: 2967, 1783, 1607, 1376, 1222, 1157, 1062, 705.

*1-(3-(tert-Butyl)phenyl)ethyl acrylate (**3k**)*

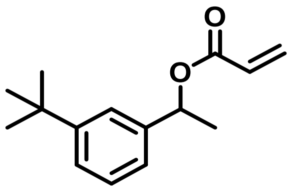

Title compound **3k** was prepared via general procedure 2 with 10 equivalents of acrylic acid and isolated by column chromatography (2% ethyl acetate in pentane) to afford a white solid (17.8 mg, 38%) after 3 *F*.

^{1}H NMR (400 MHz, $CDCl_3$) δ 7.39 – 7.27 (m, 3H), 7.20 (dt, *J* = 7.3, 1.4 Hz, 1H), 6.43 (dd, *J* = 17.3, 1.5 Hz, 1H), 6.16 (dd, *J* = 17.3, 10.4 Hz, 1H), 5.97 (q, *J* = 6.6 Hz, 1H), 5.83 (dd, *J* = 10.4, 1.5 Hz, 1H), 1.58 (d, *J* = 6.6 Hz, 3H), 1.32 (s, 9H).
^{13}C NMR (126 MHz, $CDCl_3$) δ 165.7, 151.5, 141.3, 130.8, 129.0, 128.4, 125.1, 123.3, 123.3, 73.0, 34.9, 31.5, 22.5.
HRMS (EI+) calc: [M+] ($C_{15}H_{20}O_2$) 232.1458, measured 232.1457, 0.43 ppm error.
IR (neat) ν_{max}/cm^{-1}: 2962, 2926, 1727, 1404, 1294, 1266, 1190, 1066, 809, 707.

*1-(3-(tert-Butyl)phenyl)ethyl (E)-but-2-enoate (**3l**)*

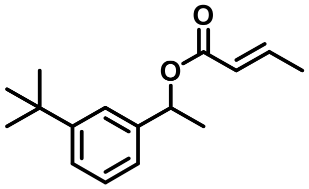

Title compound **3l** was prepared via general procedure 2 with 10 equivalents of crotonic acid and isolated by column chromatography (2% ethyl acetate in pentane) to afford a colourless oil (33.5 mg, 68%) after 3.5 *F*.

^{1}H NMR (400 MHz, $CDCl_3$) δ 7.38 – 7.27 (m, 3H), 7.20 (dt, *J* = 7.2, 1.5 Hz, 1H), 7.00 (dq, *J* = 15.5, 6.9 Hz, 1H), 5.96 (q, *J* = 6.6 Hz, 1H), 5.89 (dq, *J* = 15.5, 1.7 Hz, 1H), 1.88 (dd, *J* = 6.9, 1.7 Hz, 3H), 1.57 (d, *J* = 6.6 Hz, 3H), 1.33 (s, 9H).
^{13}C NMR (101 MHz, $CDCl_3$) δ 166.0, 151.4, 144.8, 141.6, 128.3, 125.0, 123.3, 123.2, 123.2, 72.4, 34.8, 31.5, 22.5, 18.1.
HRMS (EI+) calc: [M^+] ($C_{16}H_{22}O_2$) 246.1614, measured 246.1613, 0.41 ppm error.
IR (neat) ν_{max}/cm^{-1}: 2964, 2868, 1717, 1657, 1444, 1292, 1262, 1179, 1101, 1065, 968, 795, 706.

Acyloxylation Failed or Low Yielding Carboxylic Acids

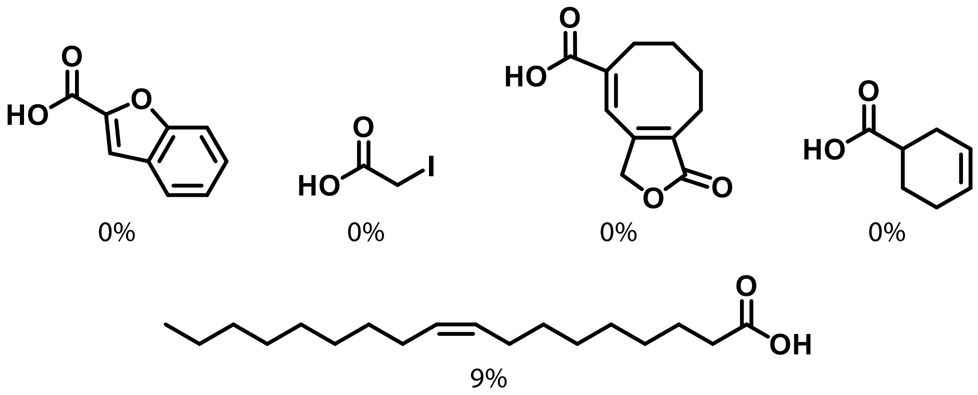

Figure S4. Failed or low yielding carboxylic acids.

Electrochemical Flow Procedure and Optimisation

Electrochemical Flow Optimisation

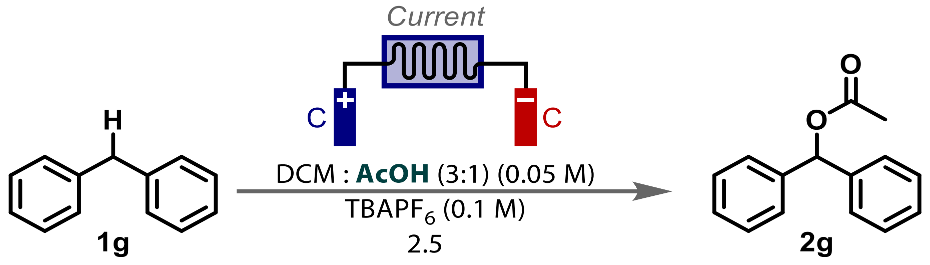

Diphenylmethane (2.50 mmol, 1.0 equiv., 418 µL) and $TBAPF_6$ (5.00 mmol, 2.0 equiv., 1.94 g) were added to an oven-dried round-bottom flask equipped with a stirrer bar and suba-seal. After purging the flask with N_2 for 5 min, DCM (37.5 mL) and glacial acetic acid (12.5 mL) were added. The reaction mixture was stirred for 5 min, then transferred to two 25-mL syringes, and attached to a syringe pump and tubing connected to the flow cell (via a mixer chip to combine the two syringes' flows). After priming the cell, several sequential constant current electrolyses were carried out at different currents (with flow rates adjusted to maintain 2.5 *F* per residence time), with 150 µL samples taken after 1.5 residence times had passed. Samples were prepared for GC-MS analysis by dilution with DCM and filtering through a short silica plug.

Current applied / mA	Potential observed/ V	Flow rate / (mL min^{-1})	Charge per residence time / *F*	P:SM[a]
19	4.6	0.092	2.5	1.37:1
37	5.8	0.184	2.5	0.83:1
56	7.0	0.276	2.5	0.58:1
74	8.3	0.369	2.5	0.37:1

[a] calculated by GCMS peak area

Recirculating Flow Procedure

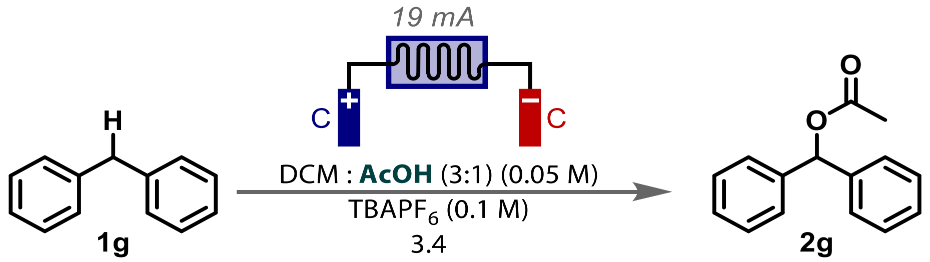

Diphenylmethane (9.00 mmol, 1.0 equiv., 1.51 mL) and $TBAPF_6$ (18.0 mmol, 2.0 equiv., 6.97 g) were added to an oven-dried round-bottom flask equipped with a stirrer bar and suba-seal. After purging the flask with N_2 for 5 min, DCM (135 mL) and glacial acetic acid (45 mL) were added under an N_2 atmosphere. After stirring for 5 min, the reagent input line of a Vapourtec E-Series Flow Chemistry System was submerged in the stirring reaction mixture and the output line positioned above the surface of the reaction mixture. After priming the flow cell, the reaction mixture was recirculated at 1 mL min^{-1} and electrolysed at 19 mA. The reaction was monitored by TLC and GC-MS and was judged to have reached completion after passing 3.4 *F*. The flow system was flushed into the round-bottomed flask with DCM (30 mL) from the solvent input line. Sat. aq. $NaHCO_3$ was added to the reaction mixture until the pH of the mixture was >7, then the organic layer was extracted three times with DCM, dried over $MgSO_4$, filtered and the solvent was removed under reduced pressure. The resulting residue was redissolved in minimal DCM, triturated with Et_2O, then filtered through a Celite pad and concentrated *in vacuo*. The crude material was purified by flash column chromatography (4% ethyl acetate in pentane) to afford **2g** (1.05 g, 51%) as a colourless oil.

Electrochemical Setup

ElectraSyn Setup

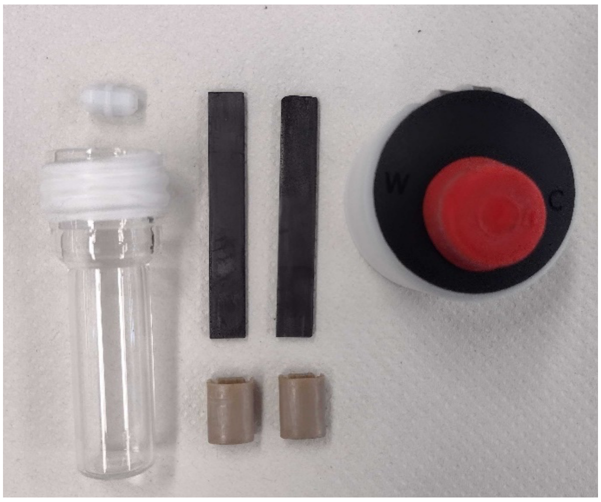

Figure S5. Items for ElectraSyn 2.0 setup. Note commercial seal replaced with a B14 septum and vial threads wrapped in PTFE tape.

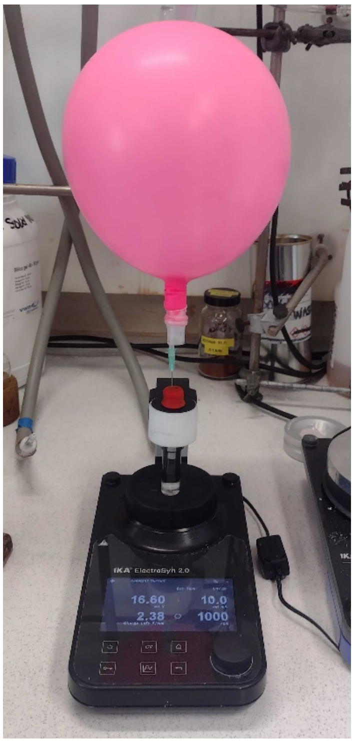

Figure S6. Reaction mixture attached to ElectraSyn 2.0 with N_2 balloon.

Flow Setup

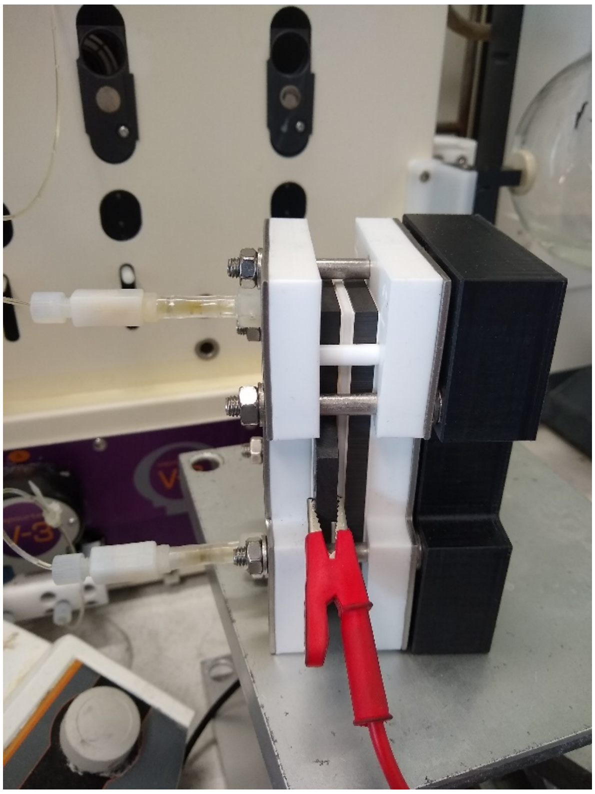

Figure S7. Electrochemical flow cell.

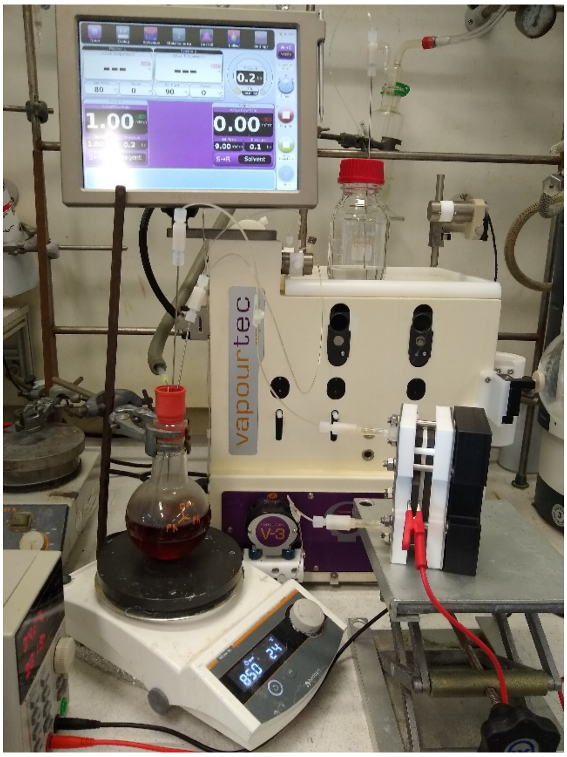

Figure S8. Complete flow setup. Cell connected to Tenma DC power supply and reaction mixture circulated by the Vapourtec E-Series Flow Chemistry System.

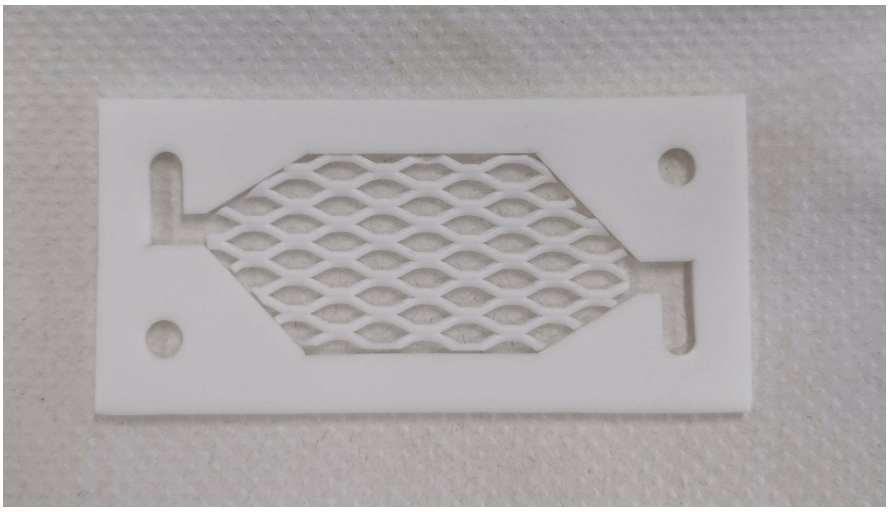

Figure S9. PTFE flow path piece. Turbulence mixing piece was included during electrolysis.

Flow electrolysis was carried out in an ElectroCell Micro Flow Cell using the 2 mm thick, 10 cm^2 electrode area PTFE flow frame and PTFE turbulence mesh, expanded PTFE gaskets, and two custom-made, impervious graphite, plate electrodes. Reaction mixtures were pumped with either a syringe pump or a Vapourtec E-Series Flow Chemistry System peristaltic pump, using a variety of rubber and plastic tubing and adapters. A Tenma DC power supply was used as a power source.

Compounds Spectra

Acetoxylation Product NMR Spectra

1-(4-Fluoro-1[1,1'-biphenyl]-4-yl)ethyl acetate (**2a**)

^{1}H NMR (400 MHz, $CDCl_3$)

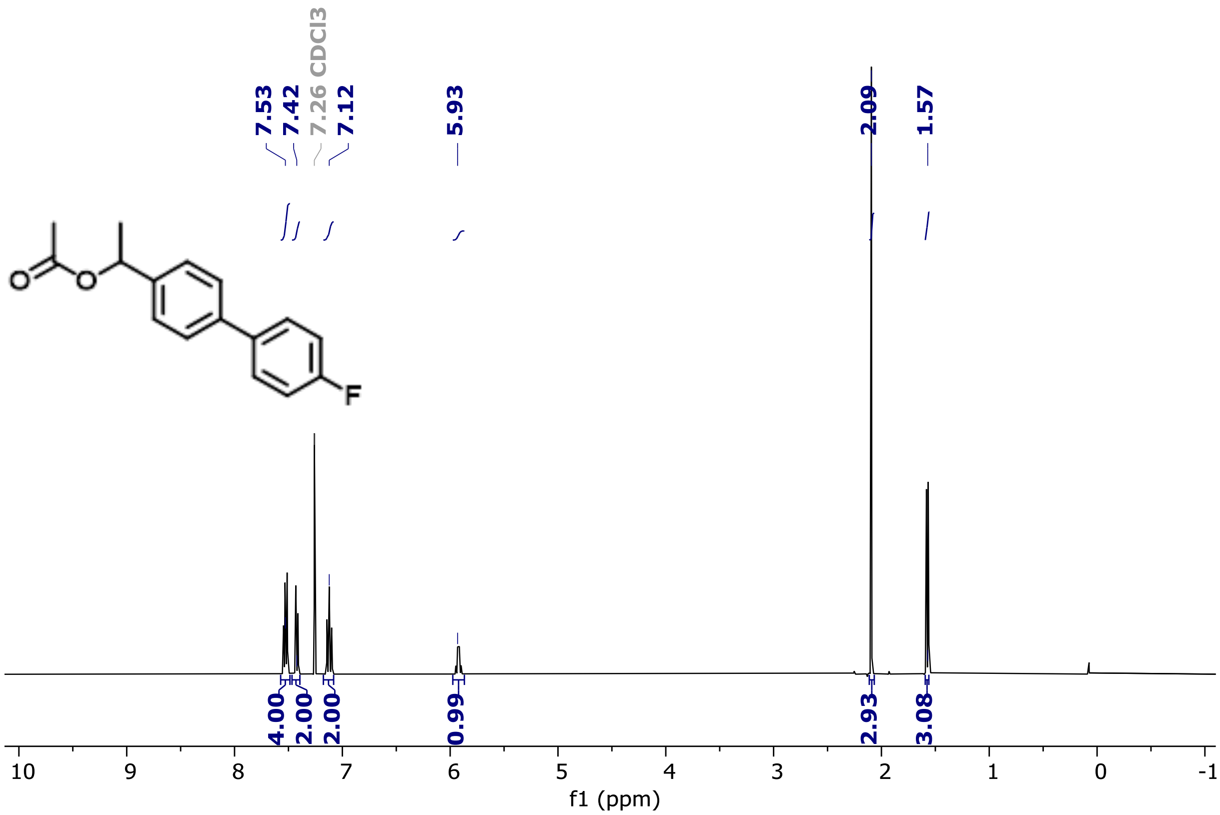

^{13}C NMR (101 MHz, $CDCl_3$)

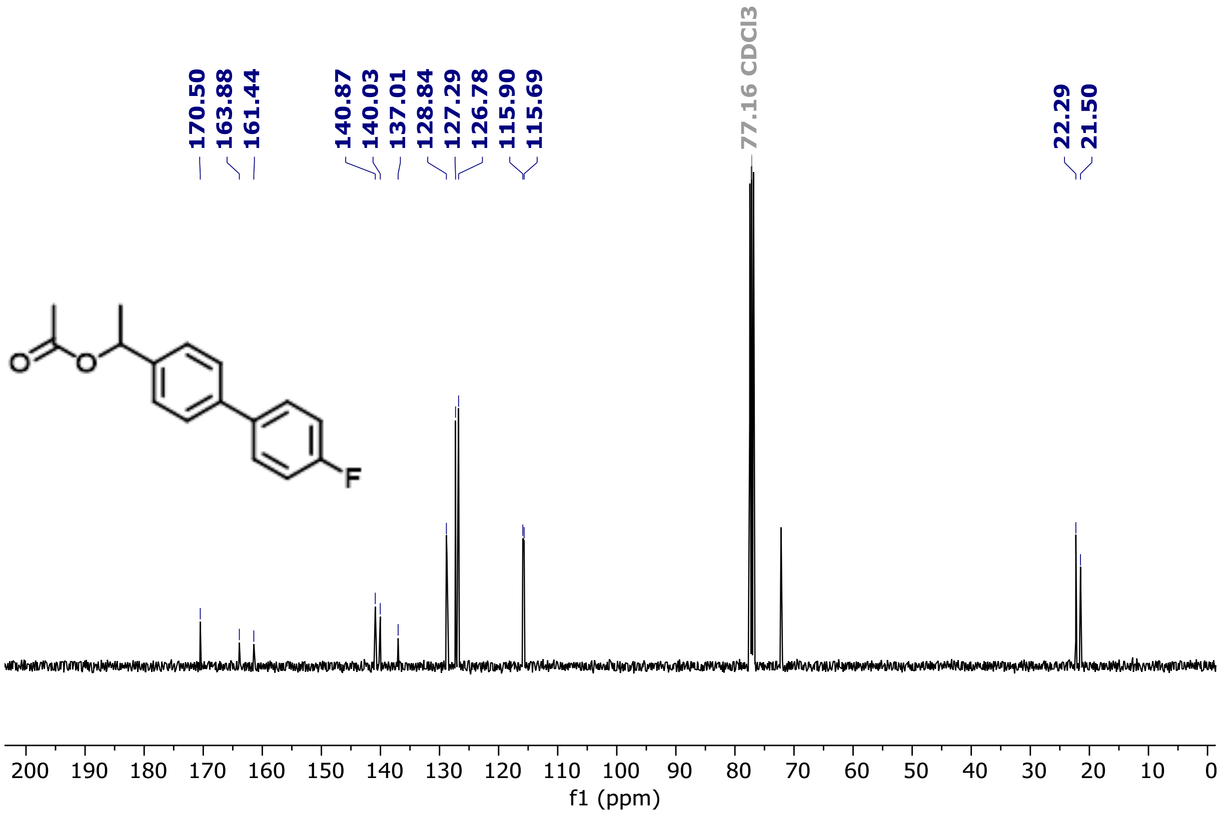

^{19}F NMR (377 MHz, $CDCl_3$)

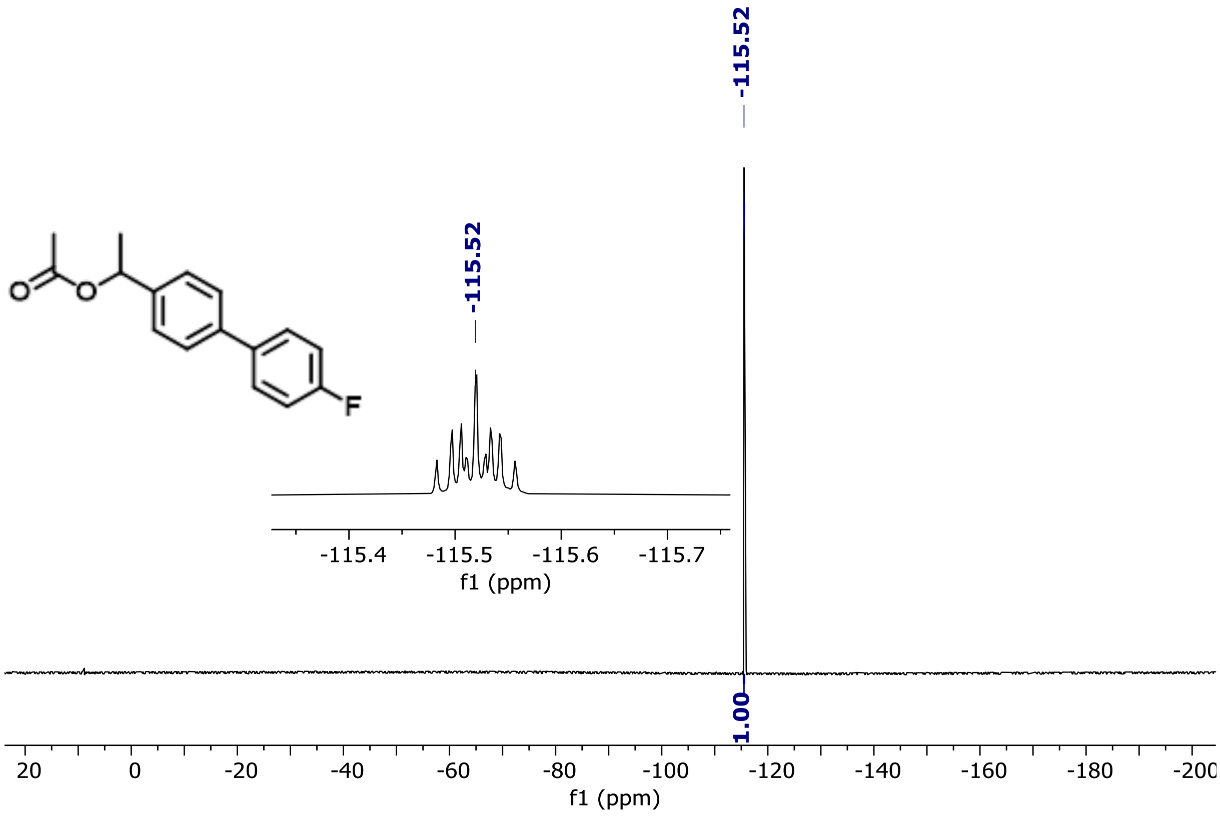

*1-(4'-Bromo-[1,1'-biphenyl]-4-yl)butyl acetate (**2b**)*

^{1}H NMR (400 MHz, $CDCl_3$)

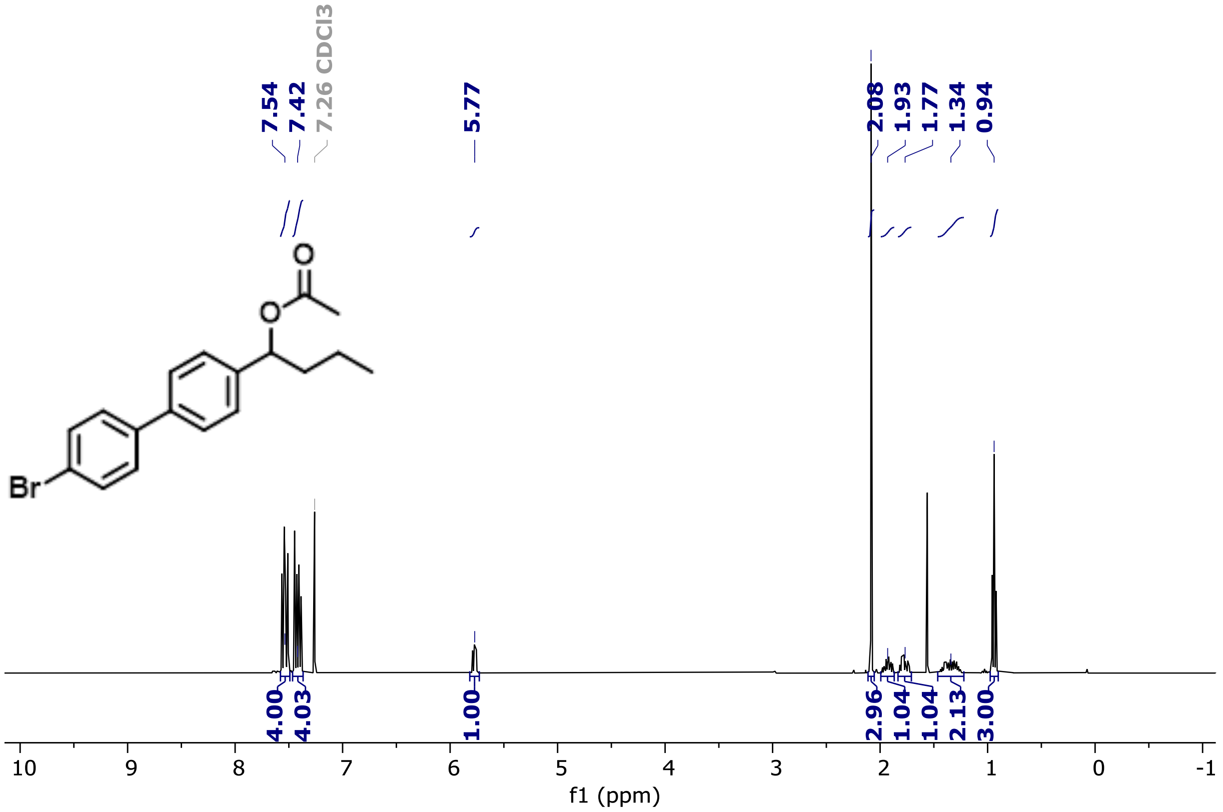

^{13}C NMR (101 MHz, $CDCl_3$)

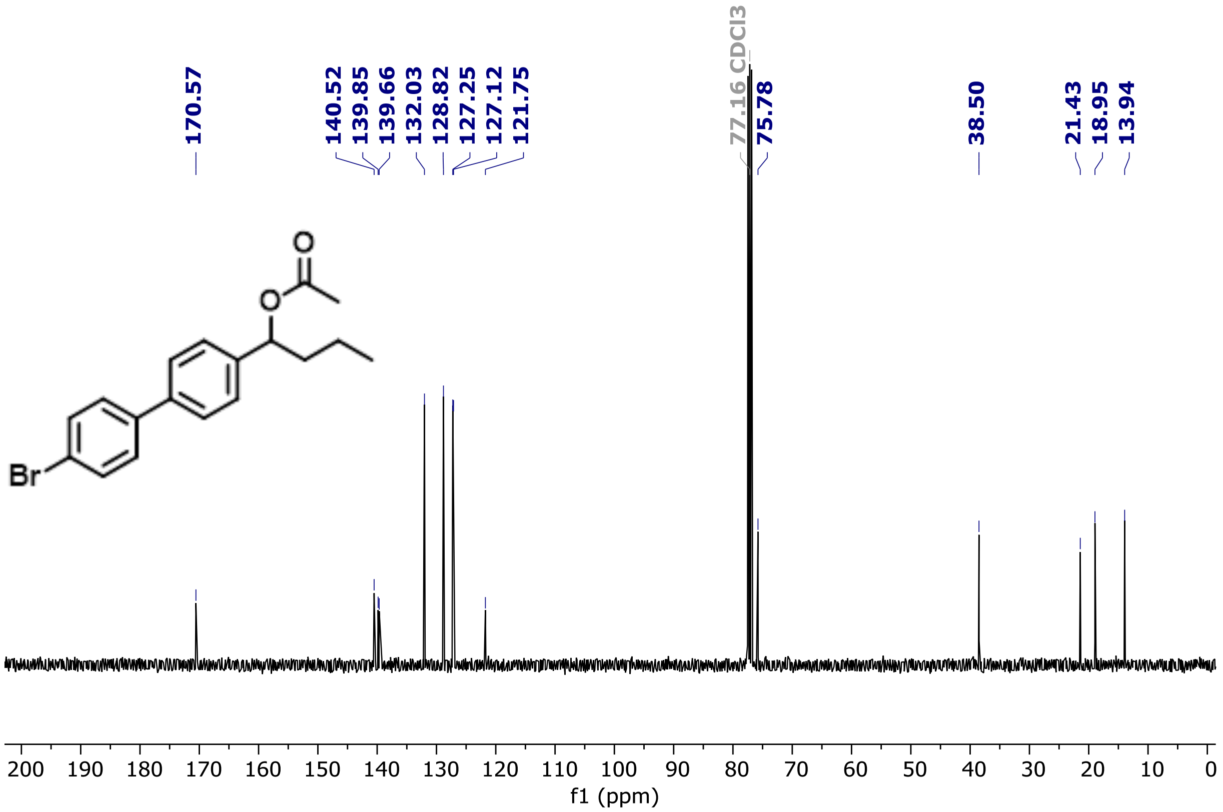

1-Phenylethyl acetate (**2c**)

^{1}H NMR (400 MHz, $CDCl_3$)

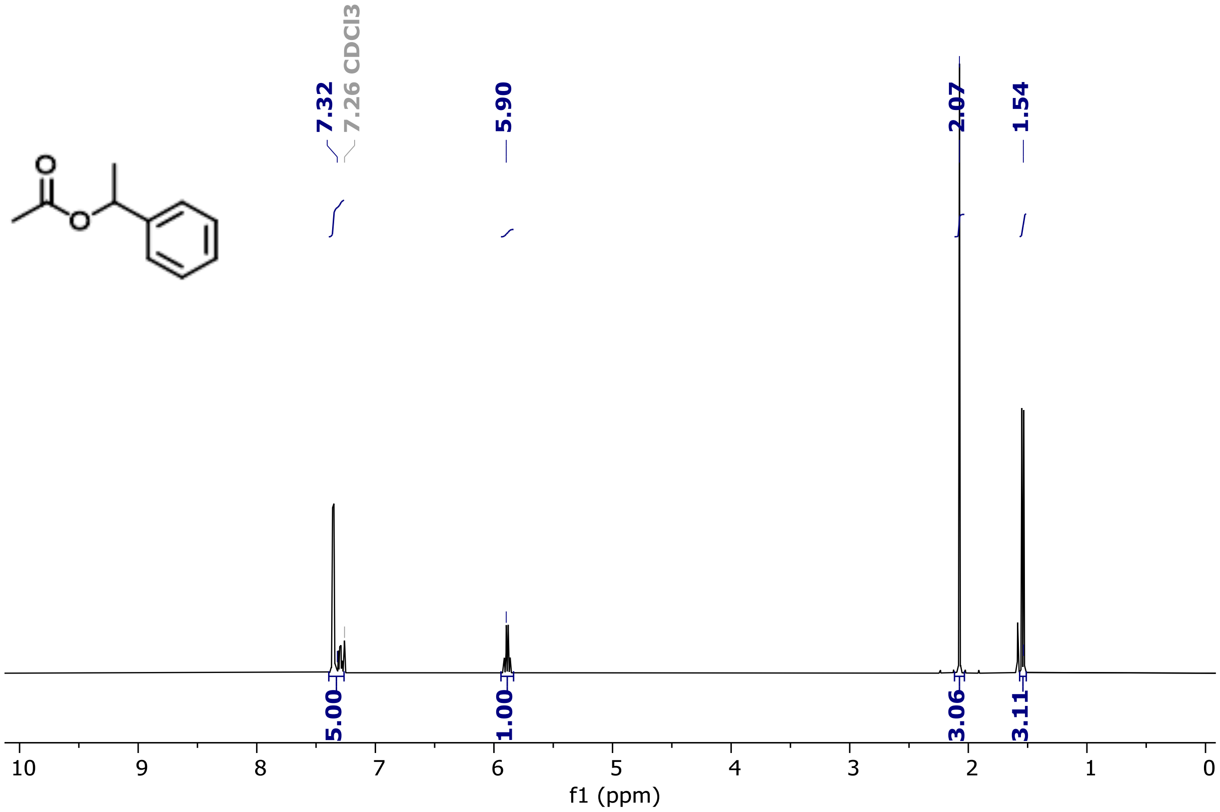

^{13}C NMR (101 MHz, $CDCl_3$)

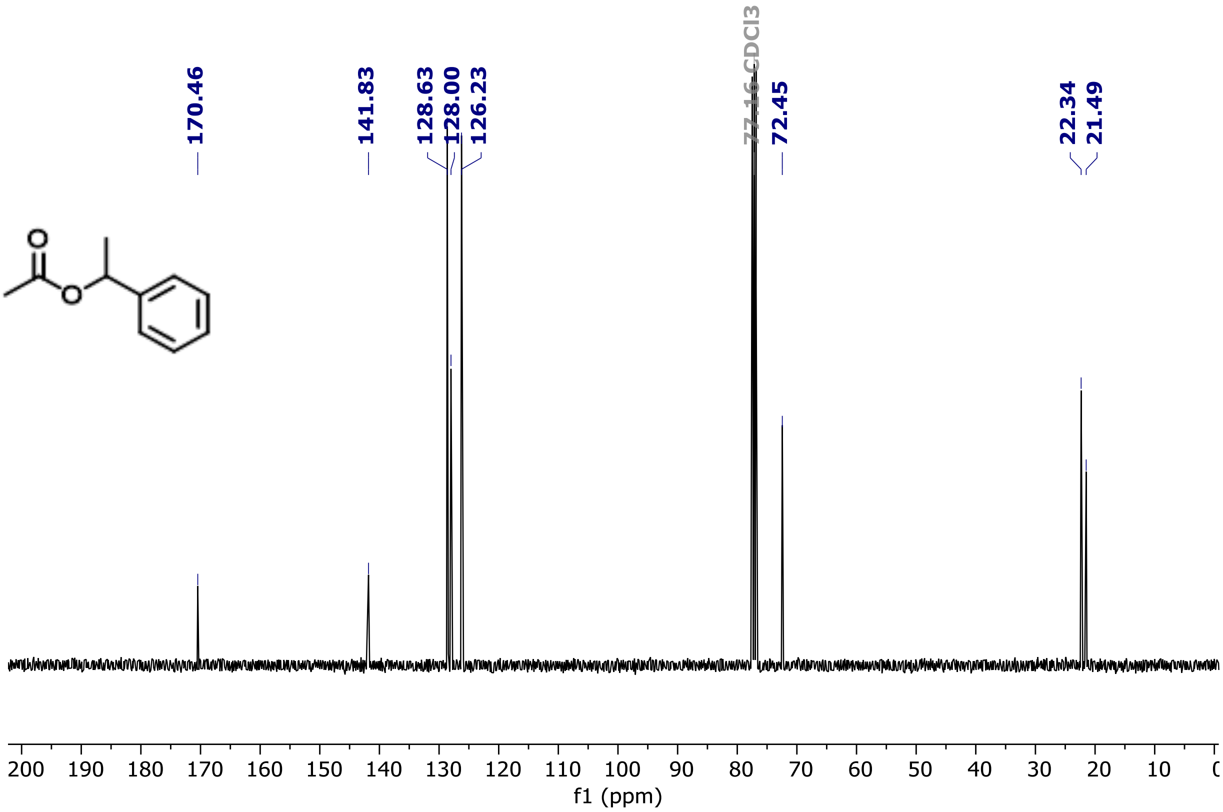

*1-(3-(tert-Butyl)phenyl)ethyl acetate (**2d**)*

^{1}H NMR (400 MHz, $CDCl_3$)

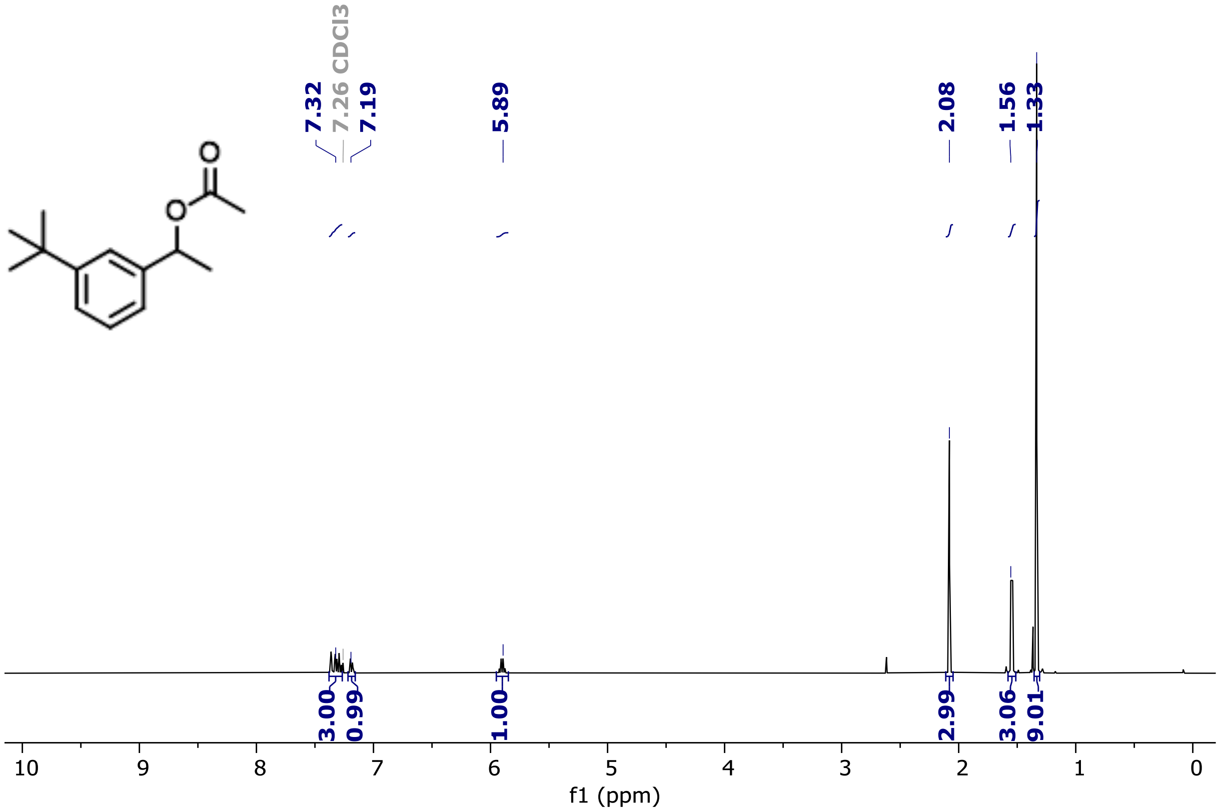

^{13}C NMR (101 MHz, $CDCl_3$)

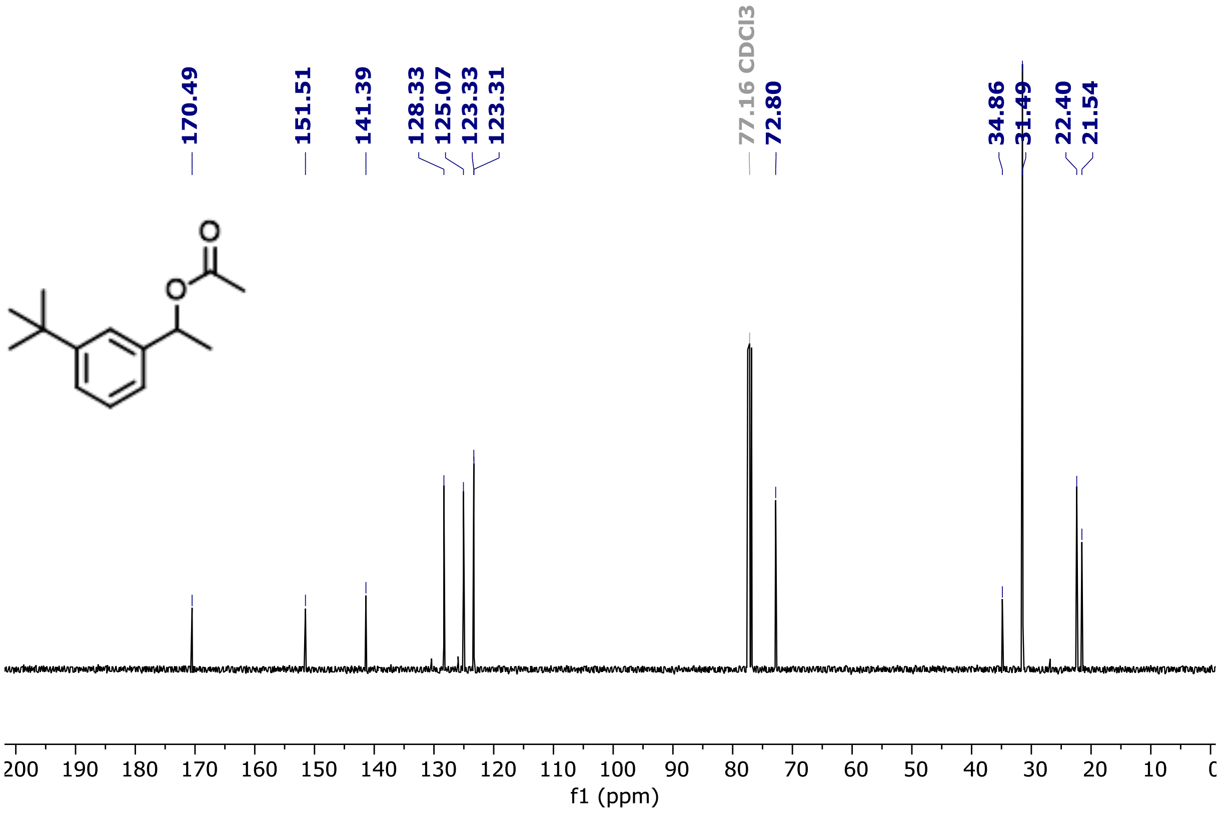

1-(4-Bromophenyl)ethyl acetate (**2e**)

^{1}H NMR (400 MHz, $CDCl_3$)

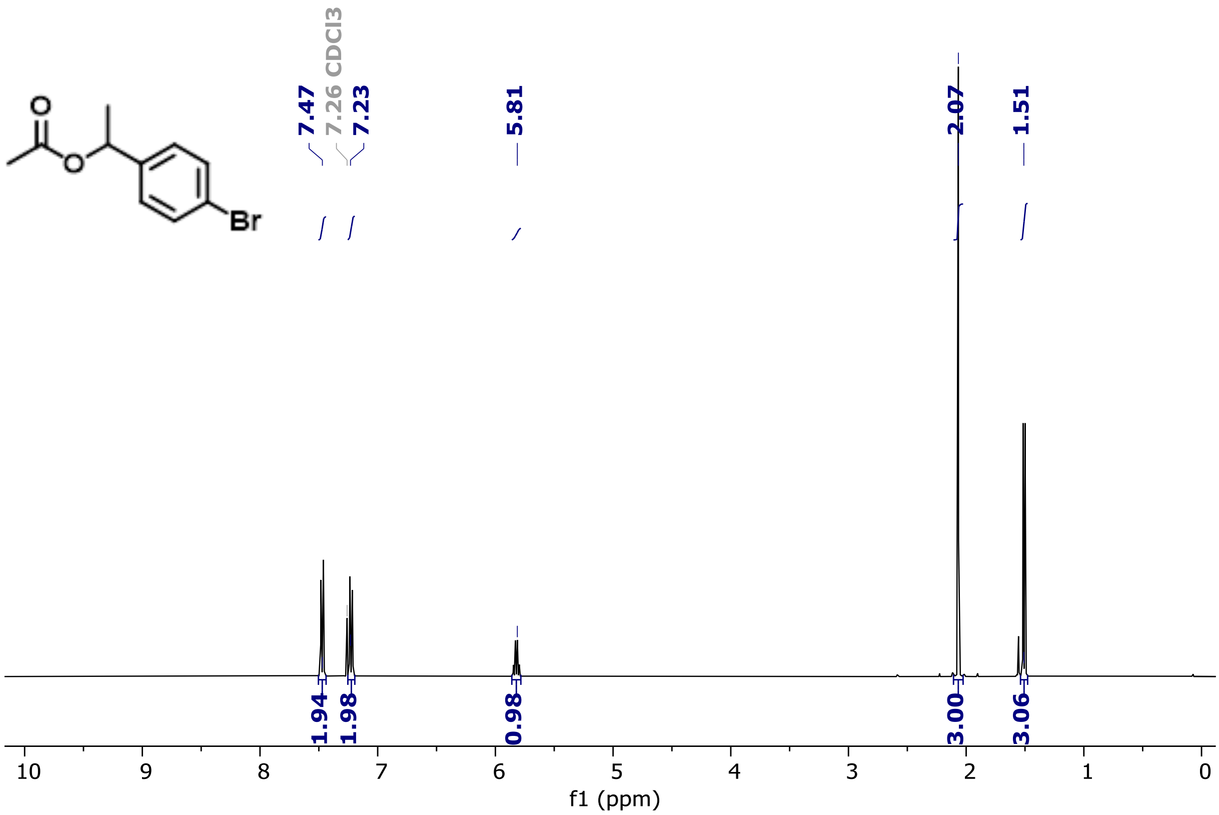

^{13}C NMR (101 MHz, $CDCl_3$)

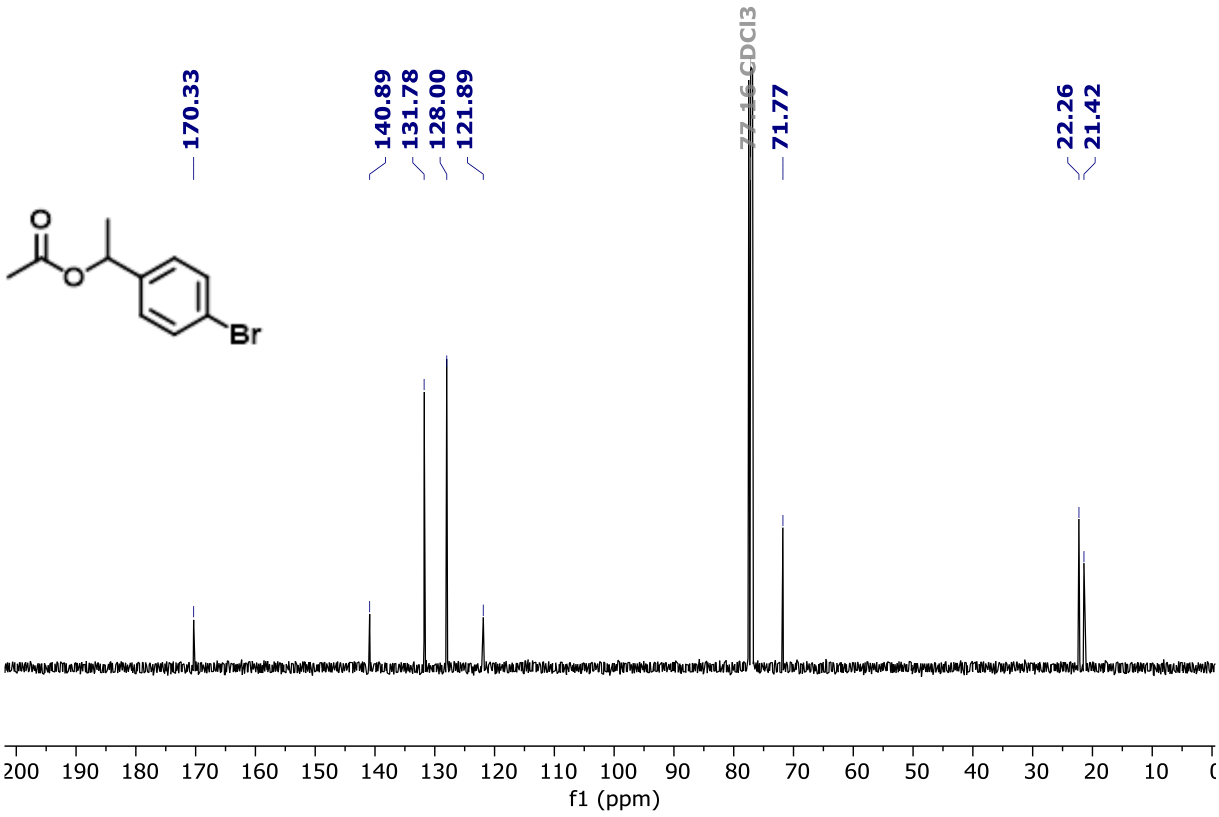

*1-(4-Bromophenyl)pentyl acetate (**2f**)*

^{1}H NMR (400 MHz, $CDCl_3$)

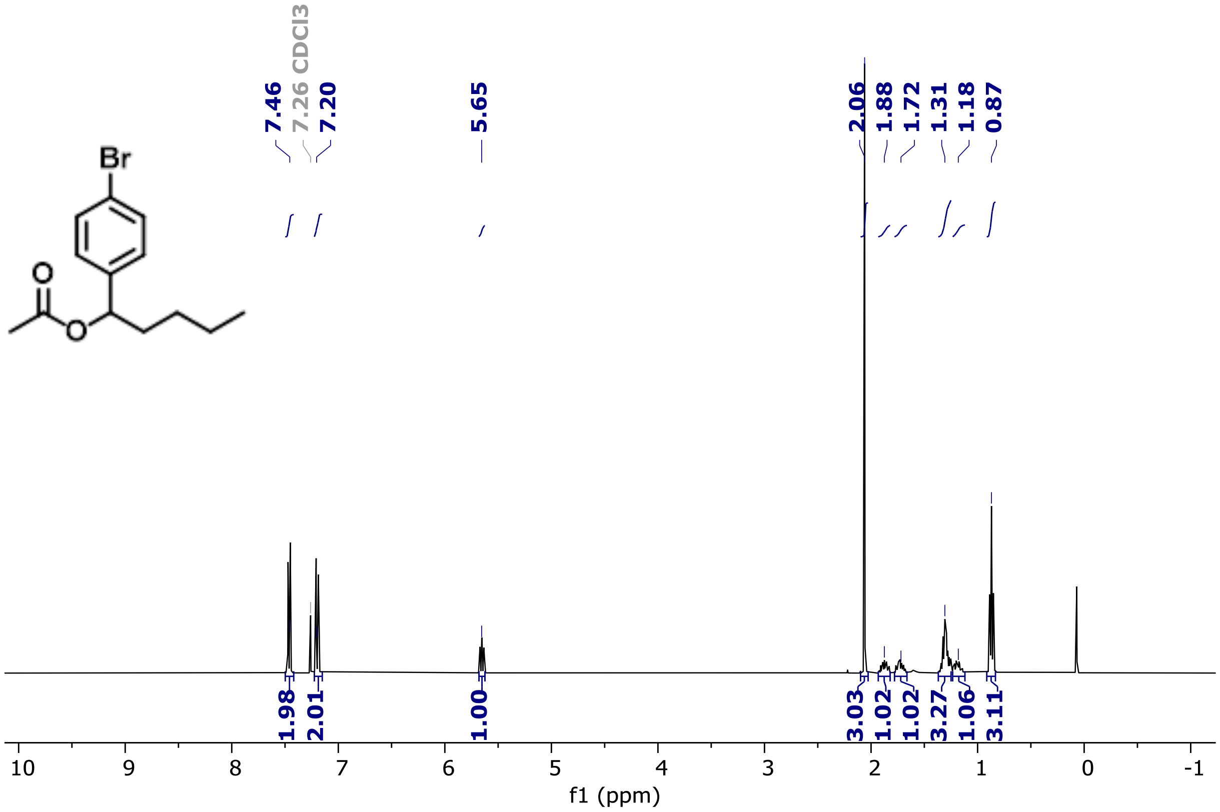

^{13}C NMR (101 MHz, $CDCl_3$)

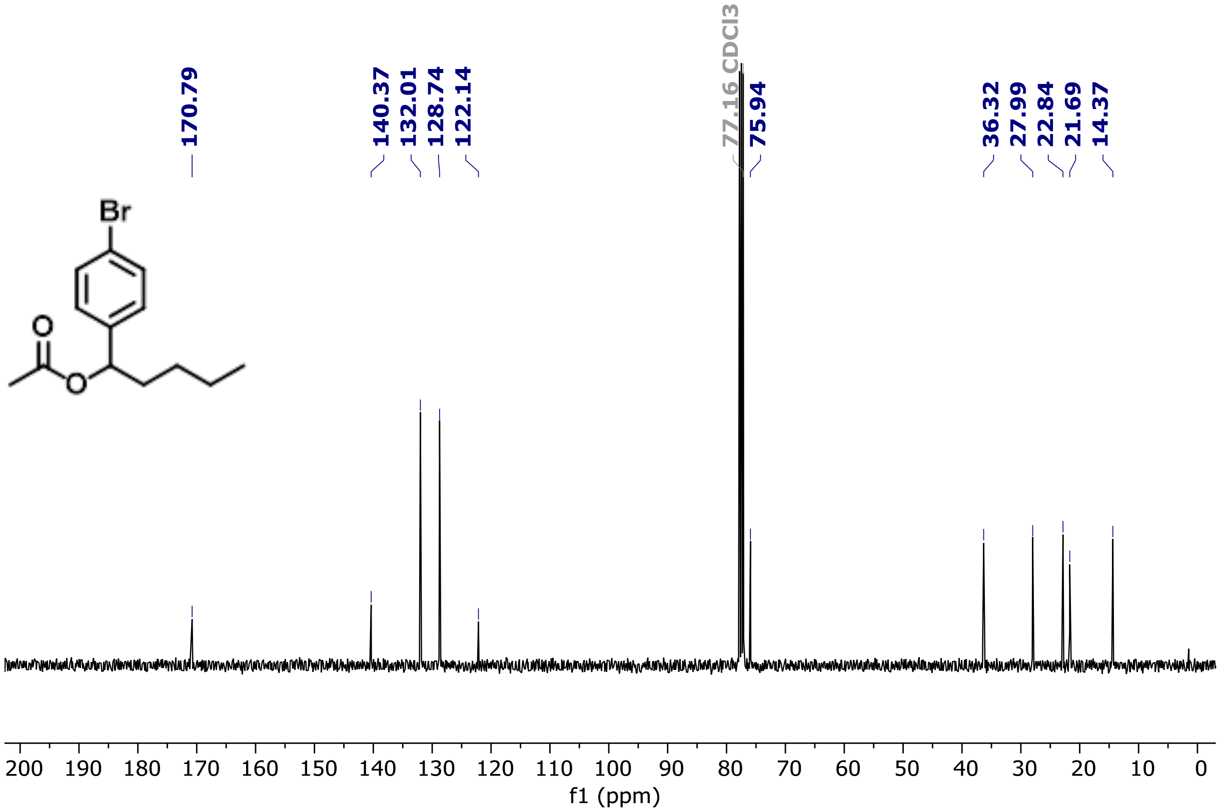

Benzhydryl acetate (**2g**)

^{1}H NMR (400 MHz, $CDCl_3$)

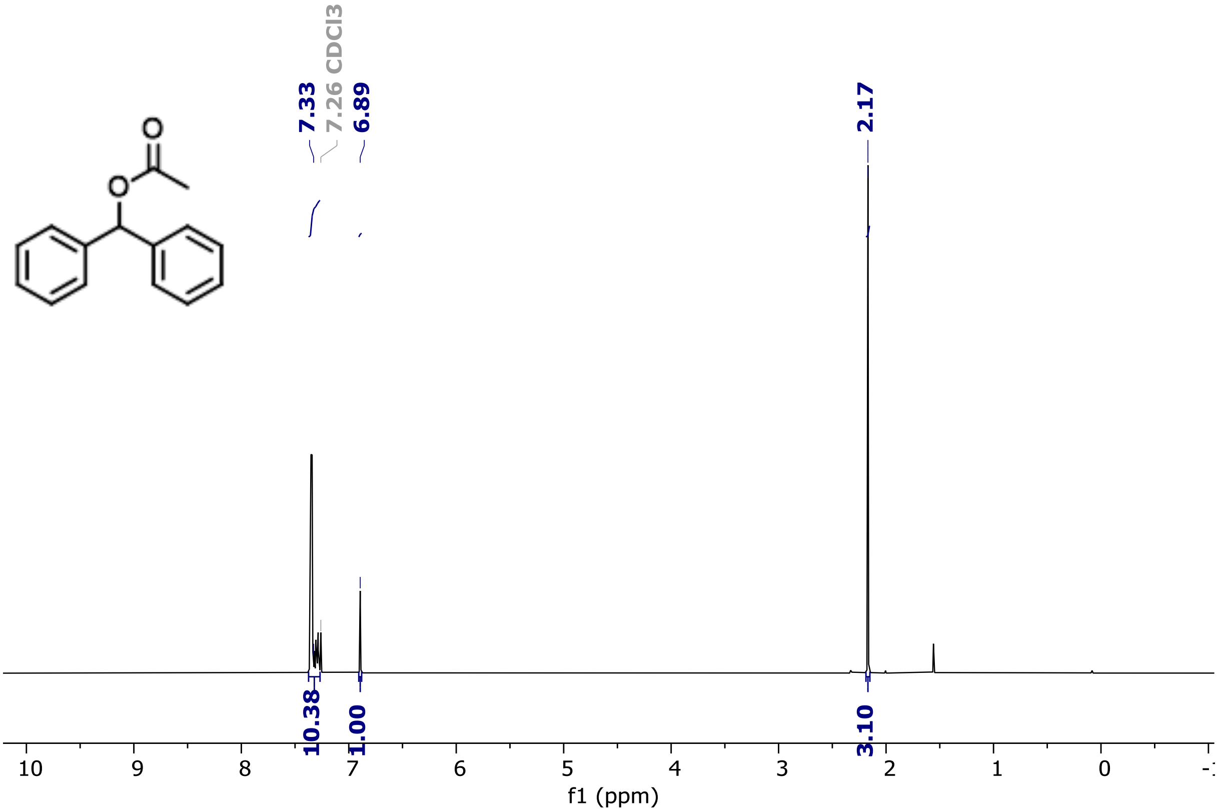

^{13}C NMR (101 MHz, $CDCl_3$)

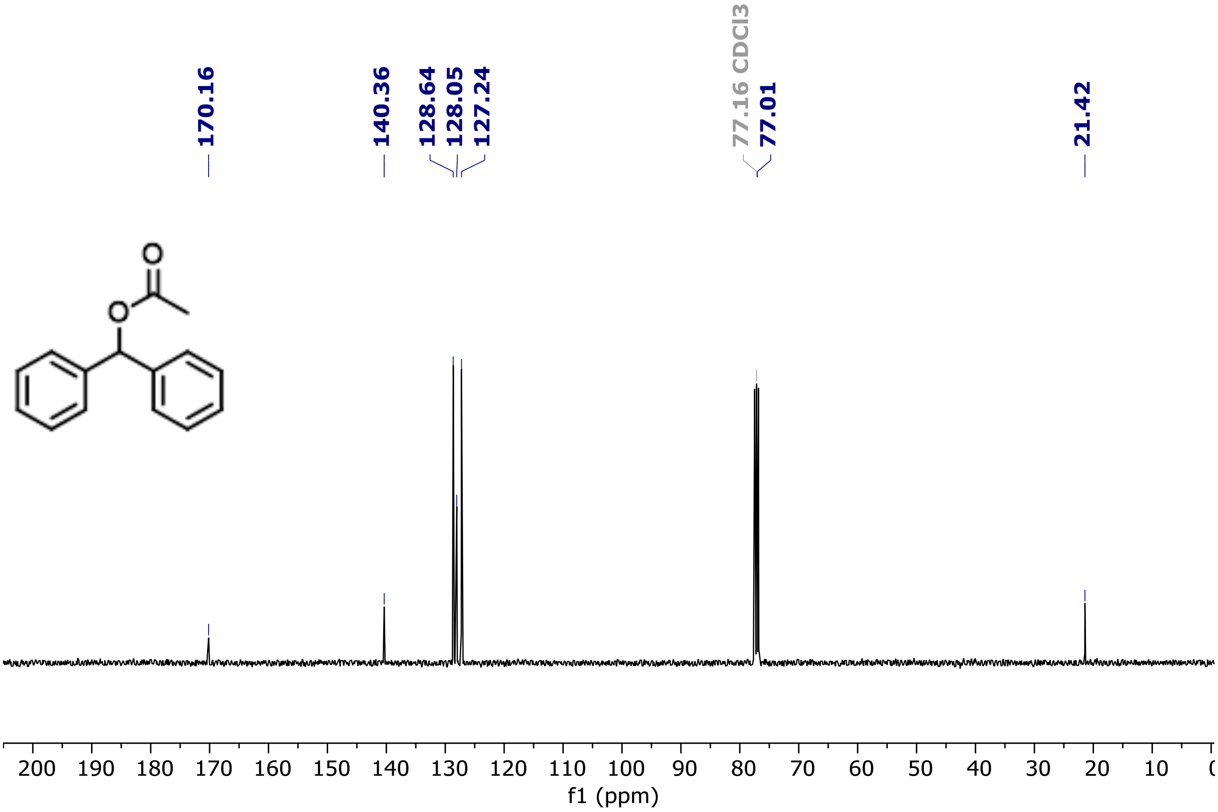

1,2,3,4-Tetrahydronaphthalen-1-yl acetate (**2h**)

^{1}H NMR (400 MHz, $CDCl_3$)

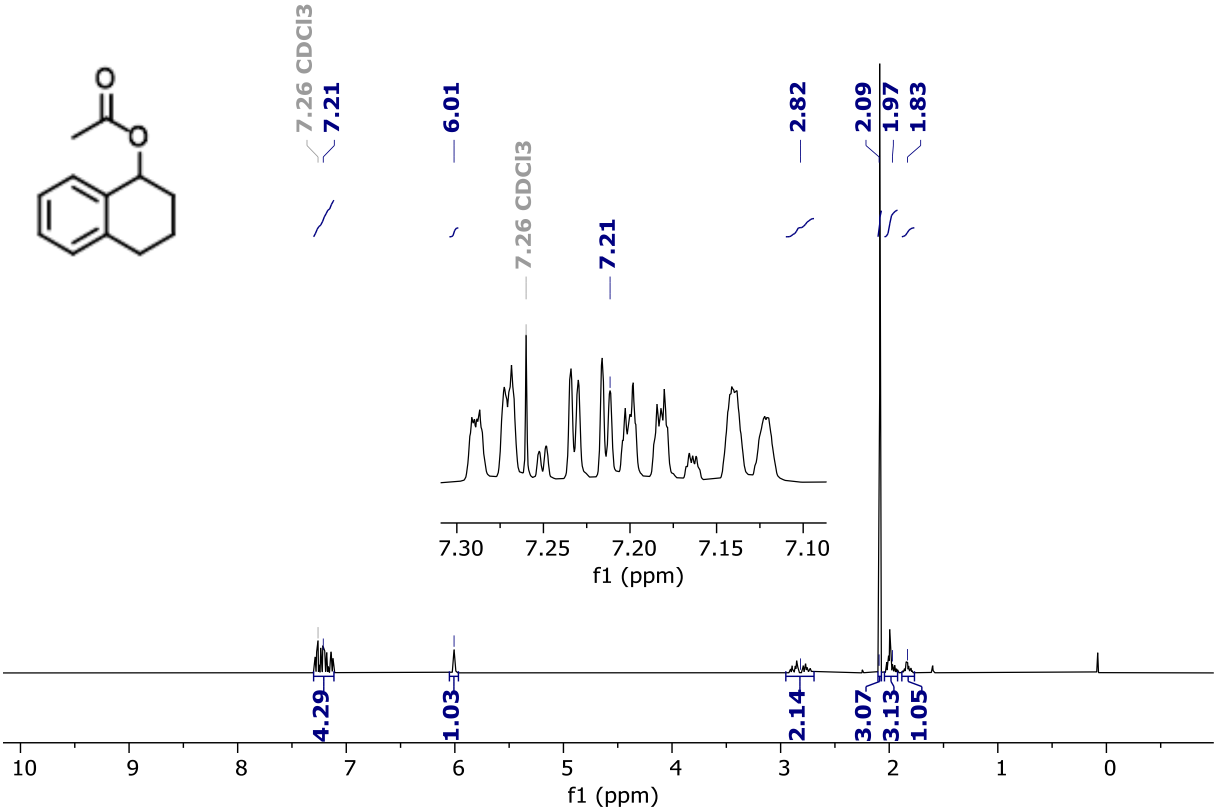

^{13}C NMR (101 MHz, $CDCl_3$)

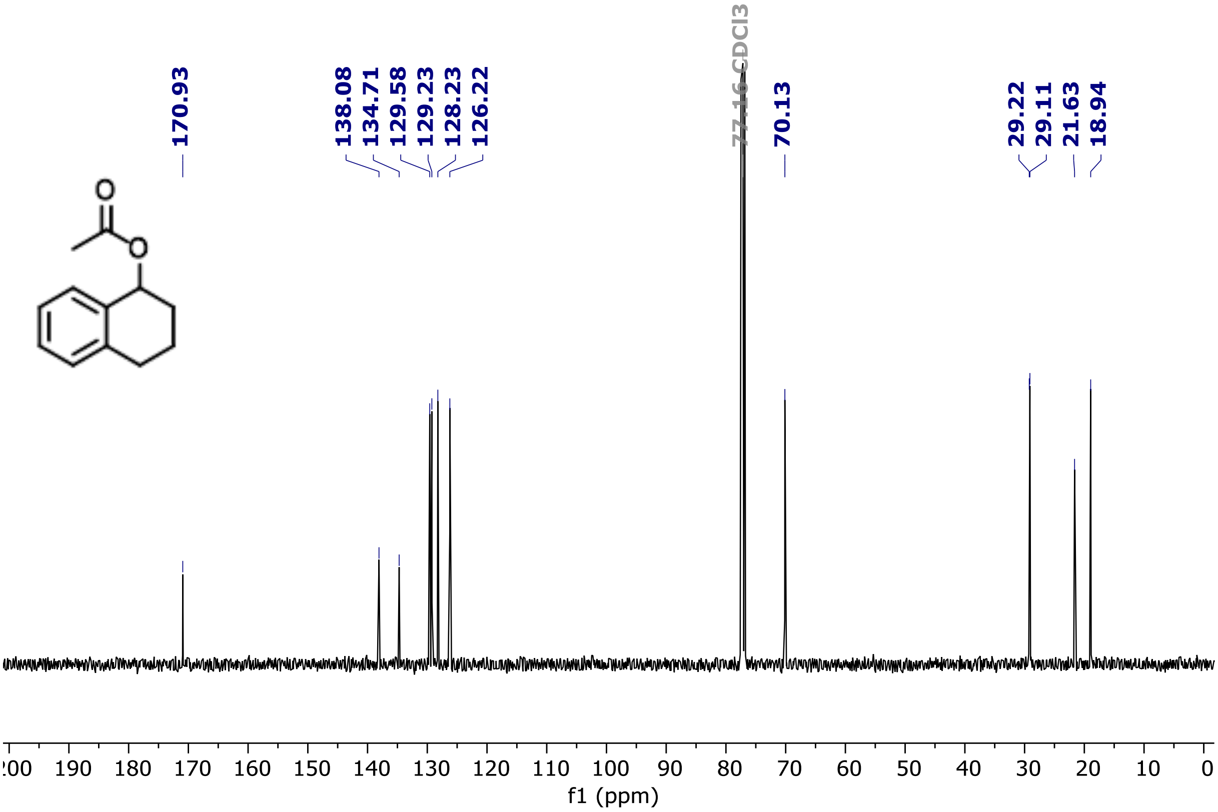

Methyl 3-acetoxy-3-phenylpropanoate (**2i**)

^{1}H NMR (400 MHz, $CDCl_3$)

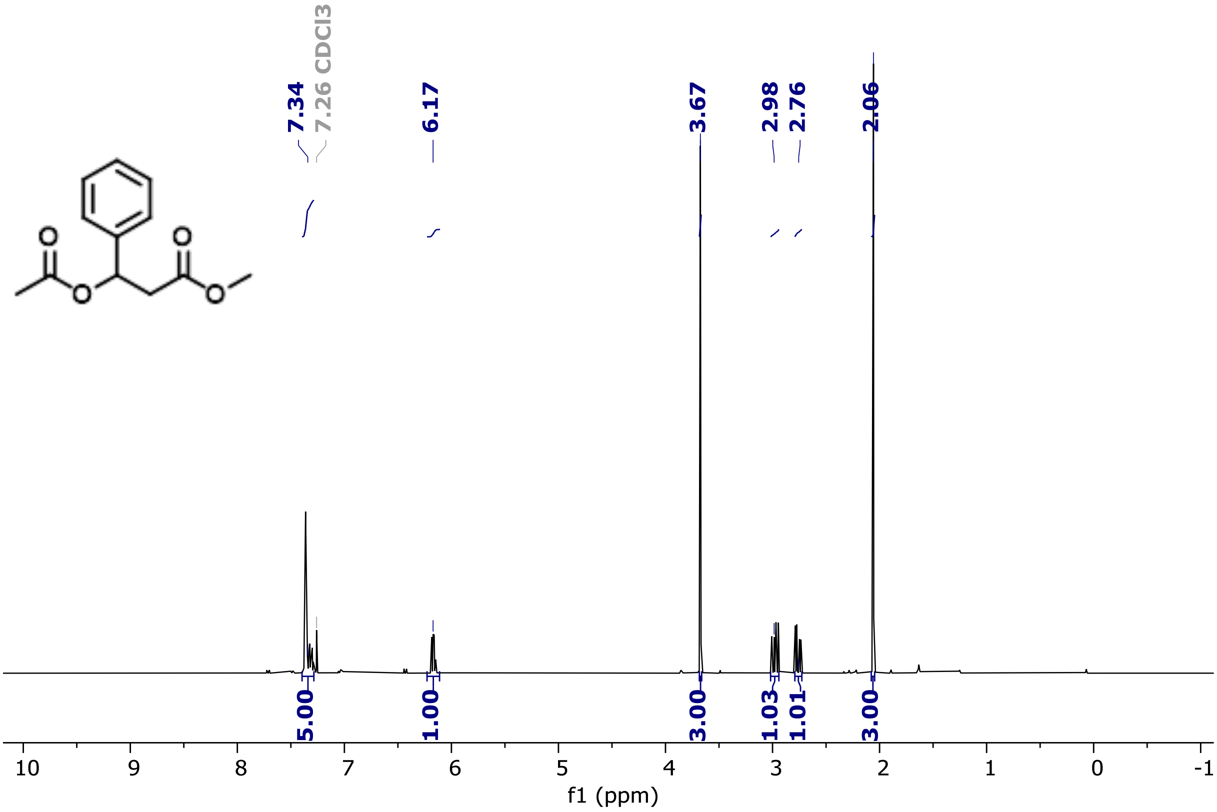

^{13}C NMR (101 MHz, $CDCl_3$)

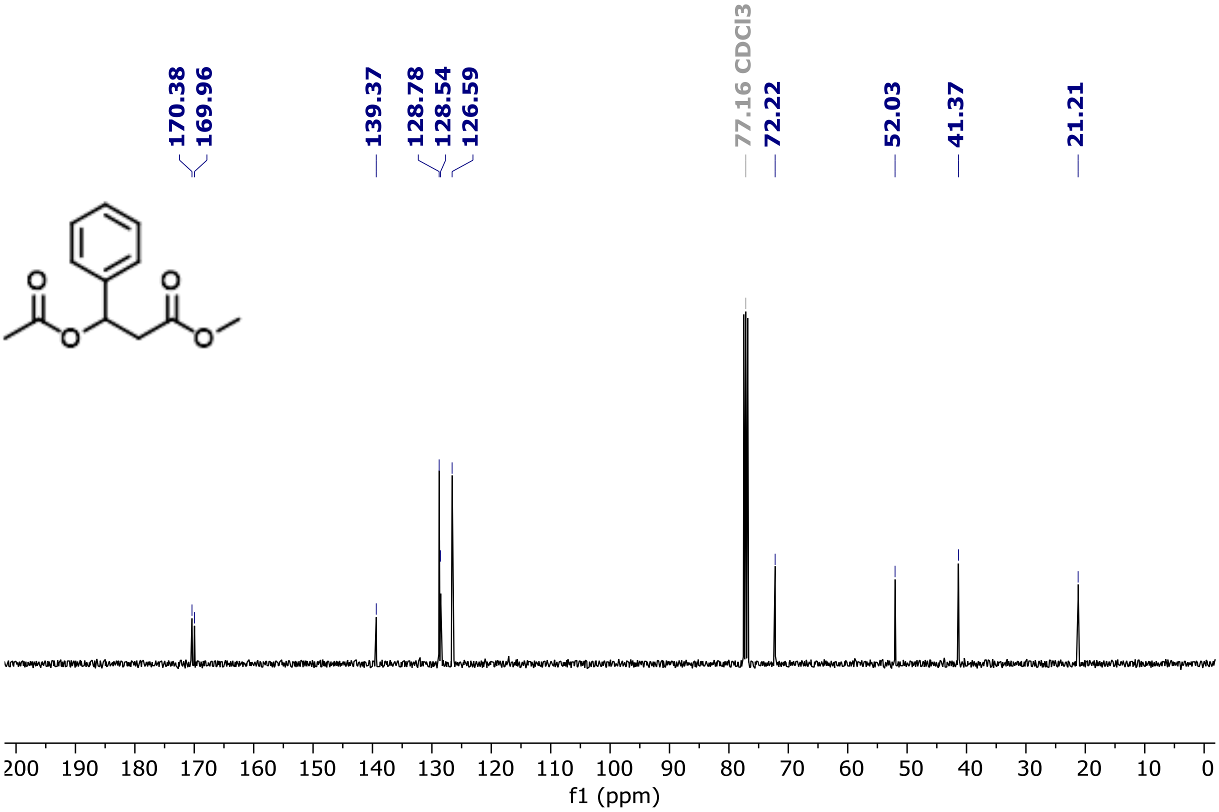

*1-Phenylpent-4-yn-1-yl acetate (**2j**)*

^{1}H NMR (400 MHz, $CDCl_3$)

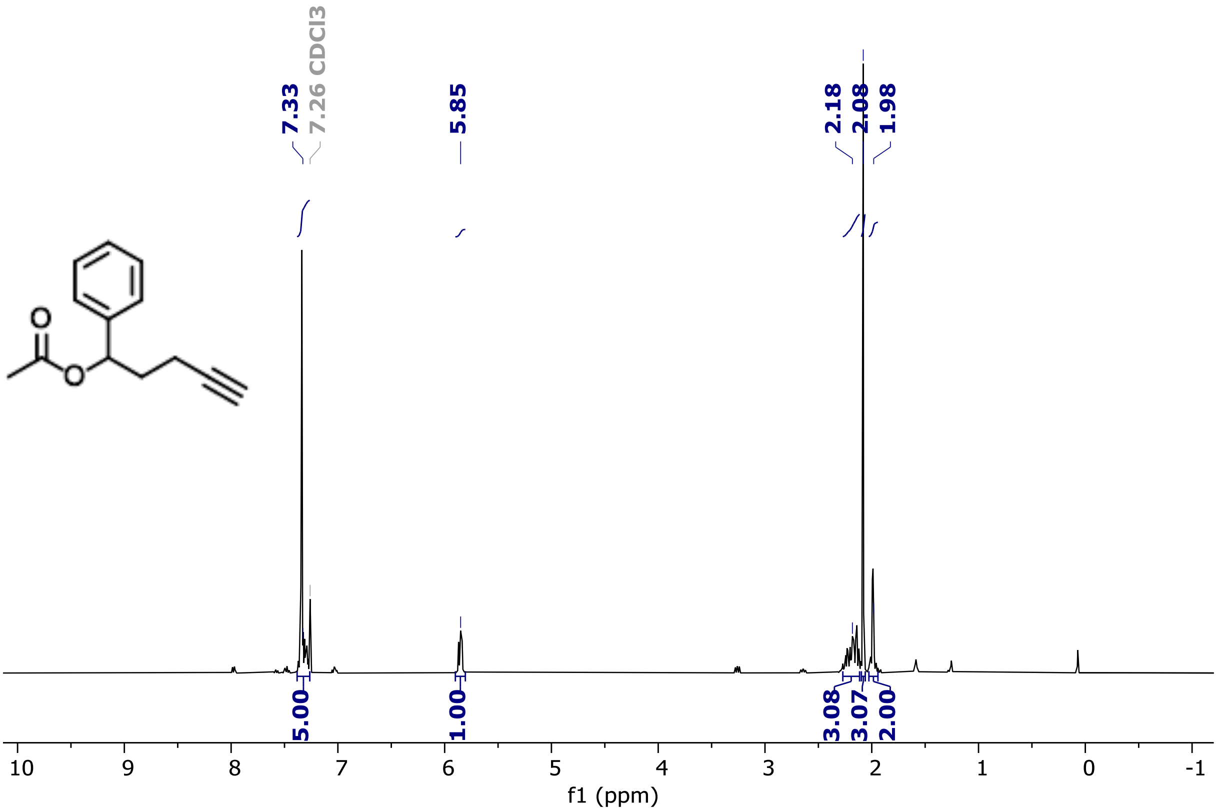

^{13}C NMR (101 MHz, $CDCl_3$)

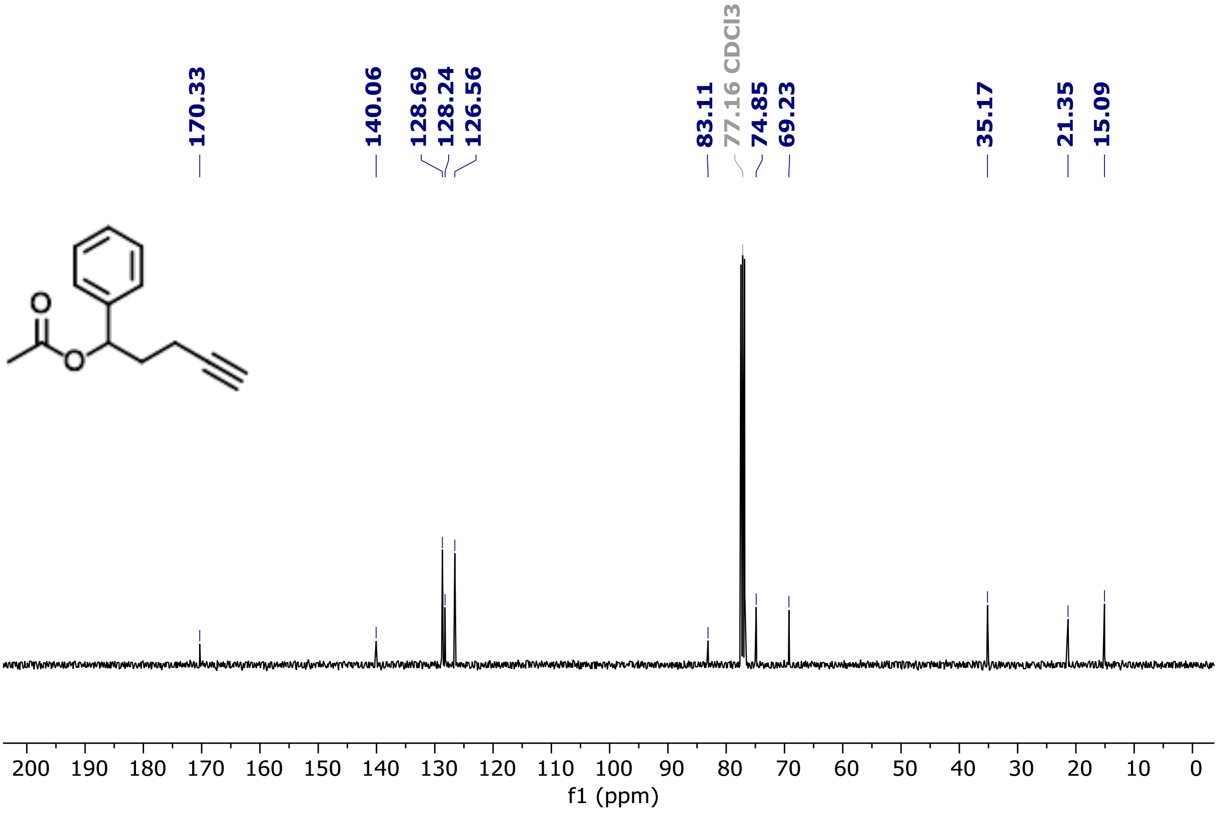

*Ethyl 2-(4-(1-acetoxy-2-methylpropyl)phenyl)propanoate (**2k**)*

^{1}H NMR (400 MHz, $CDCl_3$)

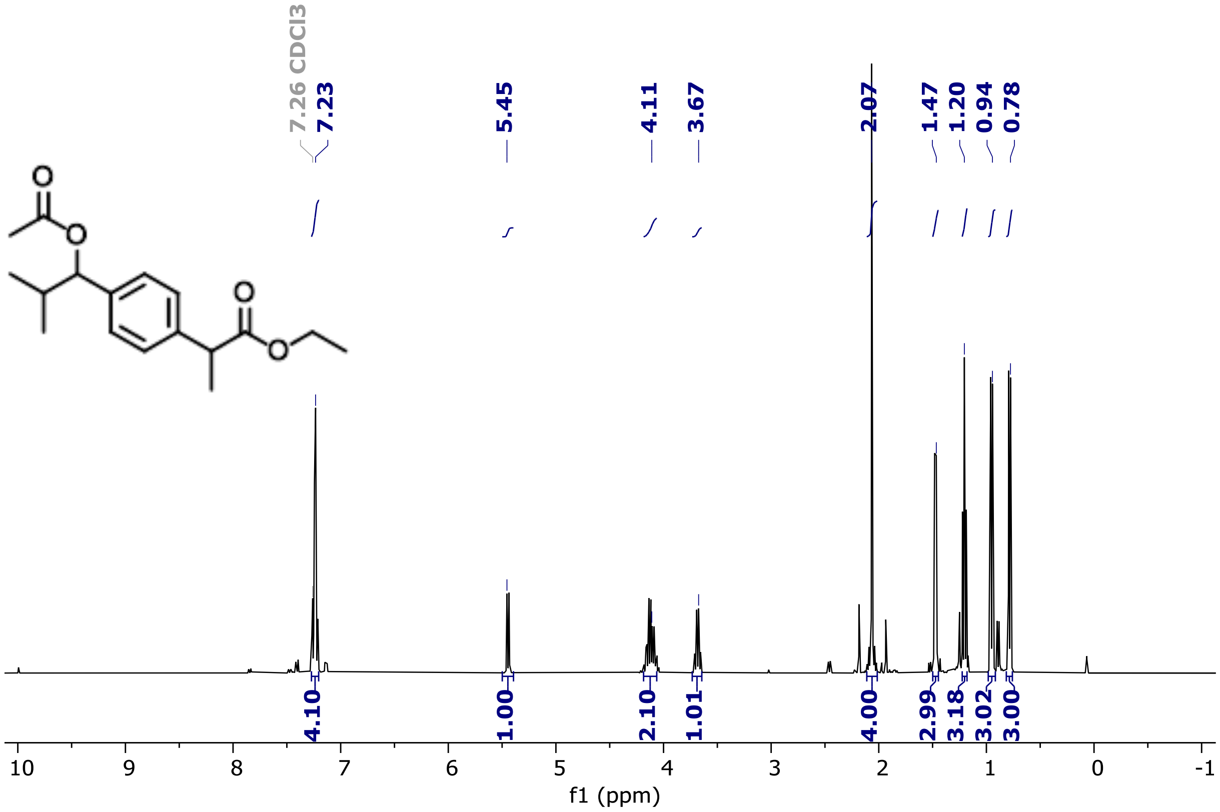

^{13}C NMR (101 MHz, $CDCl_3$)

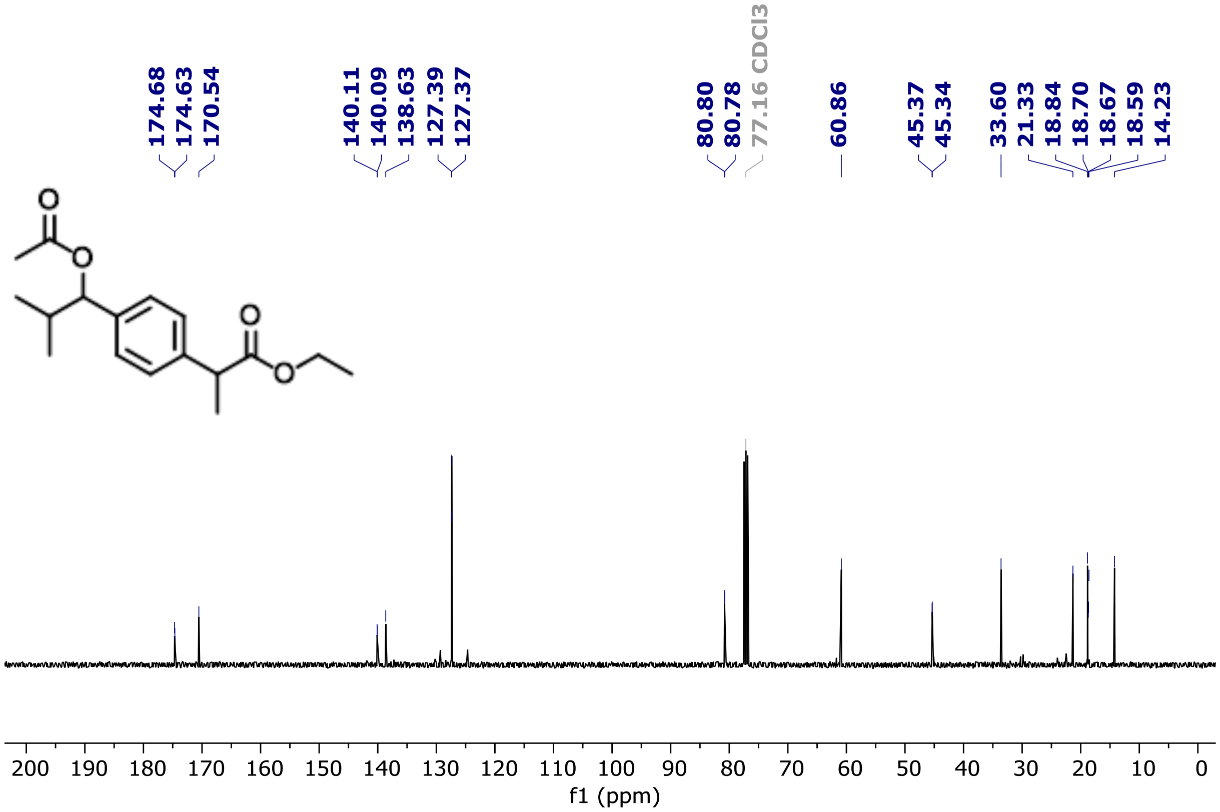

*7-Acetyl-5-(tert-butyl)-3,3-dimethyl-2,3-dihydro-1H-inden-1-yl acetate (**2l**)*

^{1}H NMR (400 MHz, $CDCl_3$)

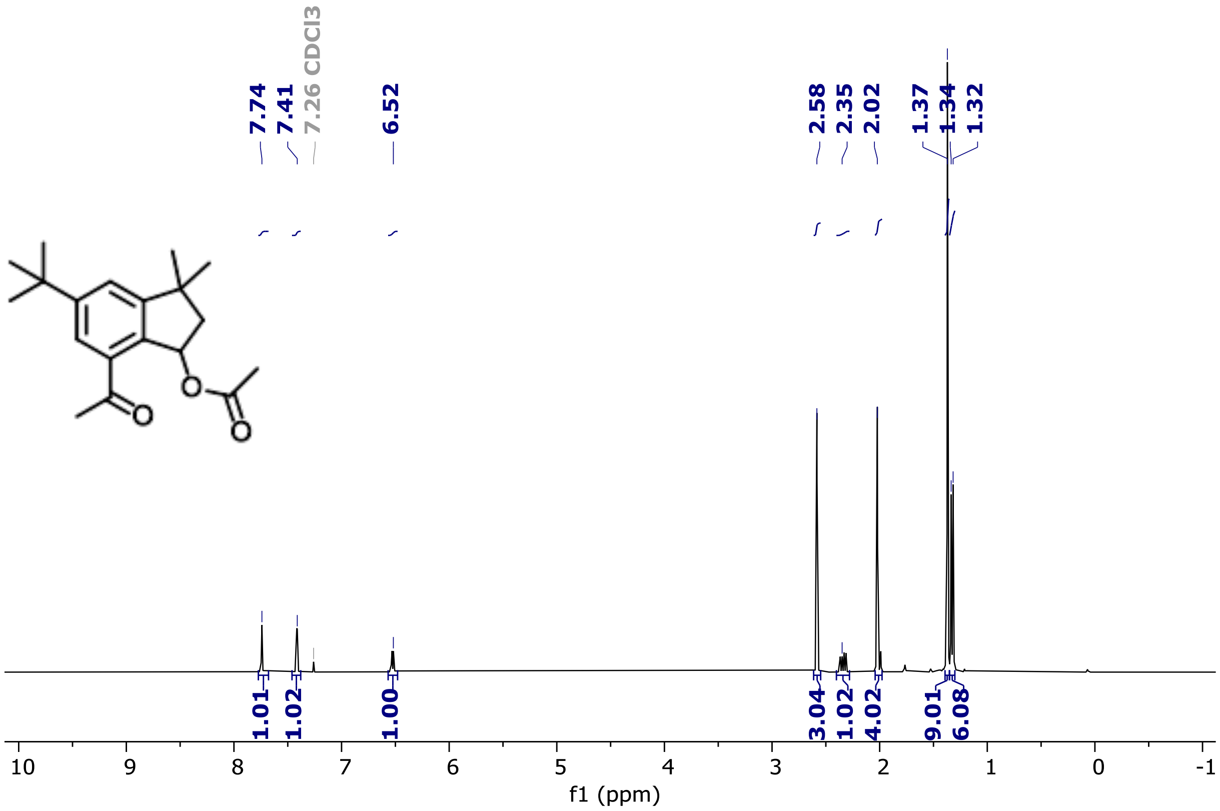

^{13}C NMR (101 MHz, $CDCl_3$)

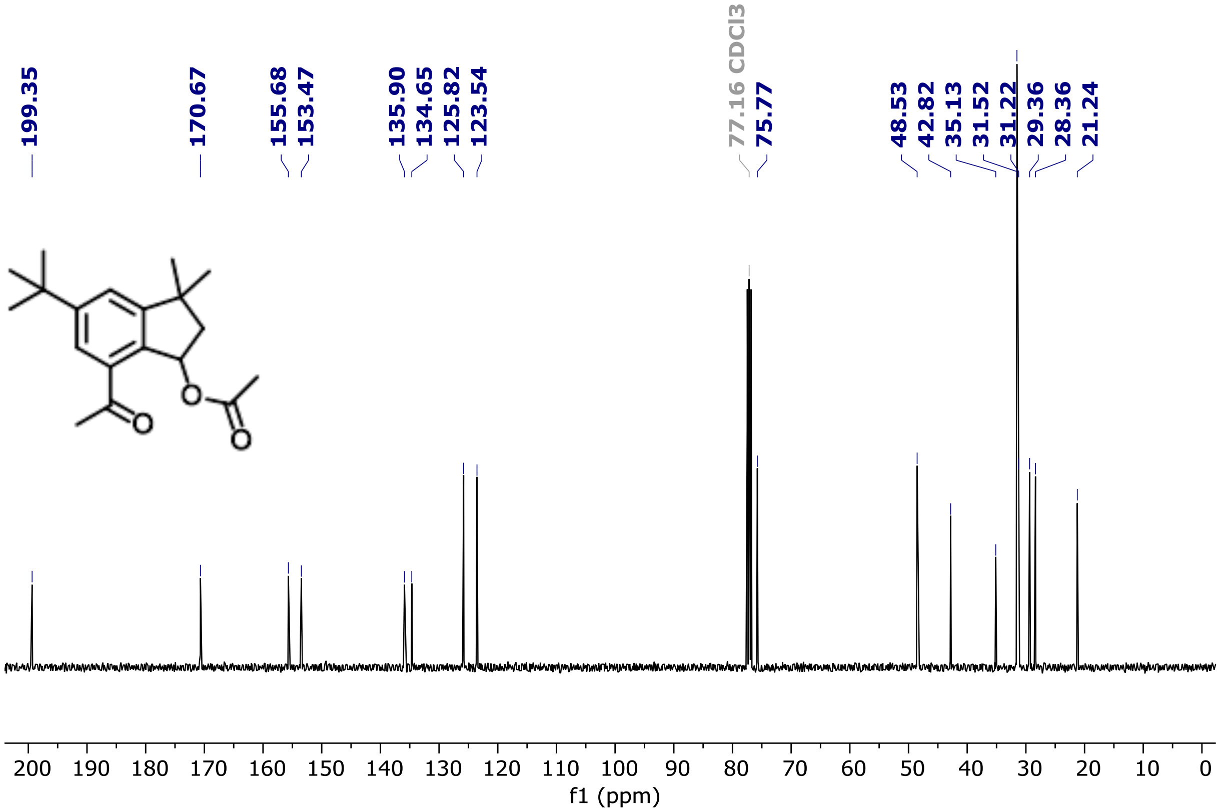

Esterification Products NMR Spectra

*1-(3-(tert-Butyl)phenyl)ethyl benzoate (**3a**)*

^{1}H NMR (400 MHz, $CDCl_3$)

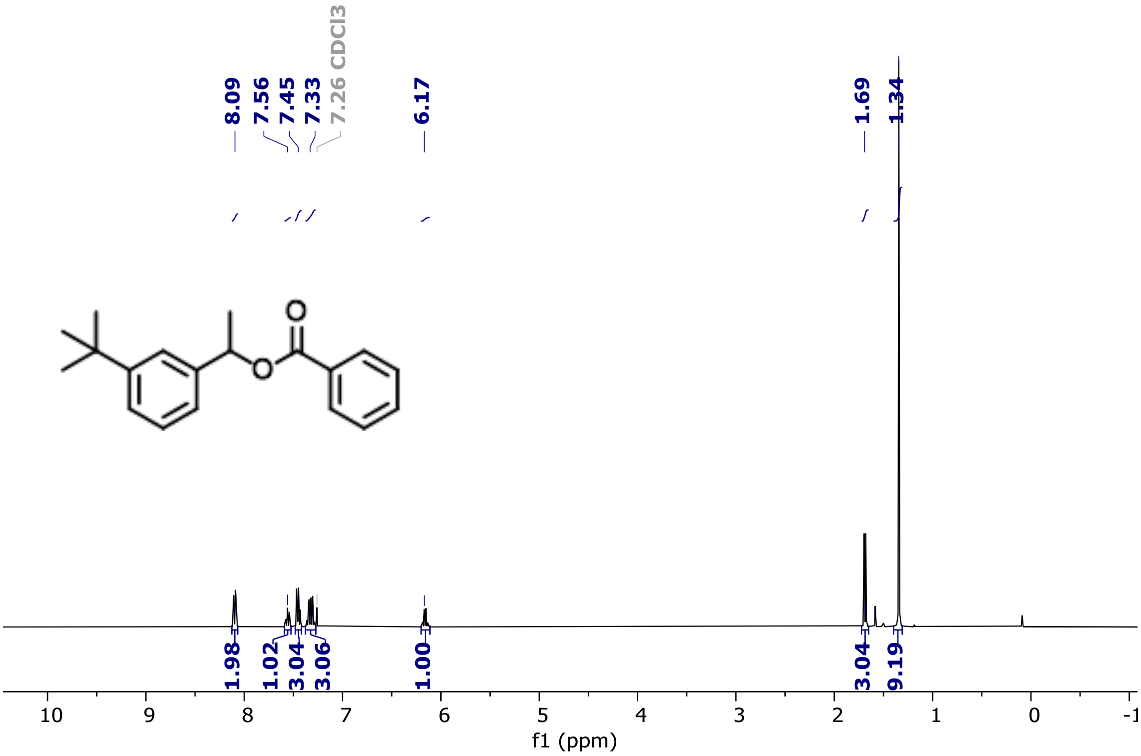

^{13}C NMR (101 MHz, $CDCl_3$)

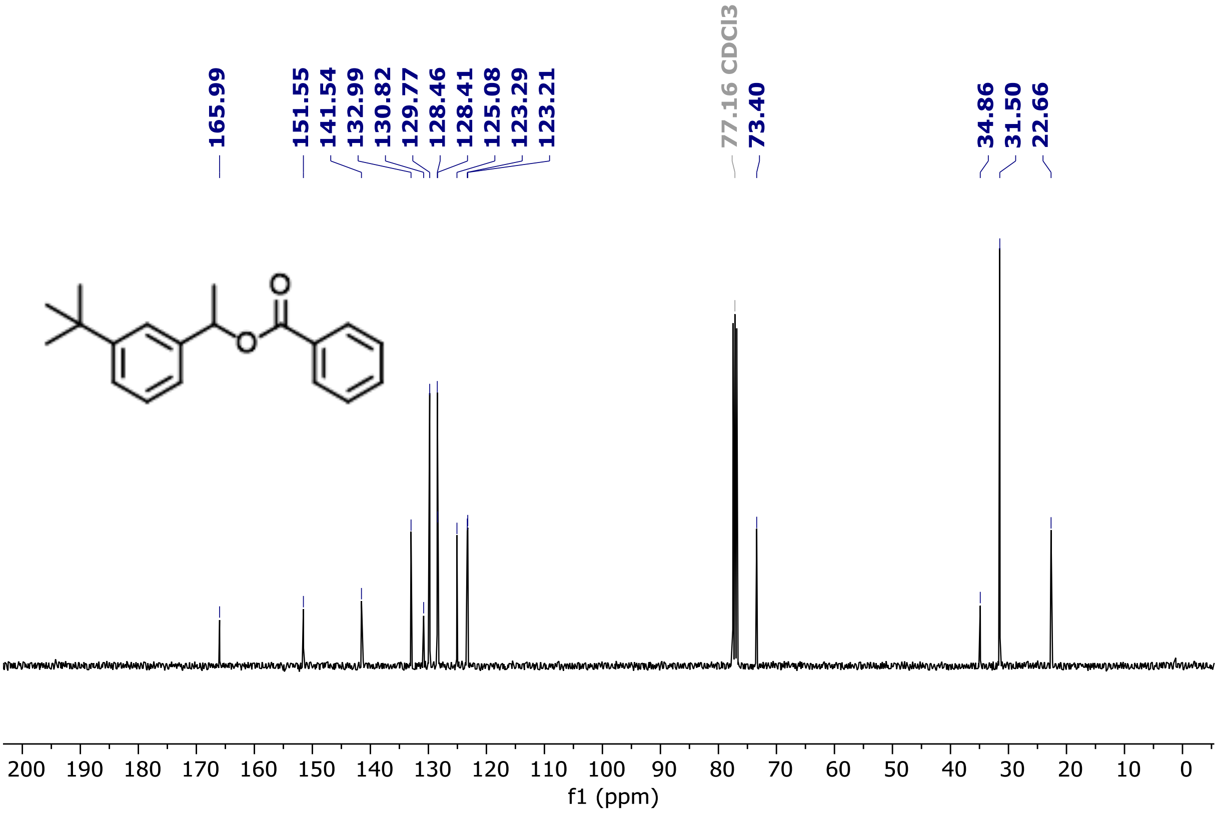

*1-(3-(tert-Butyl)phenyl)ethyl 4-fluorobenzoate (**3b**)*

^{1}H NMR (400 MHz, $CDCl_3$)

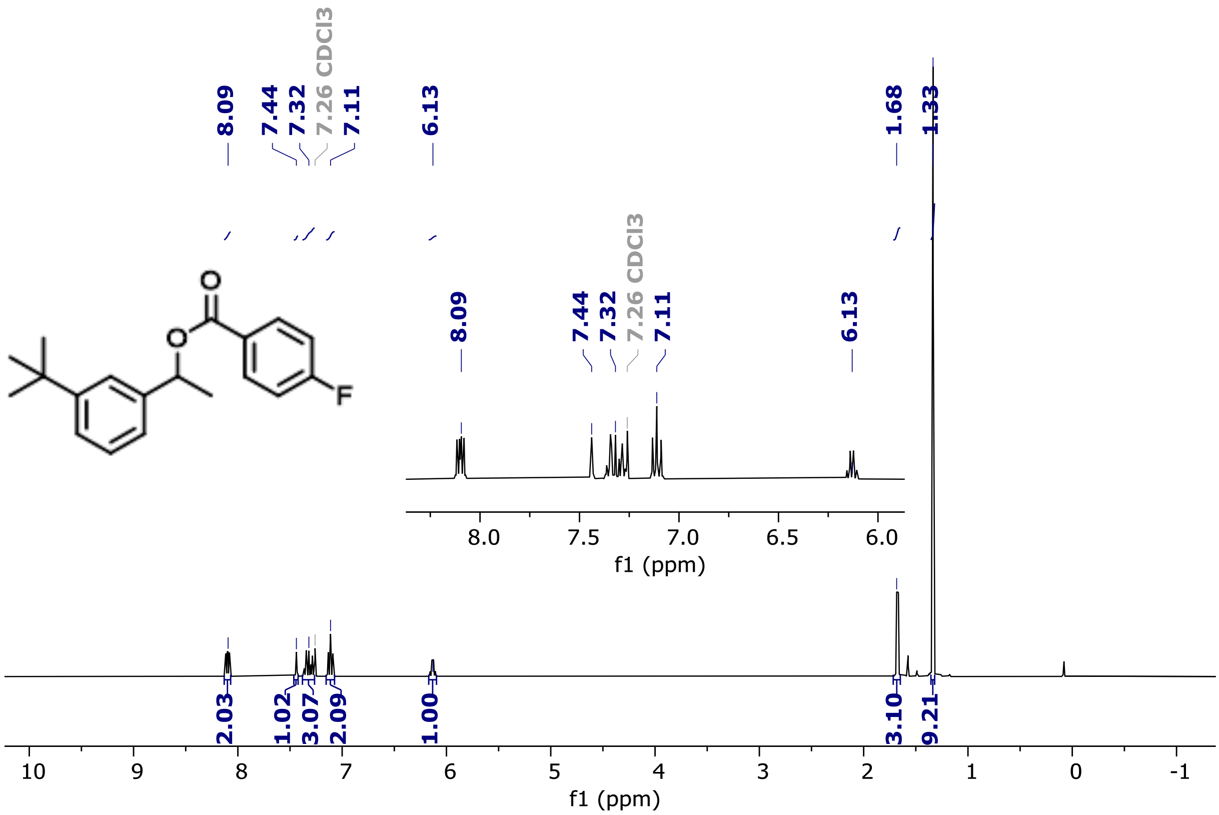

^{13}C NMR (101 MHz, $CDCl_3$)

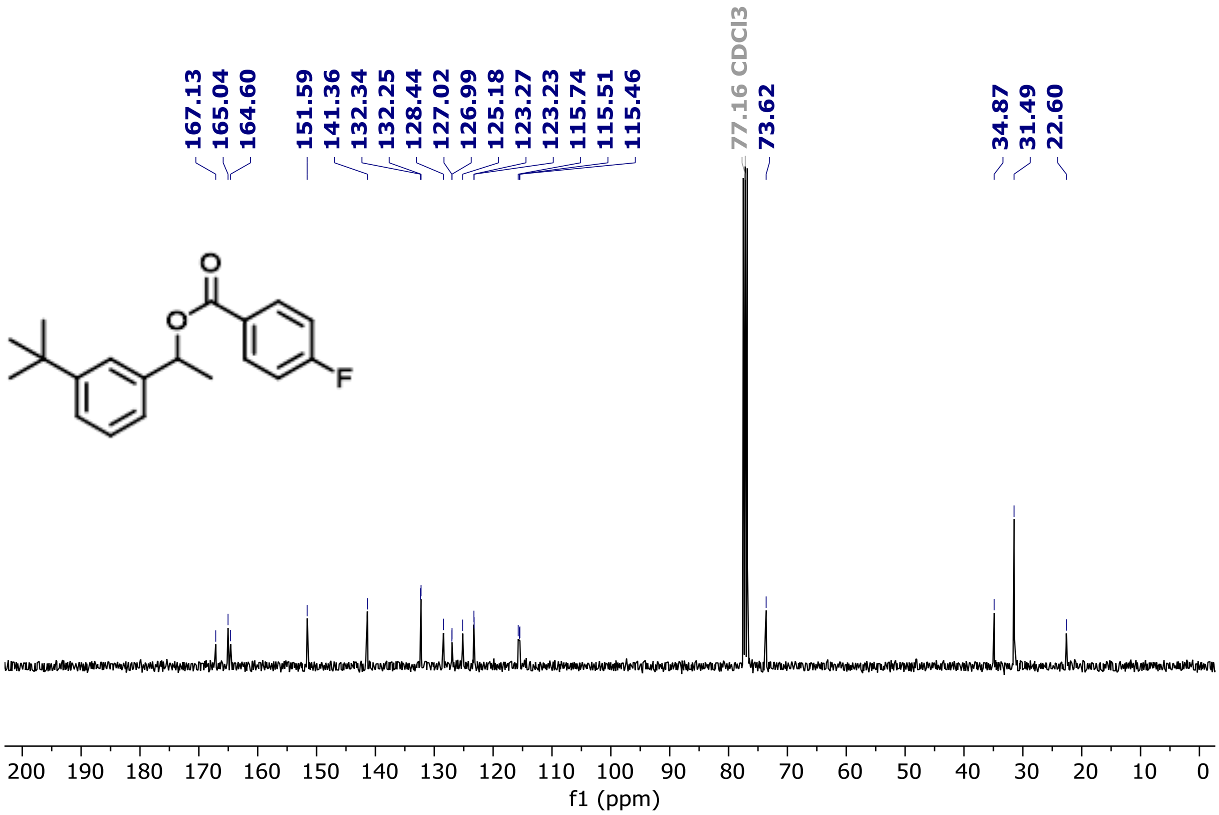

^{19}F NMR (376 MHz, $CDCl_3$)

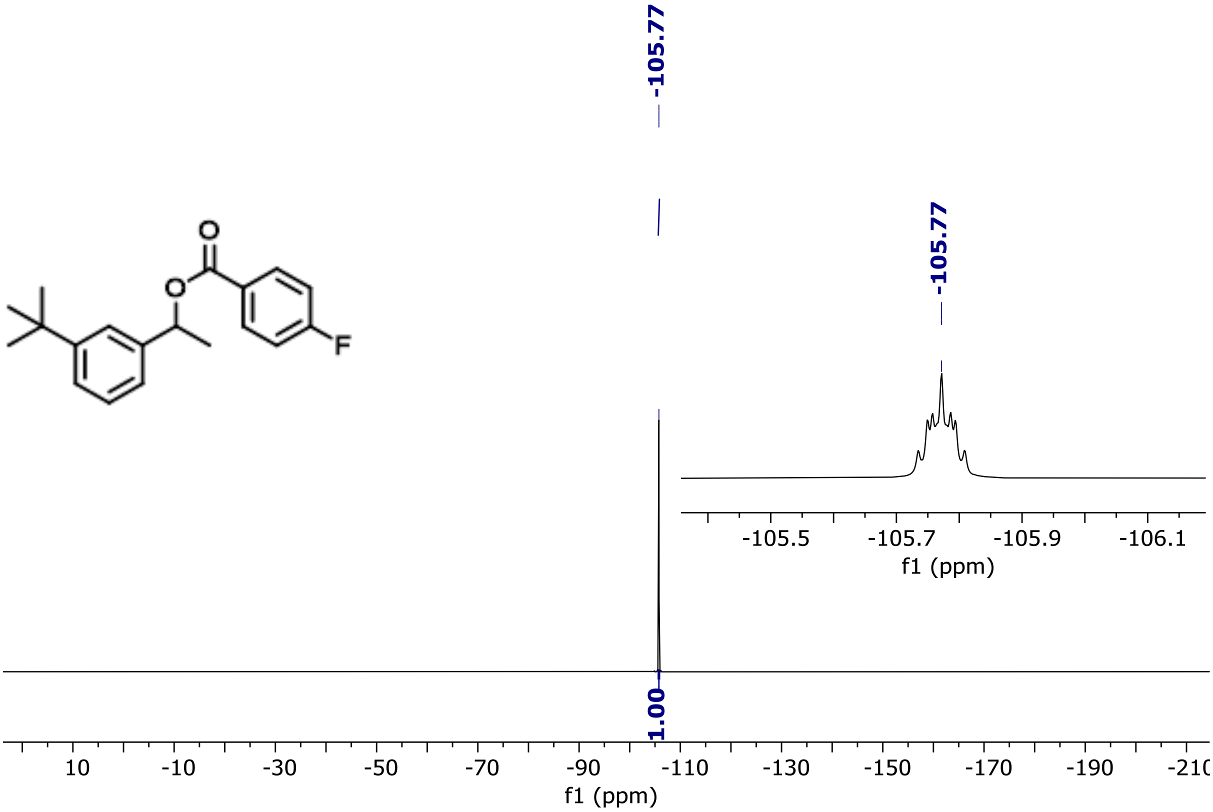

*1-(3-(tert-Butyl)phenyl)ethyl propionate (**3c**)*

^{1}**H NMR** (400 MHz, $CDCl_3$)

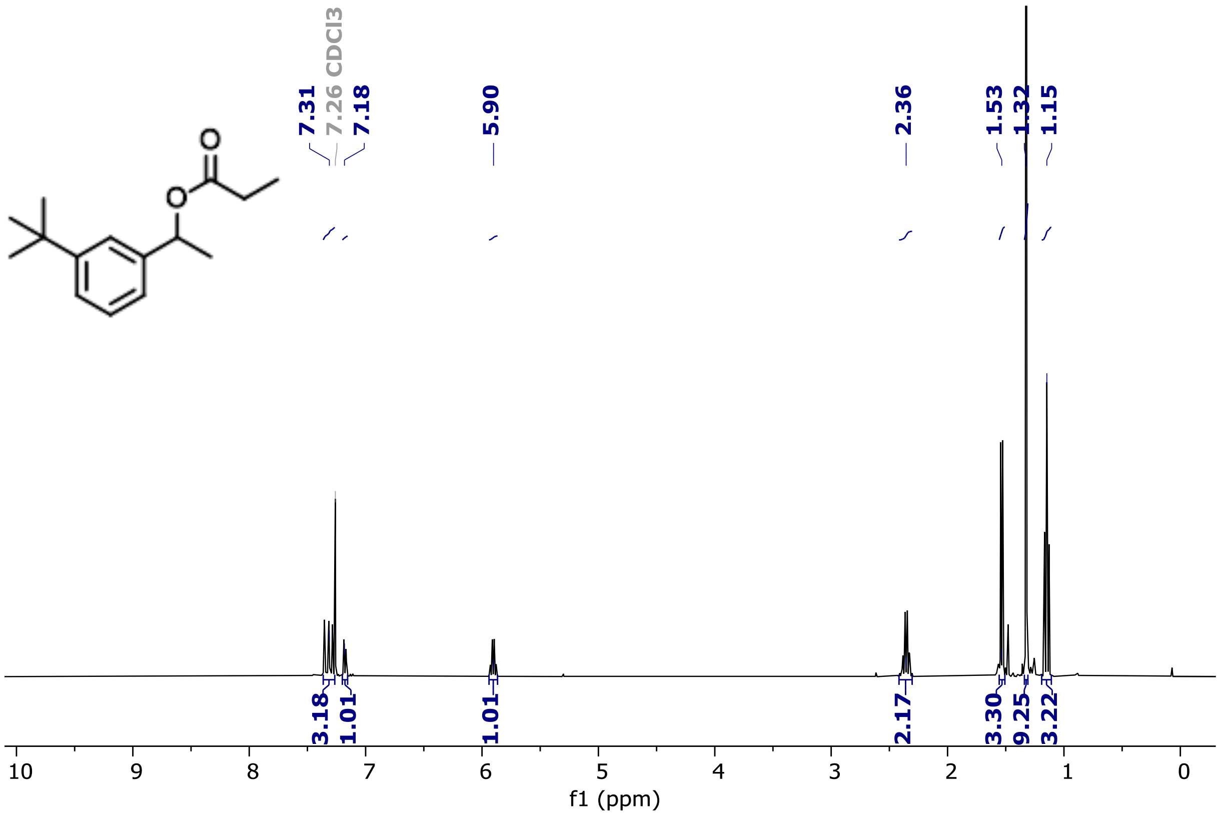

^{13}**C NMR** (101 MHz, $CDCl_3$)

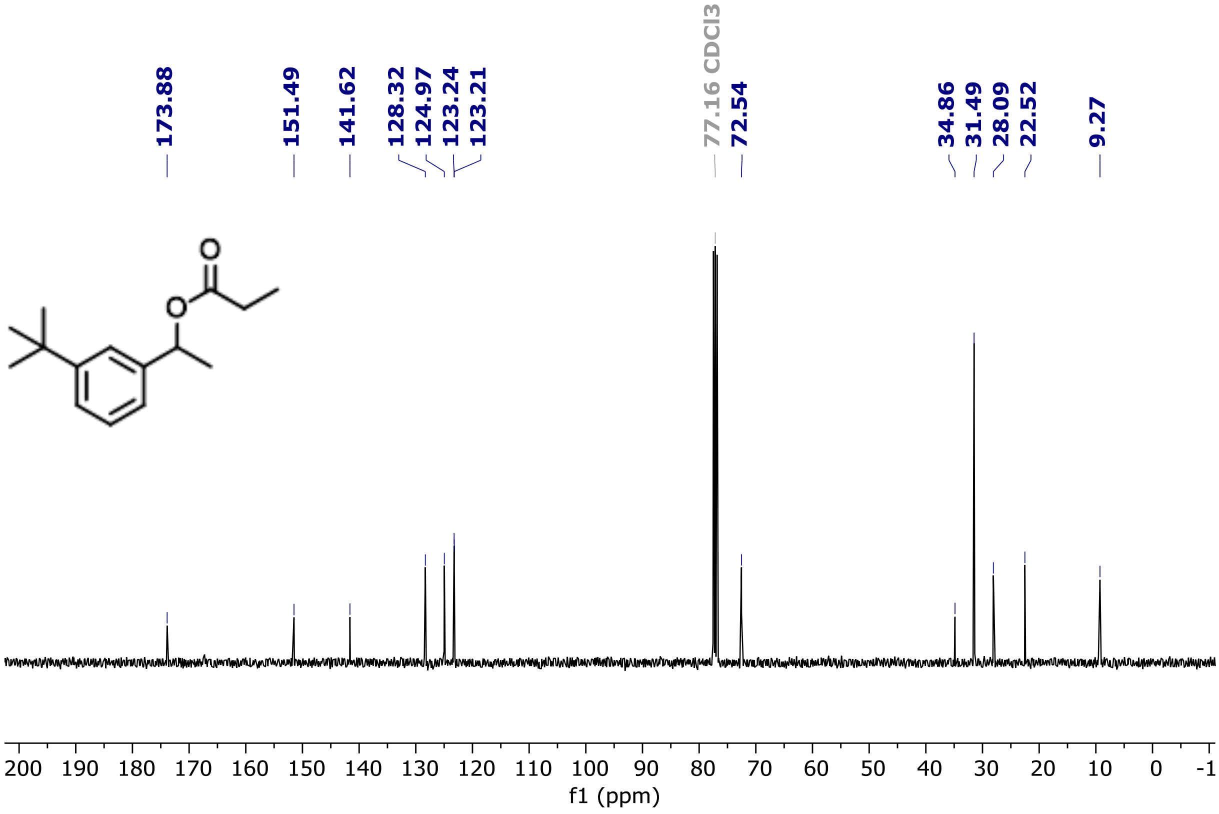

*1-(3-(tert-Butyl)phenyl)ethyl cyclopropanecarboxylate (**3d**)*

^{1}H NMR (400 MHz, $CDCl_3$)

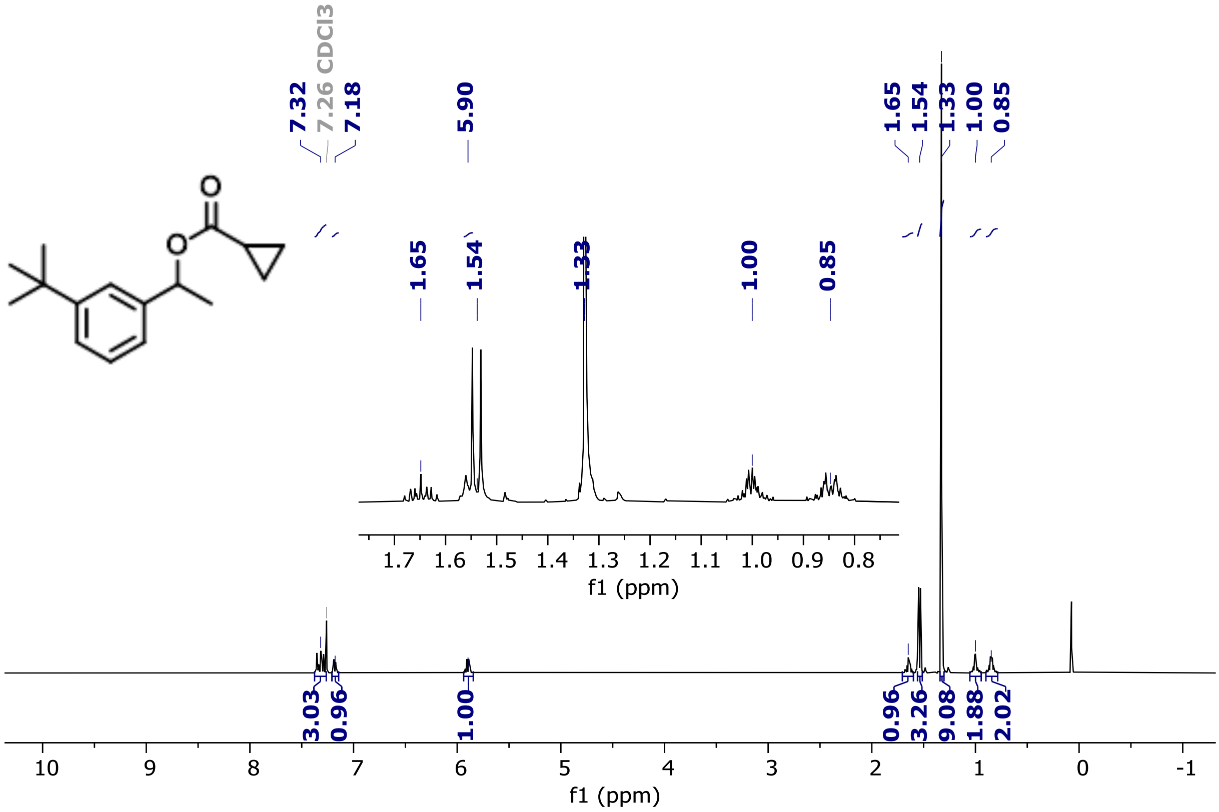

^{13}C NMR (101 MHz, $CDCl_3$)

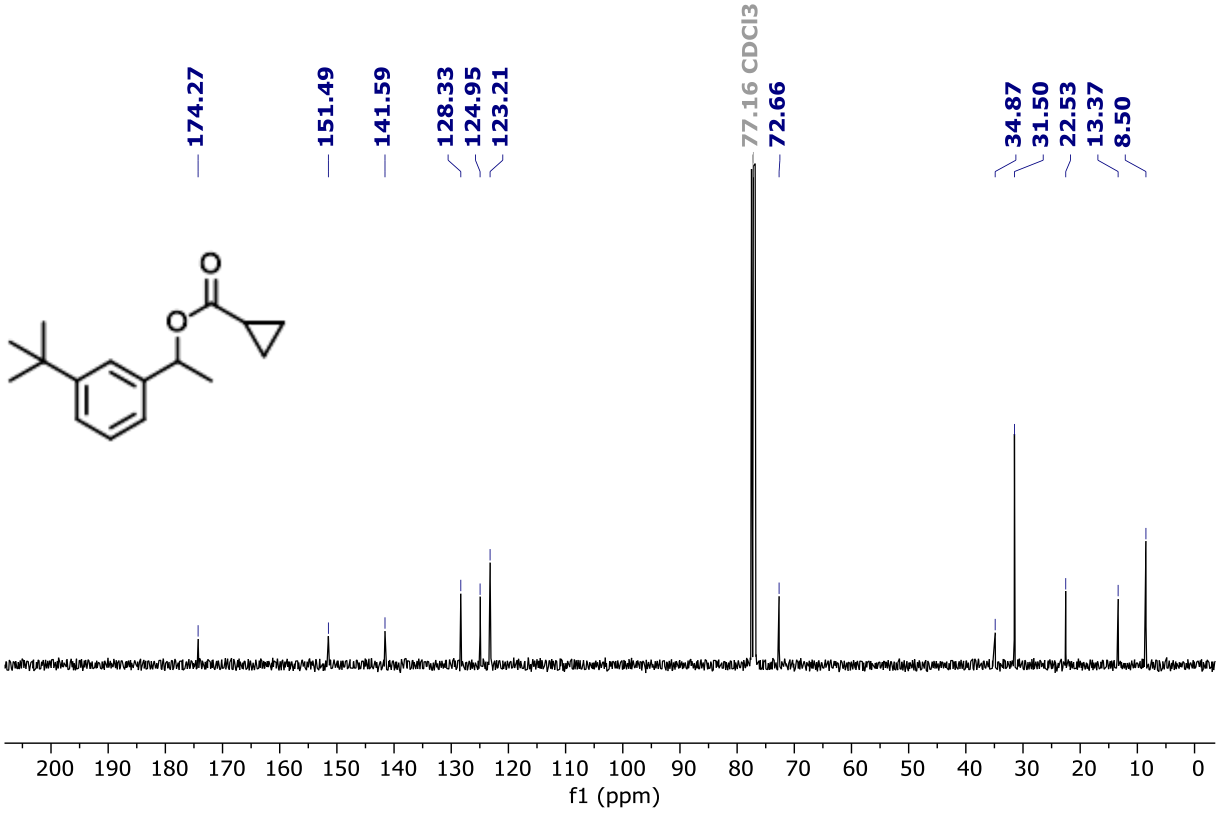

*1-(3-(tert-Butyl)phenyl)ethyl cyclobutanecarboxylate (**3e**)*

^{1}H NMR (400 MHz, $CDCl_3$)

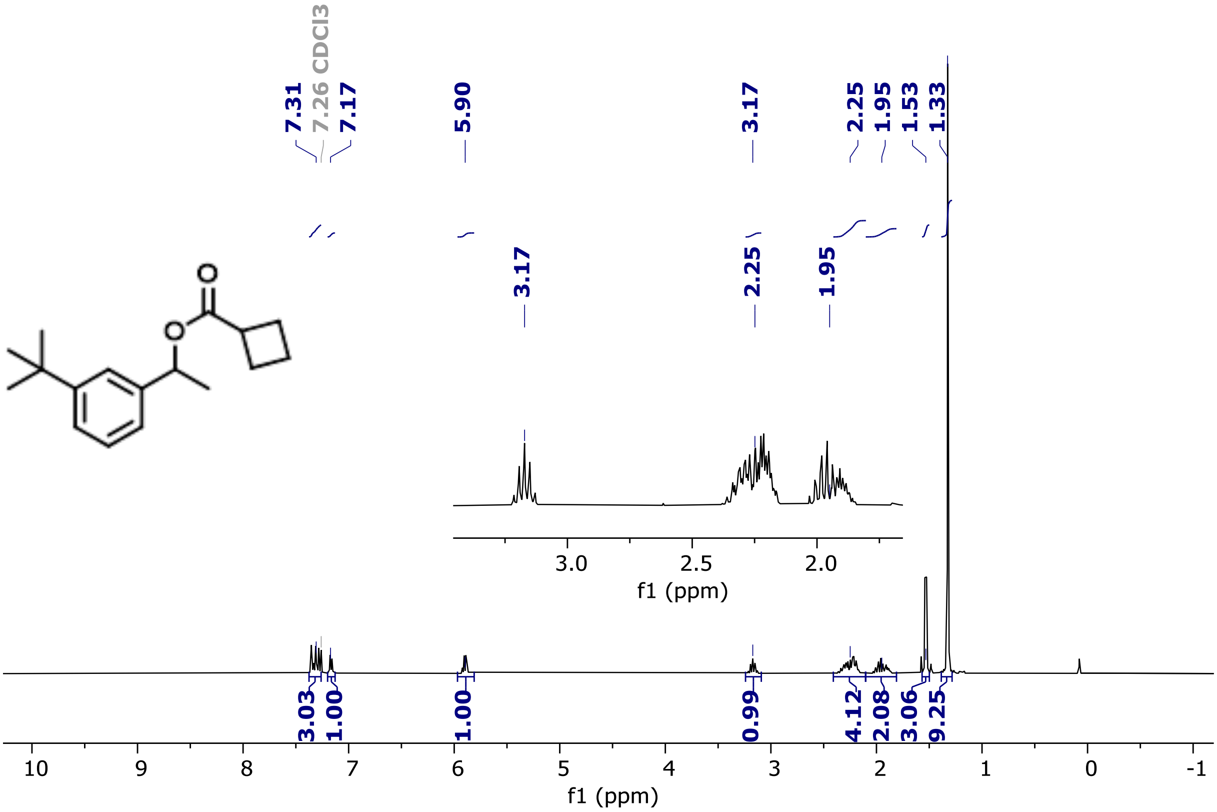

^{13}C NMR (101 MHz, $CDCl_3$)

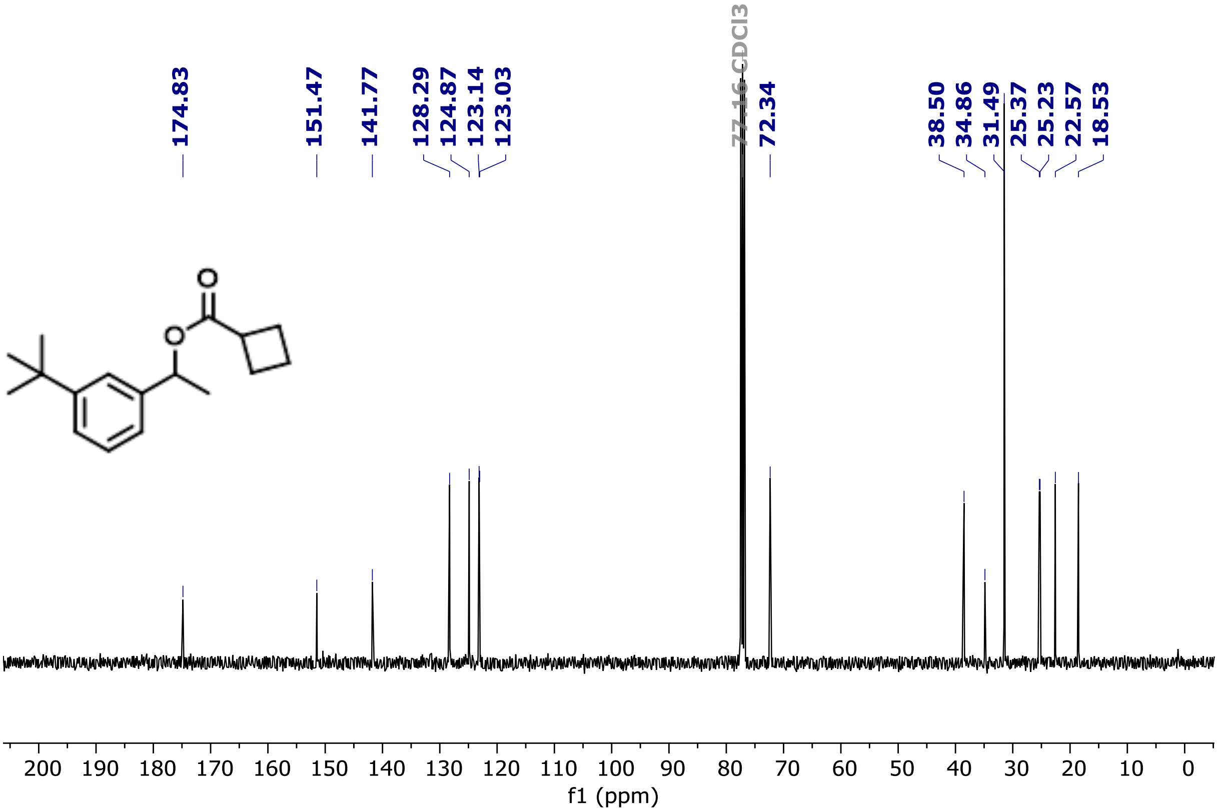

*1-(3-(tert-Butyl)phenyl)ethyl 2-cyclohexylacetate (**3f**)*

^{1}H NMR (400 MHz, $CDCl_3$)

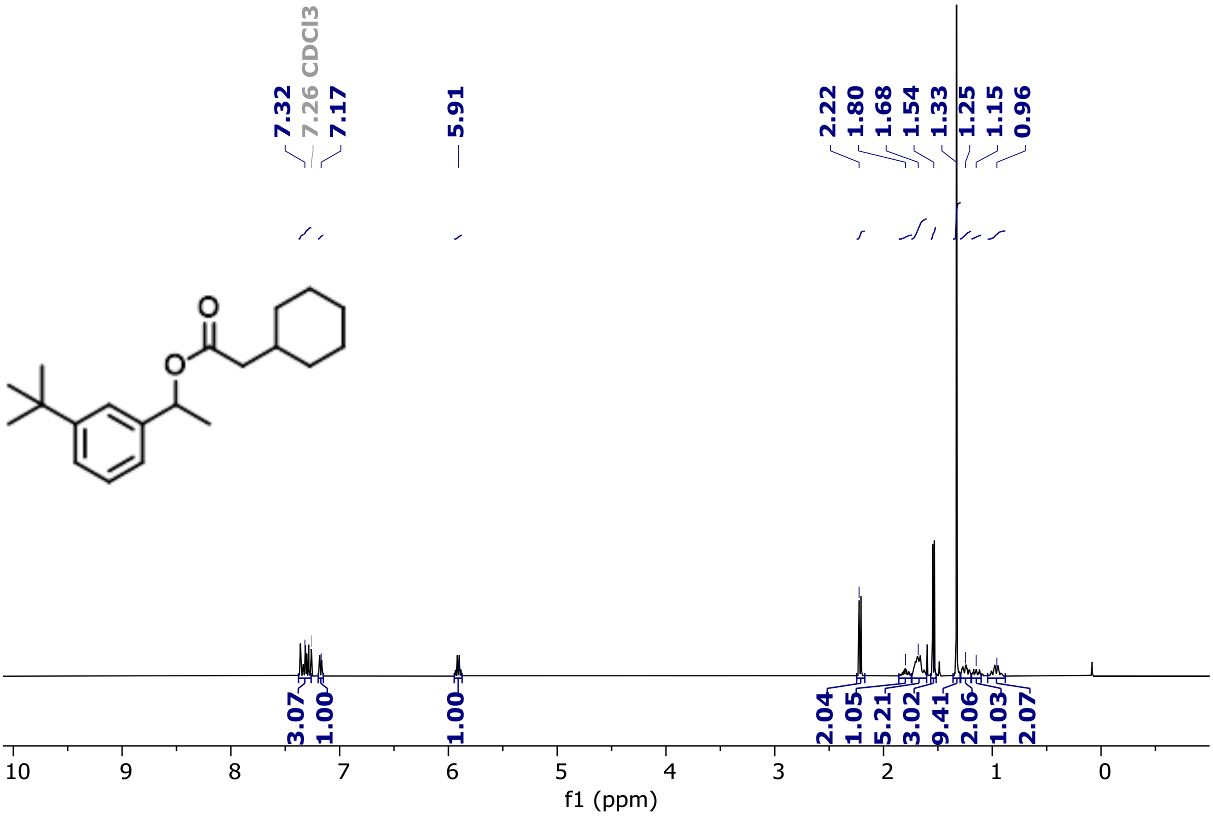

^{13}C NMR (101 MHz, $CDCl_3$)

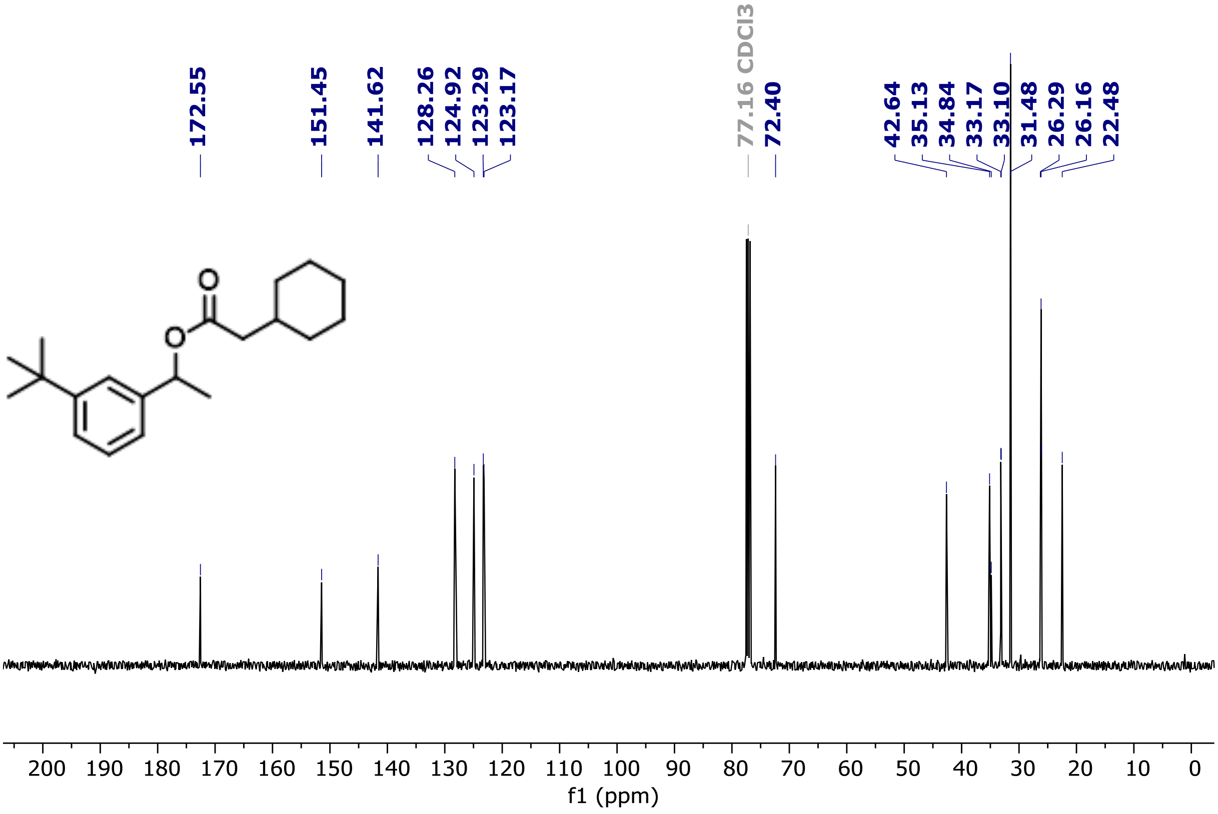

*1-(3-(tert-Butyl)phenyl)ethyl-adamantane-1-carboxylate (**3g**)*

^{1}H NMR (400 MHz, $CDCl_3$)

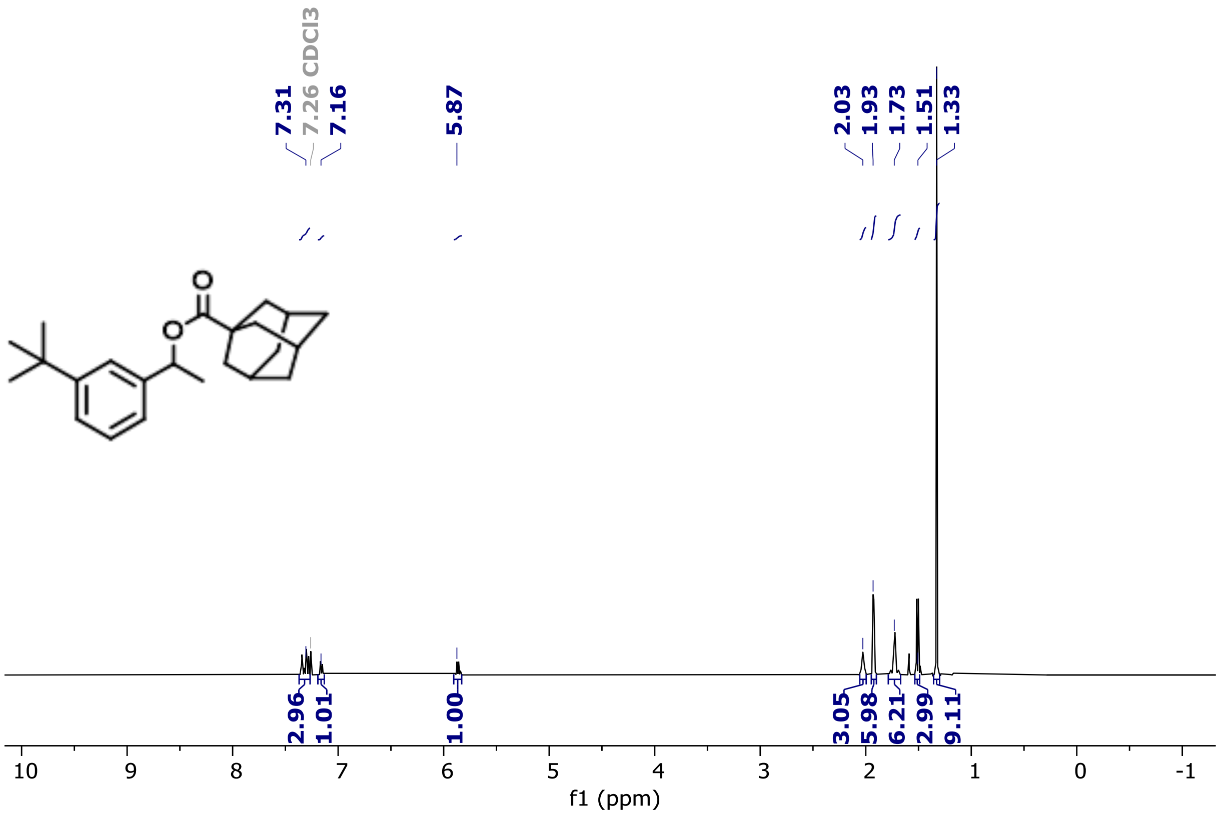

^{13}C NMR (101 MHz, $CDCl_3$)

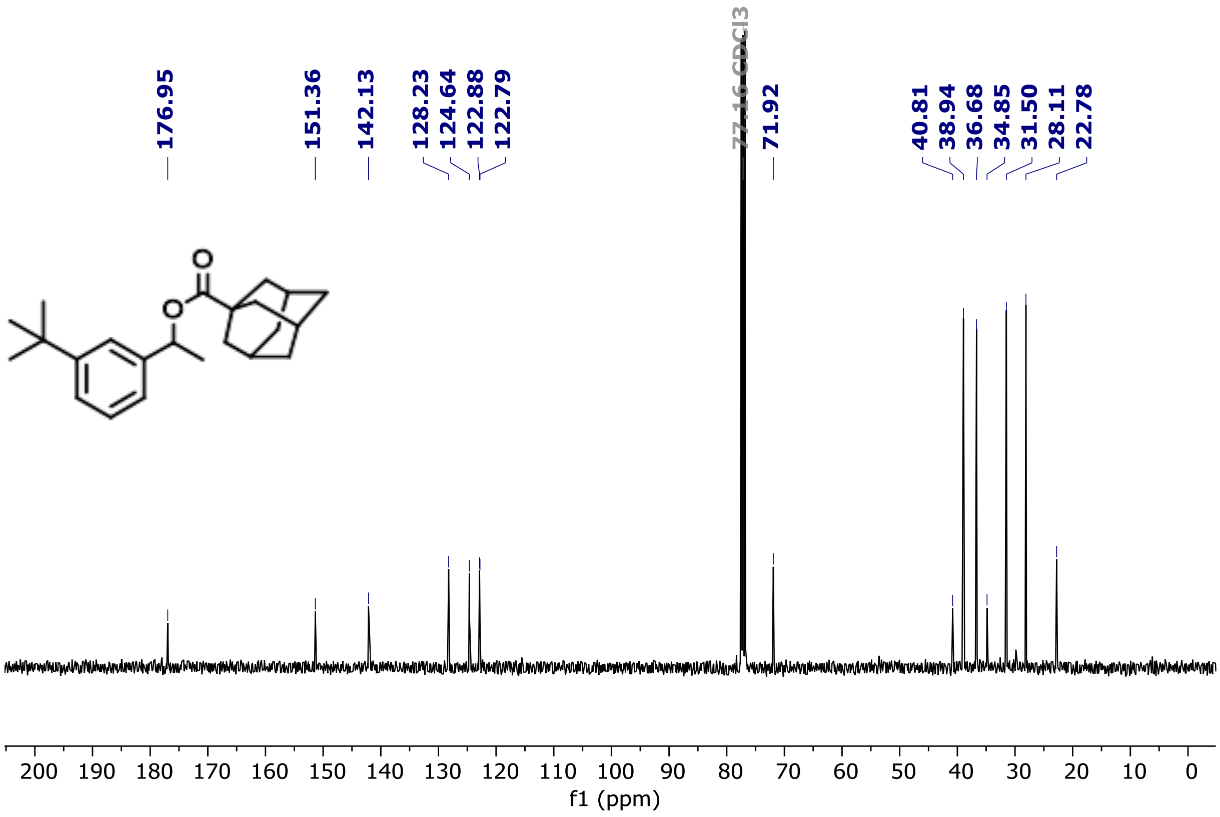

*1-(3-(tert-Butyl)phenyl)ethyl pivalate (**3h**)*

^{1}H NMR (400 MHz, $CDCl_3$)

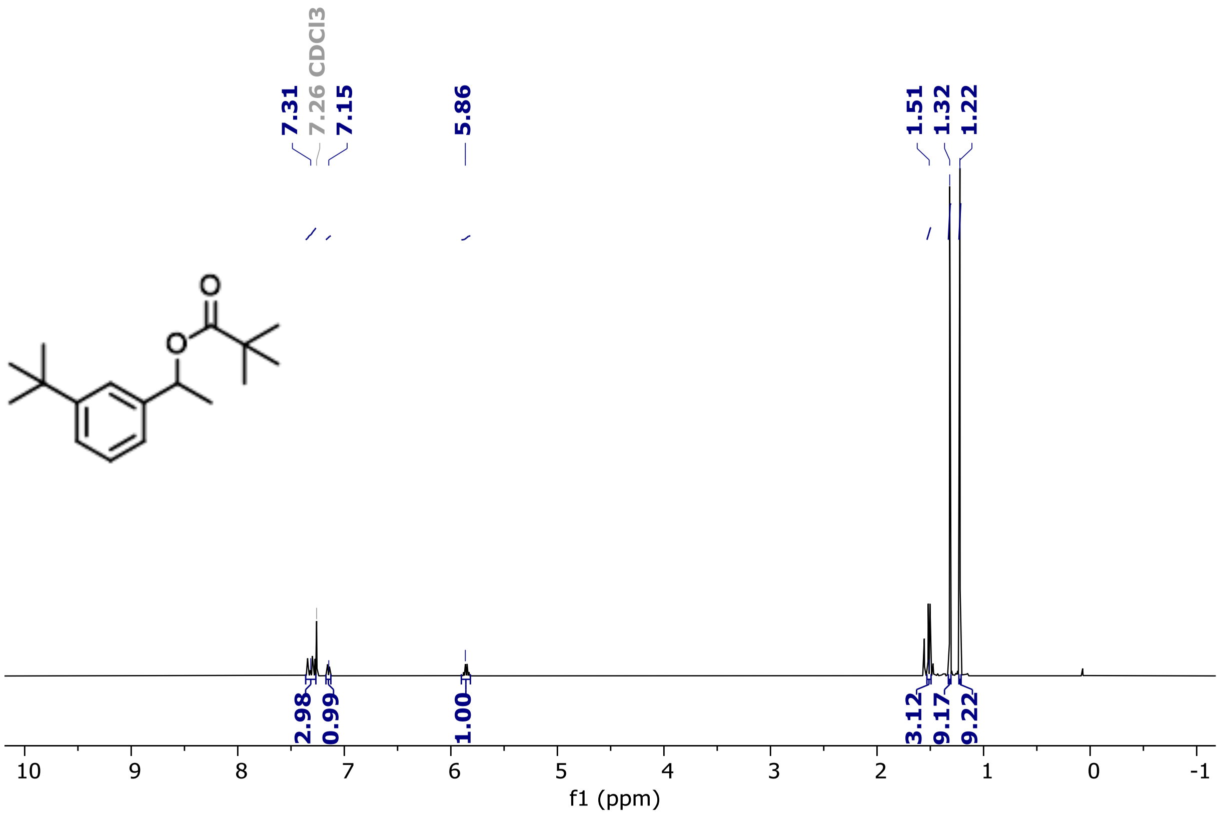

^{13}C NMR (101 MHz, $CDCl_3$)

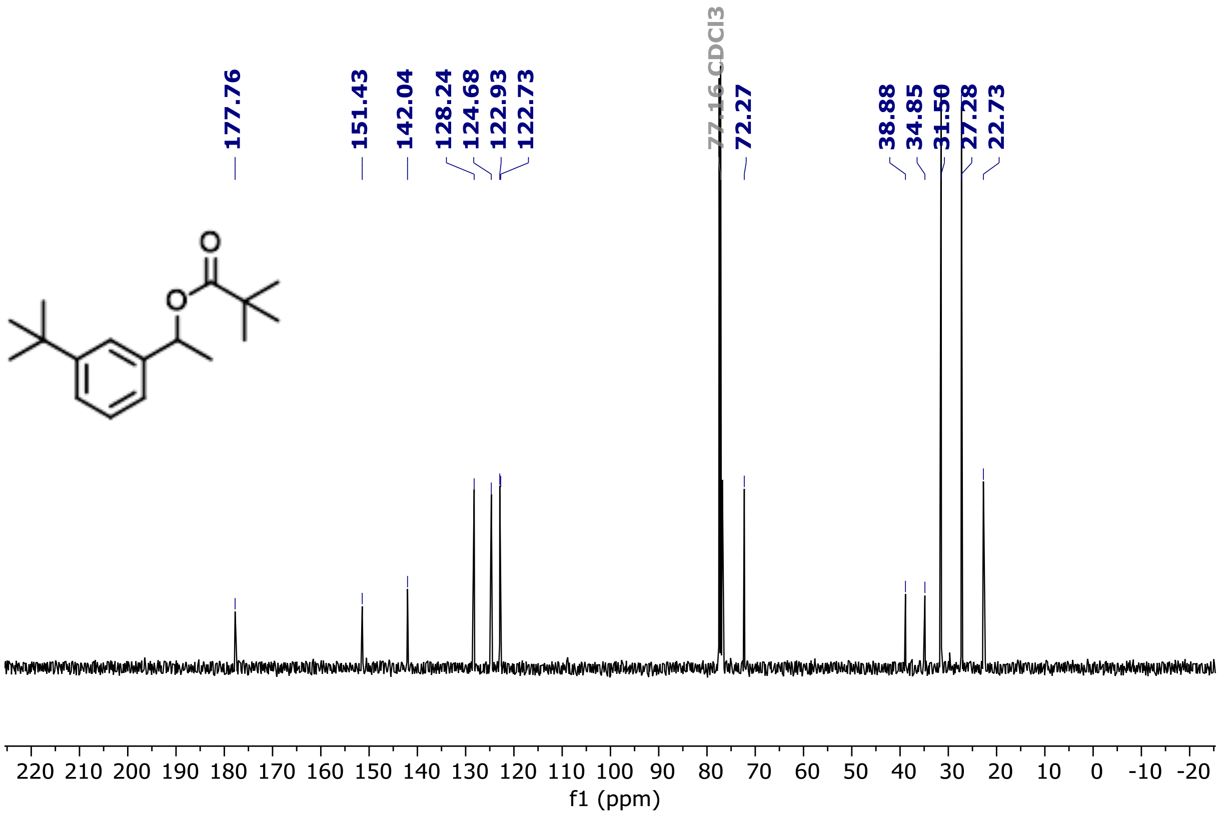

*1-(3-(tert-Butyl)phenyl)ethyl 5-bromopentanoate (**3i**)*

^{1}H NMR (400 MHz, $CDCl_3$)

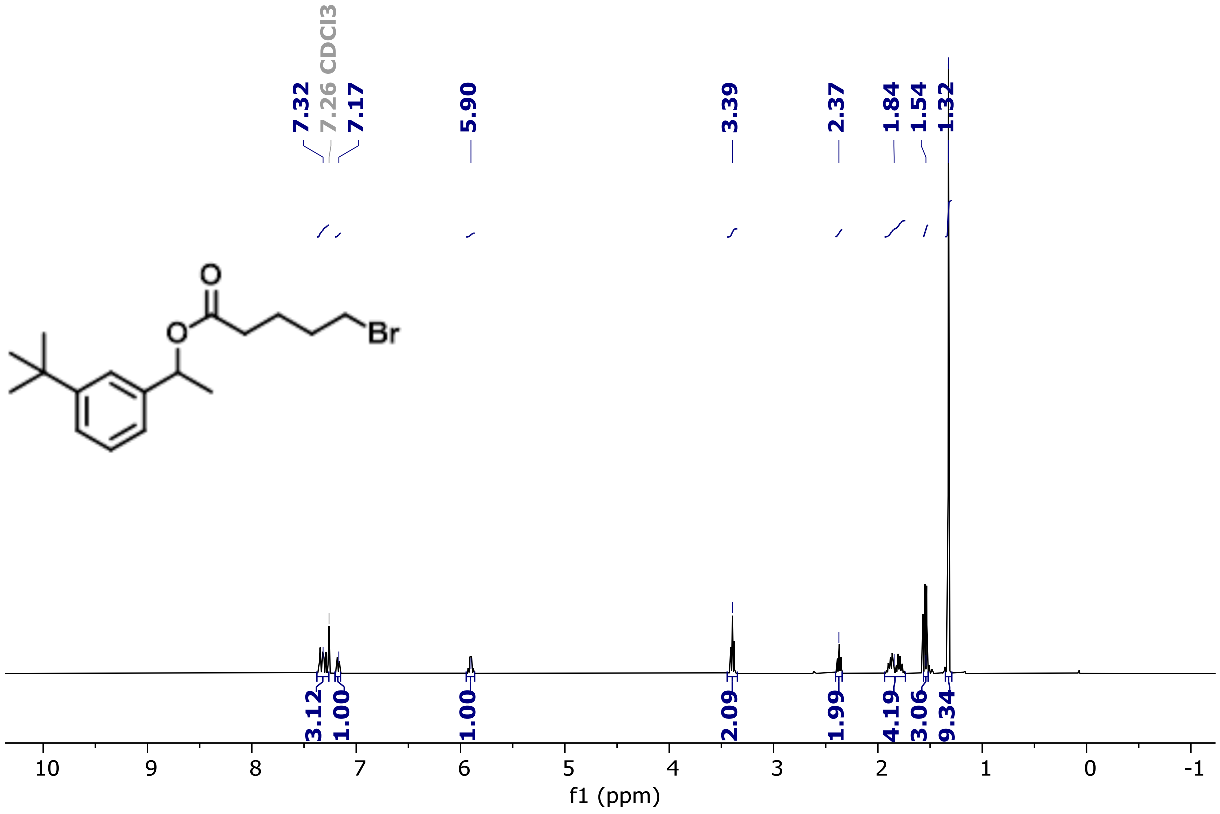

^{13}C NMR (101 MHz, $CDCl_3$)

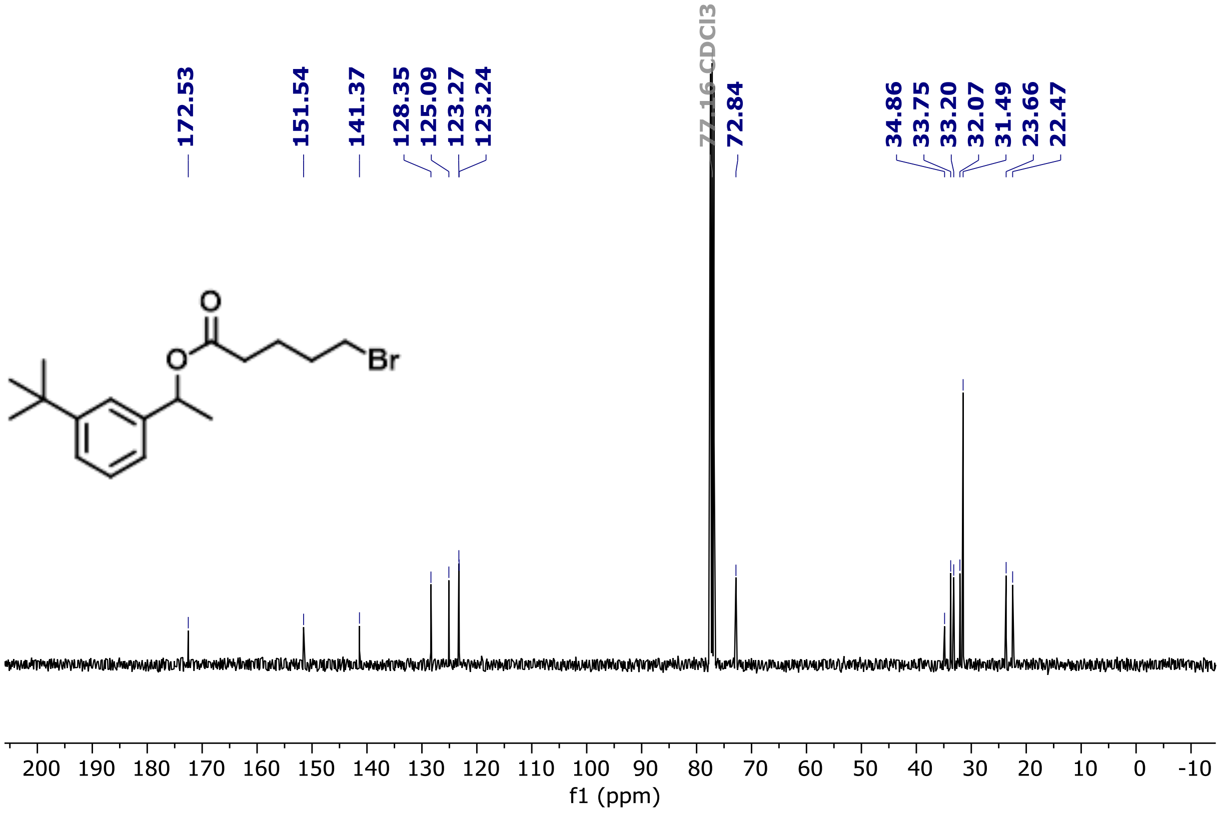

*1-(3-(tert-Butyl)phenyl)ethyl 2,2,2-trifluoroacetate (**3j**)*

^{1}H NMR (400 MHz, $CDCl_3$)

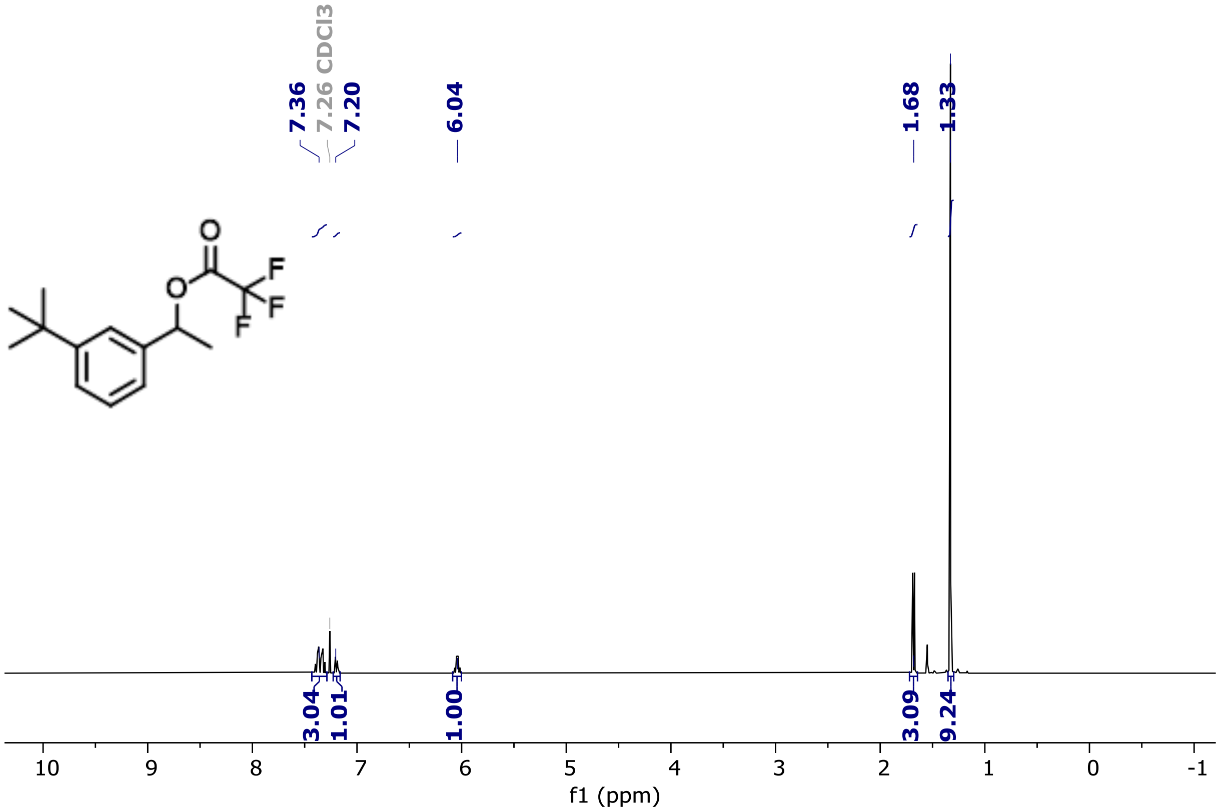

^{13}C NMR (126 MHz, $CDCl_3$)

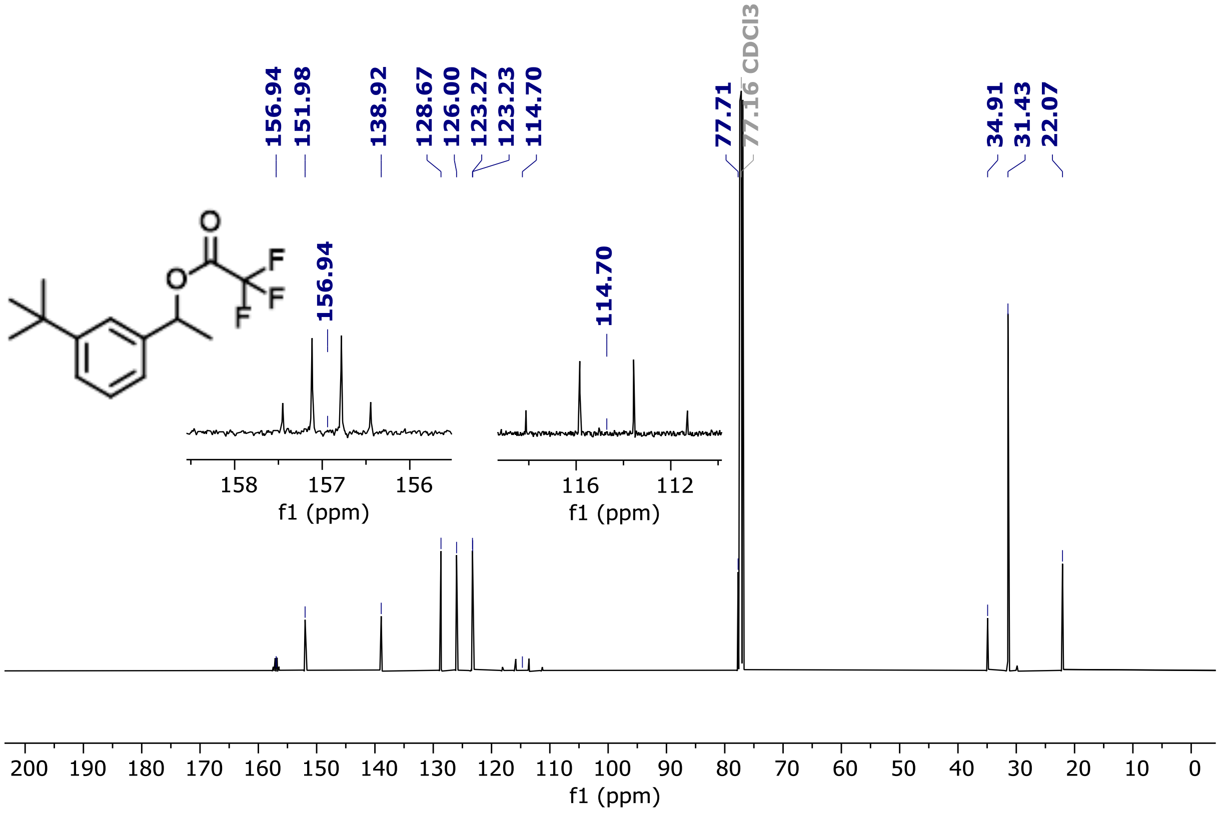

^{19}F NMR (376 MHz, $CDCl_3$)

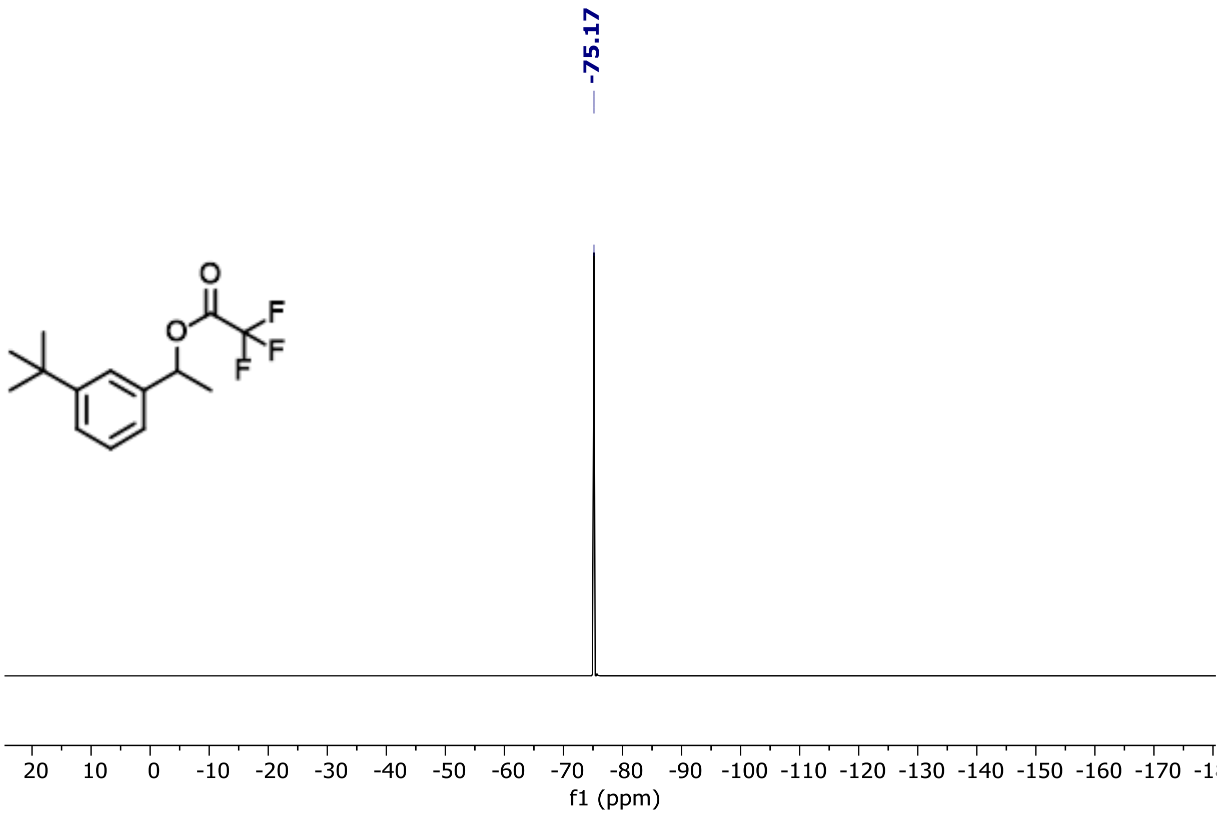

*1-(3-(tert-Butyl)phenyl)ethyl acrylate (**3k**)*

^{1}H NMR (400 MHz, $CDCl_3$)

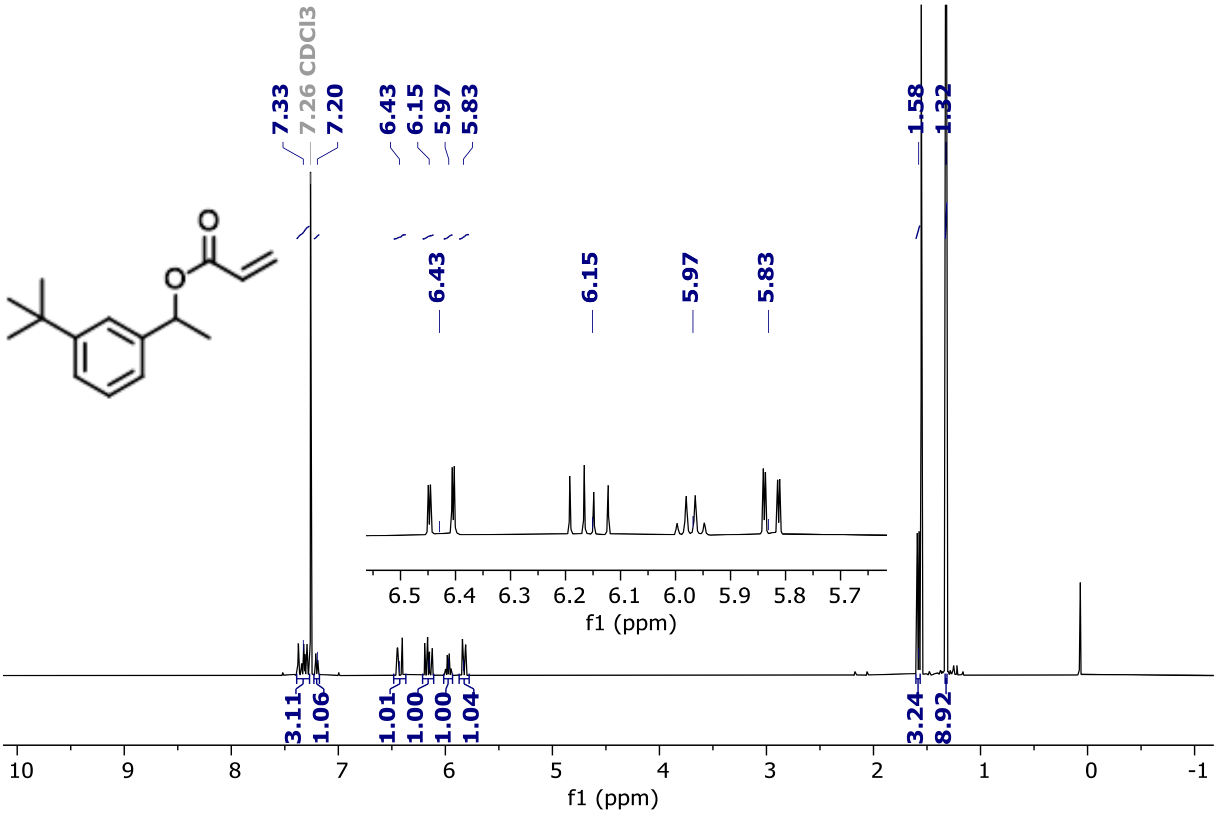

^{13}C NMR (126 MHz, $CDCl_3$)

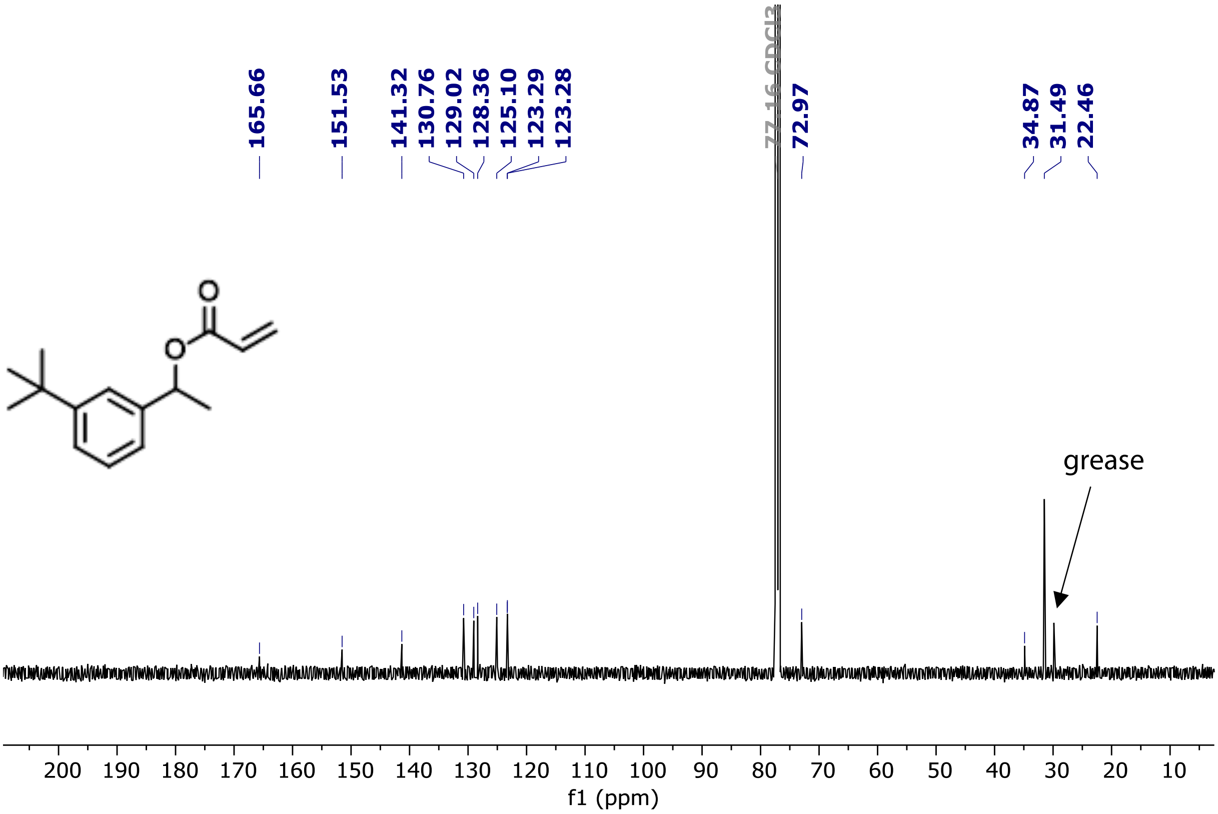

*1-(3-(tert-Butyl)phenyl)ethyl (E)-but-2-enoate (**3l**)*

^{1}H NMR (400 MHz, $CDCl_3$)

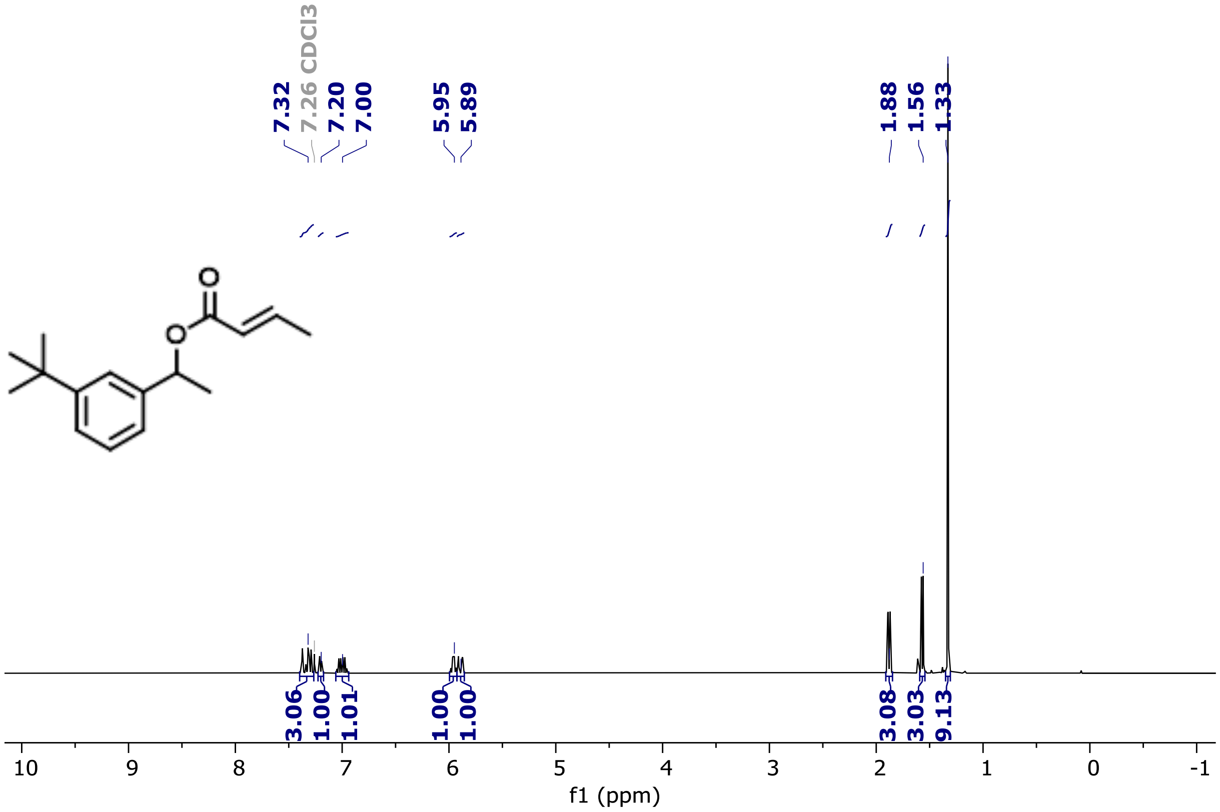

^{13}C NMR (101 MHz, $CDCl_3$)

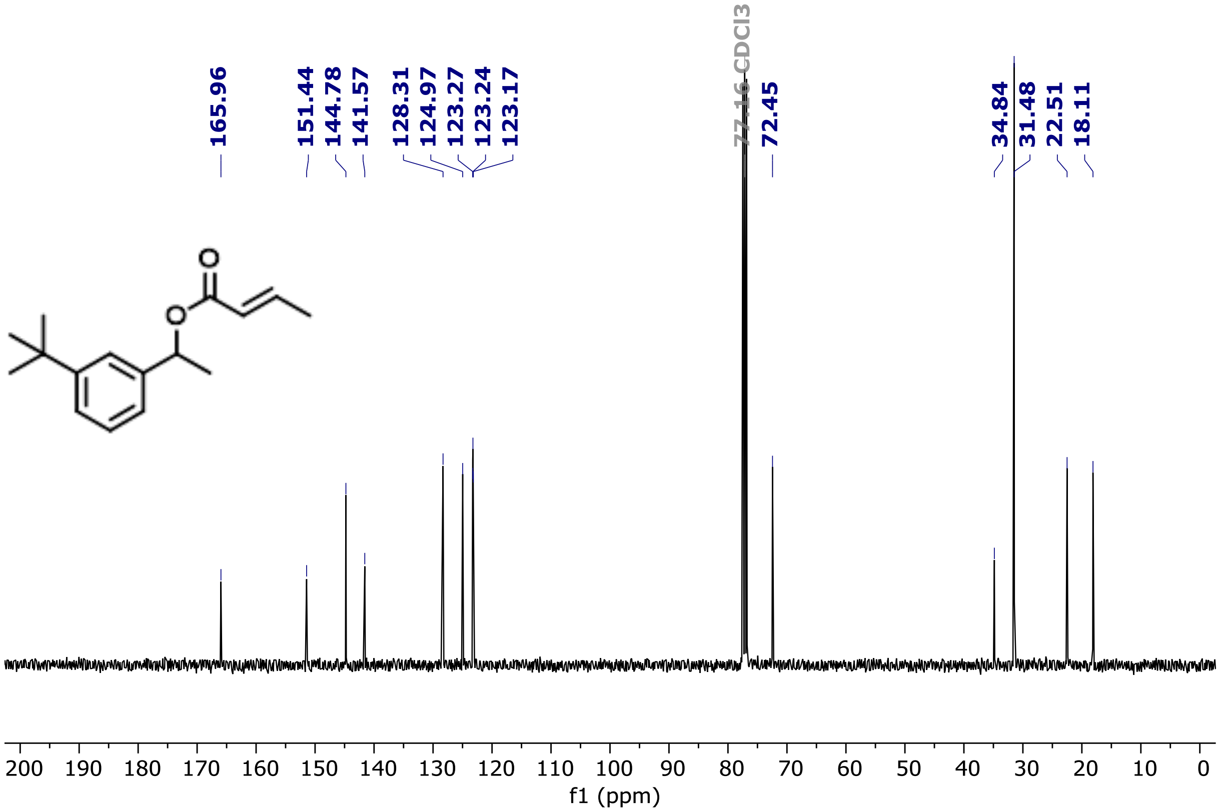

References

(1) Box, J. R.; Atkins, A. P.; Lennox, A. J. J. Direct Electrochemical Hydrodefluorination of Trifluoromethylketones Enabled by Non-Protic Conditions. *Chem. Sci.* **2021**, *12* (30), 10252–

(2) Niakan, M.; Masteri-Farahani, M. Pd–Ni Bimetallic Catalyst Supported on Dendrimer-Functionalized Magnetic Graphene Oxide for Efficient Catalytic Suzuki-Miyaura Coupling Reaction. *Tetrahedron* **2022**, *108*, 132655.

(3) Coumbarides, G. S.; Dingjan, M.; Eames, J.; Flinn, A.; Northen, J. An Efficient Laboratory Synthesis of α-Deuteriated Profens. *J. Label. Compd. Radiopharm.* **2006**, *49* (10), 903–914.

(4) Zhao, J.; Shen, T.; Sun, Z.; Wang, N.; Yang, L.; Wu, J.; You, H.; Liu, Z. Q. Site-Specific Oxidation of (Sp3)C-C(Sp3)/H Bonds by NaNO2/HCl. *Org. Lett.* **2021**, *23* (10), 4057–4061.

(5) Köckinger, M.; Hanselmann, P.; Roberge, D. M.; Geotti-Bianchini, P.; Kappe, C. O.; Cantillo, D. Sustainable Electrochemical Decarboxylative Acetoxylation of Aminoacids in Batch and Continuous Flow. *Green Chem.* **2021**, *23* (6), 2382–2390.

(6) LaMartina, K. B.; Kuck, H. K.; Oglesbee, L. S.; Al-Odaini, A.; Boaz, N. C. Selective Benzylic C–H Monooxygenation Mediated by Iodine Oxides. *Beilstein J. Org. Chem.* **2019**, *15*, 602–609.

(7) Ojha, D. P.; Prabhu, K. R. Regioselective Synthesis of Vinyl Halides, Vinyl Sulfones, and Alkynes: A Tandem Intermolecular Nucleophilic and Electrophilic Vinylation of Tosylhydrazones. *Org. Lett.* **2015**, *17* (1), 18–21.

(8) Rodriguez, A.; Moran, W. J. Palladium-Catalyzed Three-Component Coupling Reactions: 1,1-Difunctionalization of Activated Alkenes. *European J. Org. Chem.* **2009**, *2009* (9), 1313–1316.
